# Aryl versus Alkyl Redox-Active Diazoacetates — Light-Induced C–H Insertion or 1,2-Rearrangement

**DOI:** 10.1021/acs.orglett.3c02055

**Published:** 2023-08-22

**Authors:** João V. Santiago, Katarzyna Orłowska, Michał Ociepa, Dorota Gryko

**Affiliations:** Institute of Organic Chemistry, Polish Academy of Sciences, Kasprzaka 44, 5201-224 Warsaw, Poland

## Abstract

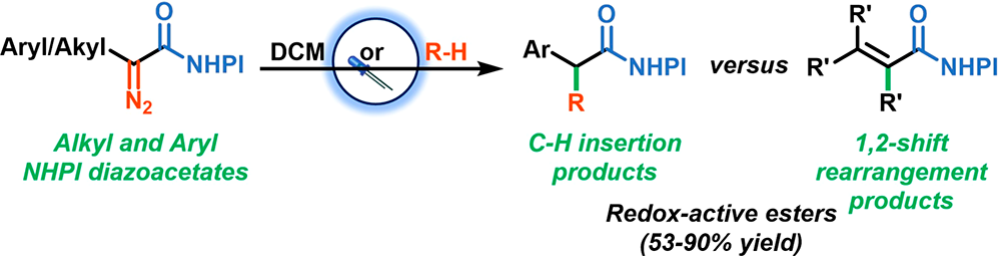

Diazo compounds with redox-active leaving groups are versatile reagents for orthogonal functionalizations, previously utilized in the Rh-catalyzed synthesis of highly substituted cyclopropanes. Photochemical activation of aryl-substituted diazoacetates generates carbenes, whereas redox-active esters can furnish C-radicals via the photoexcitation of EDA complexes. However, the photochemical behavior of these two functionalities, while present in one molecule, remains to be defined. We demonstrate that under light irradiation, reactions occur only on the diazo moiety, leaving the NHPI functionality intact. Not only aryl- but also alkyl-substituted NHPI diazoacetates are activated by blue light; either C–H insertion or the hydrogen/carbon 1,2-rearrangement occurs depending on the aryl/alkyl group.

The use of diazo compounds for C–C bond forming reactions is an important and valuable tool in organic synthesis.^1−6^ Among them, both metal-catalyzed and light-induced cyclopropanation, C–H insertion, rearrangements, and radical coupling reactions are of importance. In metal-catalyzed reactions, carbenoids are formed,^1^ direct photolysis of diazo compounds generates carbenes,^7−10^ while photoredox catalysis leads to radicals.^11−14^ However, the use of visible light in direct photolysis is mainly limited to aryl diazoacetates.

On the other hand, *N*-(acyloxy)phthalimides, known as NHPI esters, are widely employed as substrates for the generation of radicals. Due to their versatility, metal-catalyzed, thermal, and photochemical transformations have been reported.^15,16^ NHPI esters often participates in photoactivated reactions by means of photoinduced single-electron transfer, where this moiety undergoes a reductive fragmentation to form different radical intermediates. Secondary or tertiary C-centered radicals are the most common and very often used in photoinduced cross-coupling reactions.^17,18^

Mendoza and co-workers installed these two moieties in one molecule (Scheme 1A), and the merger of the diazo compound chemistry with the intrinsic characteristic of NHPI esters enabled to obtain chiral, highly substituted cyclopropanes.^19,20^ The telescoped synthesis consists of Rh-catalyzed cyclopropanation, followed by reactions on the redox-active ester moiety. These include light-induced photodecarboxylation driven by an EDA complex between the redox-active moiety and the benzothiazoline,^21^ decarboxylation, decarboxylative borylation, amination, selenylation, alkoxylation, or Giese-type addition performed in the presence of a photocatalyst.^19^ Such difunctionalizations of NHPI diazoacetates allow the formation of two bonds at the same carbon atom. Intriguingly, *despite the ability of these reagents to enable orthogonal functionalizations, their photochemical behavior has not been explored*. Herein, we disclose that not only aryl but also alkyl NHPI diazoacetates are activated by blue light and selectively undergo either C–H insertion or 1,2-H/C rearrangement depending on the substituents at the α-position (Scheme 1B).

**Scheme 1:**
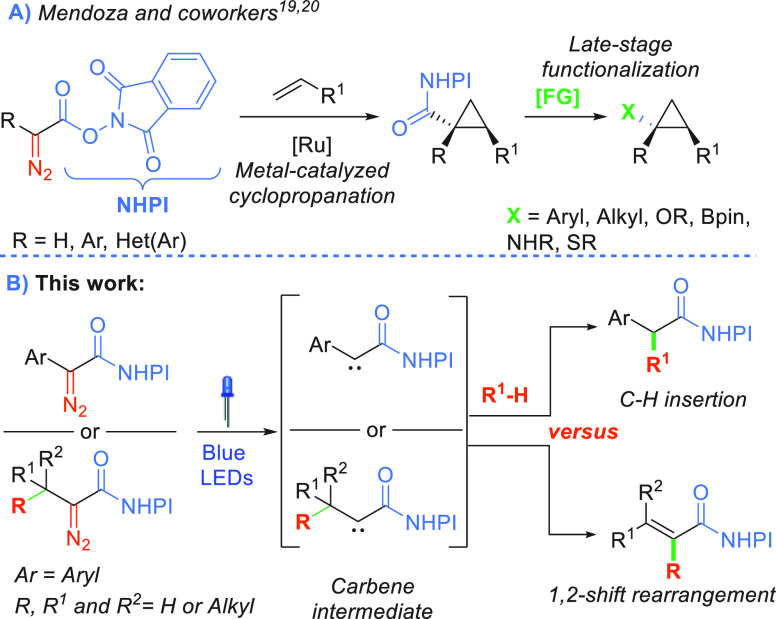
Reactivity of Aryl and Alkyl NHPI Diazoacetates

In the first instance, we prepared aryl- and alkyl-substituted redox-active diazo compounds (Scheme 2).^19,22,23^ To obtain phenyl NHPI diazoacetate **3a** on a gram-scale, 2-oxo-2-phenylacetic acid was treated with *N*-tosylhydrazine, and then, a *one-pot* acylation of *N*-hydroxyphtalimide followed by the release of the diazo group yielded diazoacetate **3a** in 52% yield (Scheme 2A).^20^ Methyl-, ethyl- , and *t*-butyl-substituted NHPI diazo compounds (**4a**,**b**,**e**, respectively) were synthesized according to the reported procedure from glyoxylic acid derivatives.^23,24^ Meanwhile, for the synthesis of diazoacetates **4c**,**d**,**f**, alkylation of diethyl oxalate with Grignard reagents^25^ followed by hydrolysis was required (Scheme 2, for more details see SI).^26^

**Scheme 2:**
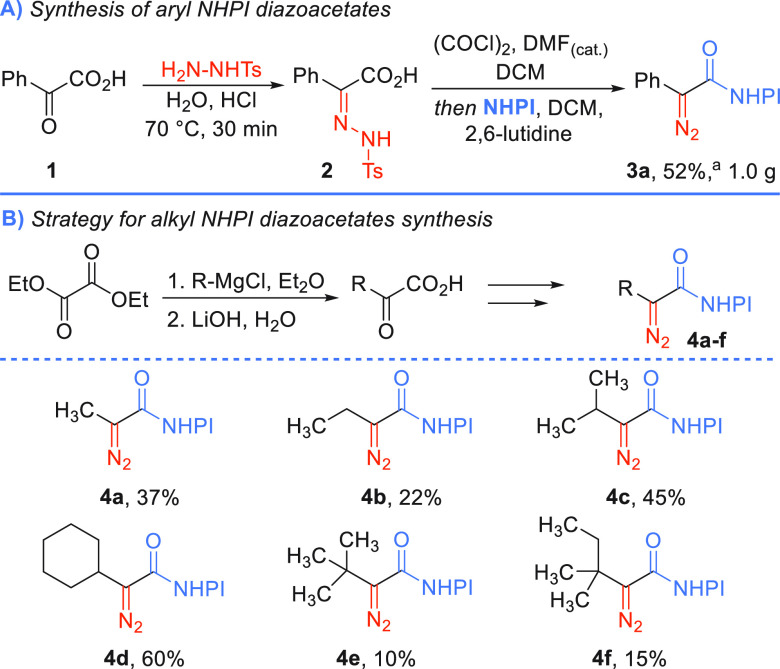
Synthesis of the NHPI Diazoacetates Yield counted from 2-oxo-acids

Simple alkyl NHPI esters do not absorb in the visible region, in contrast to aryl and alkyl diazo acetates (Figure 1). UV/vis spectra of diazo compounds bearing the NHPI moiety revealed that their λ_max_ is hypsochromically shifted. Nevertheless, both alkyl and aryl reagents absorb in the visible region, thus predisposing them to visible-light activation. Photolysis of aryl diazoacetates under blue light irradiation has been indeed broadly investigated,^27^ while solar photochemistry of alkyl counterparts remained underdeveloped even though they display absorption in the violet/blue region.

**Figure 1:**
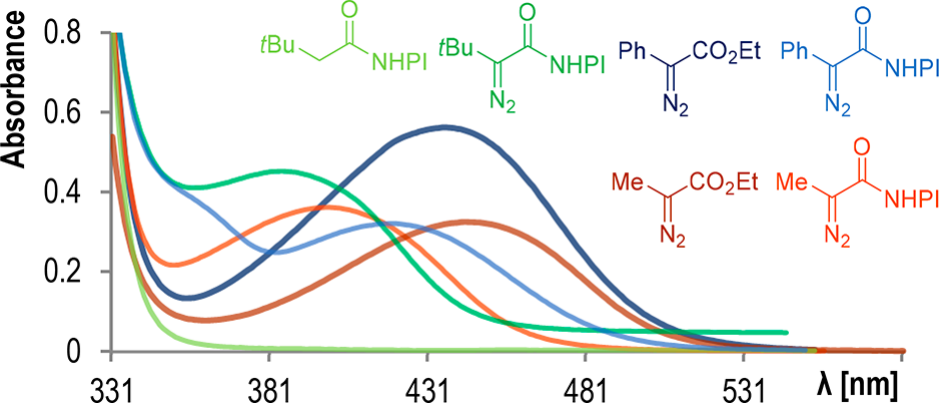
UV–vis spectra of aryl- and alkyl-substituted NHPI diazoacetates.

The reported telescoped synthesis of *cis*-cyclopropanes from aryl NHPI diazoacetates involved Rh-catalyzed cyclopropanation followed by photodecarboxylation.^19,20^ As both diazo and NHPI compounds are photoactive, we wondered whether both can be used in orthogonal photochemical transformations and whether the NHPI moiety remains intact during the generation of carbene species in a photocatalytic manner. To this end, we evaluated the reactivity of diazo compounds **3** and **4** in the model photolytic C–H insertion reaction. The reaction of reagent **3a** with cyclohexane under blue LED irradiation afforded the desired NHPI ester **5a** in only 23% yield with low conversion of the starting material (Table 1, entry 1). After the addition of DCM to solubilize the substrate, the yield increased significantly up to 85% indicating that the NHPI moiety indeed remained intact (entry 2). However, under more intense light irradiation, the NHPI moiety cleavage was observed (entries 4). Control experiments confirmed the crucial role of light in these reactions and of the balance between the solubility of NHPI diazoacetate **3a** and the amount of cyclohexane (entry 5, for details, see SI). For comparison, reactions of simple diazo esters were performed, and products **5a′** and **5a″** were obtained in moderate yields in accordance with the Davies’ protocol (Scheme 3).^7^

**Table 1:**
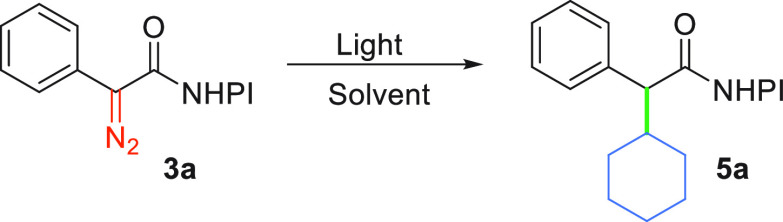
Optimization of the C–H Insertion Reaction with Phenyl NHPI Diazoacetate **3a**^a^

entry	solvent (ratio v/v)	light	yield [%]^b^
1	Cyclohexane	blue (7 W)	23
2	DCM/Cyclohexane (1:1)	blue (7 W)	85
3	DCM/Cyclohexane (1:1)	blue (3 W)	69
4	DCM/Cyclohexane (1:1)	blue (13 W)^c^	60
5	DCM/Cyclohexane (1:1)	none	0

a Reaction conditions: NHPI diazoacetate **3a** (0.1 mmol) in 1.0 mL solvent/cyclohexane (v:v) (0.1 M) irradiated with blue LEDs (450 nm) under an argon atmosphere, 16 h.

b Yield determined by quantitative ^1^H NMR analysis with 1,3,5-trimethoxybenzene as internal standard.

c 445 nm.

With the optimized conditions in hand, we explored generality of the method. The reaction tolerates a wide variety of cycloalkanes, and their ring size has negligible influence (72–84%), even for bulky adamantane (**5d**, 72%, Scheme 3). Dioxane also reacts well furnishing ester **5e** in 57% yield, while for tetrahydrofuran and 1,3-dioxolane, only traces of the C–H insertion product formed; instead, the ether ring opening was observed. Expectedly, in the presence of *n*-pentane, a mixture of products **5f**–**5f″** was observed with predominant substitution at secondary carbons. The electronic character of the phenyl ring in aryl diazoacetates **3** does not have an influence on the product formation, as the yields for respective NHPI esters **5g**–**i** are comparable in all the cases (52–53%). The substitution pattern, however, has an impact; product **5g′** was obtained in a diminished yield as compared to derivative **5g**.

**Scheme 3:**
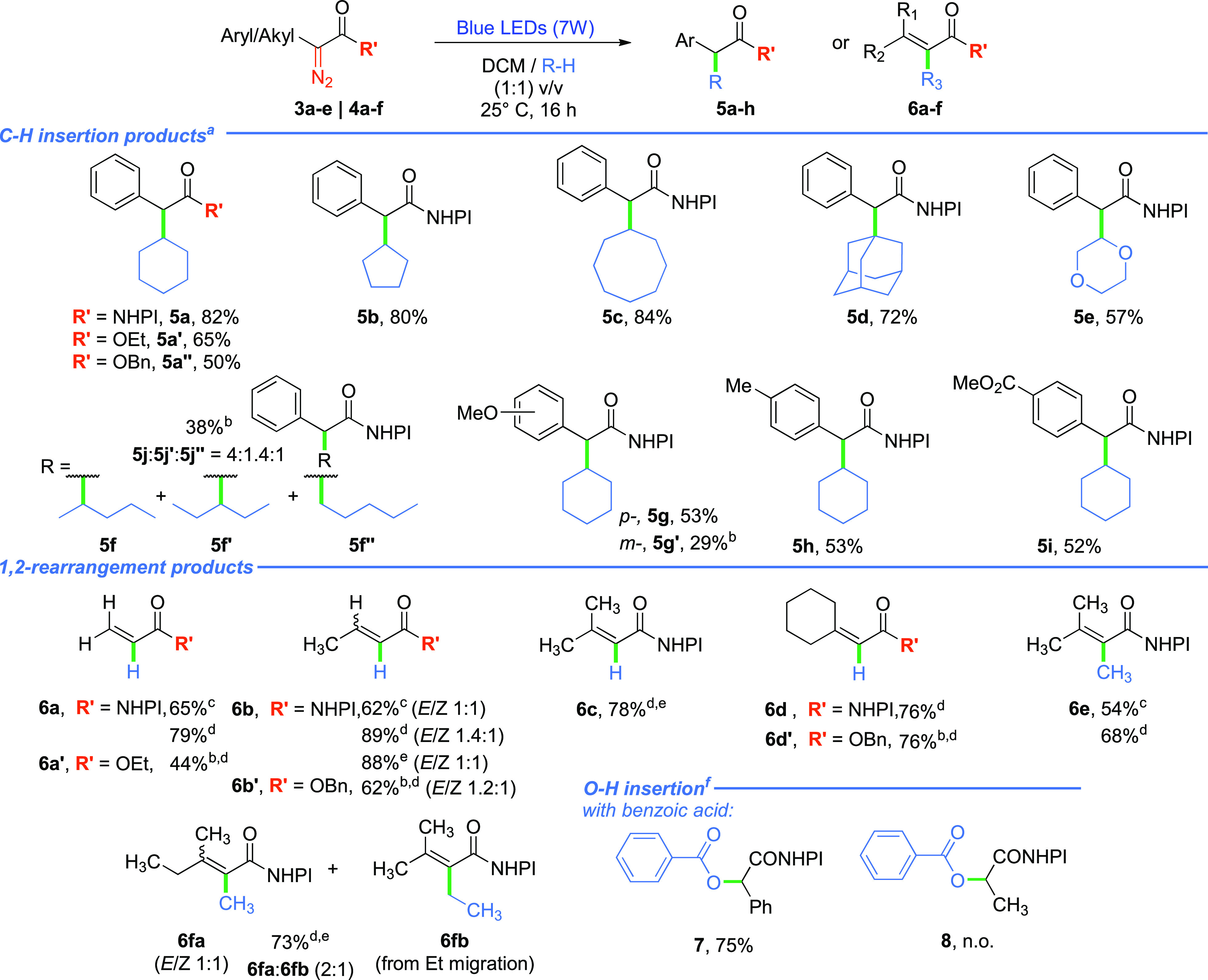
Scope of C–H Insertion and 1,2-Rearrangement Products Reaction conditions: ^a^Blue LEDs (7 W, 455 nm), DCM/R-H (1:1) v/v, 16 h, 0.1 M. ^b^NMR yields. ^c^Blue LEDs (7 W, 455 nm), DCM/cyclohexane (1:1) v/v, 16 h, 0.1 M. ^d^Blue LEDs (7 W, 455 nm), DCM, 16 h, 0.1 M. ^e^Blue LEDs (25 W, 450 nm), DCM, 1.5 h, 0.1 M. ^f^Blue LEDs (7W, 455 nm), acid (5 equiv), DCM, 16 h, 0.1 M. n.o. = not obtained.

Furthermore, the reaction of NHPI diazo ester **3a** with benzoic acid effectively leads to the product **7**. However, attempts to perform O–H (alcohol) and S–H insertions failed to give desired products.

When alkyl NHPI diazoacetate **4a** was exposed to visible-light irradiation under the optimized conditions, the product of type **5** did not form. Instead, the reaction yielded vinyl NHPI ester **6a** in 65% yield, originating from the 1,2-H rearrangement (Scheme 3). When the reaction was performed in only DCM, the yield of ester **6a** increased to 79%. This visible-light-induced process is intramolecular and thus has inherent preference over CH insertion.^28−31^ So far, it has been reported only in metal-catalyzed approaches and observed as a side reaction in photochemical Wolf rearrangements.^32,33^ The 1,2-H shift for ethyl 2-diazopropanoate afforded unsaturated ester **6a′** in 44% yield. For other alkyl NHPI diazoacetates, whenever possible, 1,2-H rearrangement always predominates over 1,2-C rearrangement, as observed for NHPI esters **4b** and **4c**. The photolysis of NHPI diazoacetate **4d** leads to vinyl NHPI ester **6c** in a 78% yield. Even for *tert-*butyl derivative **4e**, the 1,2-migration also occurred instead of the common 1,3-H shift leading to cyclopropanes.^32^ For substrates with different alkyl substituents at the α-position to the carbene moiety, a mixture of compounds **6fa** and **6fb** formed, with the preference for the Me over Et migration (2:1, 75%). The 1,2-H rearrangement is favored over 1,2-C rearrangement due to the higher lability of the hydrogen s orbital to migration reactions and also the more favorable orbital overlap between its orbital and the empty carbene’s p orbital.^34,35^ Other alkyl diazo esters reacted similarly to their NHPI analogues, giving products **6b′** and **6d′**. When subjected to the presence of X–H (X = O, S) reaction partners, no competition of insertion to 1,2-H shift was observed.

In summary, various aryl- and alkyl-substituted redox-active NHPI diazoacetates were synthesized, and their behavior under light irradiation was investigated. For aryl-substituted diazo compounds, selective reactions on the diazo moiety can be performed, as represented by the C–H insertion reaction with cycloalkanes. Alkyl-substituted NHPI diazoacetates can also be photochemically activated. In this case, either 1,2-hydrogen or carbon rearrangement occurs with a predominance for the former one. The presented methodology gives access to a broad variety of NHPI esters that are useful reagents for the generation of C-centered radicals.

## Data Availability

The data underlying this study are available in the published article and its Supporting Information.

## Supporting Information Available

Optimization details, experimental procedures, and characterization data for all new compounds (PDF)
