# Supplementary material for: Aryl versus Alkyl Redox-Active Diazoacetates — Light-Induced C–H Insertion or 1,2-Rearrangement
Source: Org Lett. 2023 Aug 22;25(34):6267–71. doi: 10.1021/acs.orglett.3c02055 (PMC10476266; doi:10.1021/acs.orglett.3c02055)

Supporting Information

Aryl versus Alkyl Redox-Active Diazoacetates – Light Induced C-H Insertion or 1,2-Rearrangement

João V. Santiago, Katarzyna Orłowska, Michał Ociepa and Dorota Gryko*

[a]Institute of Organic Chemistry, Polish Academy of Sciences,
Kasprzaka 44/52, 01-224 Warsaw, Poland

correspondence: dgryko@icho.edu.pl

TABLE OF CONTENTS

1. GENERAL INFORMATION **4**

2. **PHOTOREACTOR SETUPS** **5**

2.1. Constructed photoreactor setup with an aluminum cooling block 5

2.2. UOSlab Miniphoto photoreactor – Commercially available 5

3. SYNTHESIS OF REDOX-ACTIVE NHPI DIAZOACETATES **6**

3.1. General procedure for the synthesis of Glyoxylic acid intermediates (GP1) 6

3.2. General procedure for the synthesis of NHPI-diazoacetates (GP2) 7

3.3. Arylation of 1,3-dioxoisoindolin-2-yl 2-diazoacetate **3b** 11

4. Reactivity of NHPI diazoacetates **13**

4.1. C-H insertion reaction with phenyl NHPI diazoacetate **3a** 13

4.2. General procedure for C-H insertion (GP3) 14

4.3. General procedure for 1,2-shift rearrangement (GP4) 21

General procedure for O-H insertion (GP5) 25

5. REFERENCES **26**

6. UV/VIS SPECTRA **27**

1,3-dioxoisoindolin-2-yl 2-diazo-2-phenylacetate **3a** 27

1,3-dioxoisoindolin-2-yl 2-diazopropanoate **4a** 27

1,3-dioxoisoindolin-2-yl 2-diazobutanoate **4b** 27

1,3-dioxoisoindolin-2-yl 2-diazo-3-methylbutanoate **4c** 28

1,3-dioxoisoindolin-2-yl 2-cyclohexyl-2-diazoacetate **4d** 28

1,3-dioxoisoindolin-2-yl 2-diazo-3,3-dimethylbutanoate **4e** 28

1,3-dioxoisoindolin-2-yl 2-diazo-3,3-dimethylpentanoate **4f** 29

1,3-dioxoisoindolin-2-yl 2-diazo-2-(4-methoxyphenyl)acetate **3c** 29

1,3-dioxoisoindolin-2-yl 2-diazo-2-(*p*-tolyl)acetate **3d** 29

methyl 4-(1-diazo-2-((1,3-dioxoisoindolin-2-yl)oxy)-2-oxoethyl)benzoate **3e** 30

Combined UV/VIS spectra of NHPI diazoacetates 31

7. ^{1}H and ^{13}C NMR DATA **33**

1,3-dioxoisoindolin-2-yl 2-diazo-2-phenylacetate **3a** 33

1,3-dioxoisoindolin-2-yl 2-diazoacetate **3b** 34

1,3-dioxoisoindolin-2-yl 2-diazopropanoate **4a** 35

1,3-dioxoisoindolin-2-yl 2-diazobutanoate **4b** 36

1,3-dioxoisoindolin-2-yl 2-diazo-3-methylbutanoate **4c** 37

1,3-dioxoisoindolin-2-yl 2-cyclohexyl-2-diazoacetate **4d** 38

1,3-dioxoisoindolin-2-yl 2-diazo-3,3-dimethylbutanoate **4e** 39
1,3-dioxoisoindolin-2-yl 2-diazo-3,3-dimethylpentanoate **4f** 40
1,3-dioxoisoindolin-2-yl 2-diazo-2-(4-methoxyphenyl)acetate **3c** 41
1,3-dioxoisoindolin-2-yl 2-diazo-2-(*p*-tolyl)acetate **3d** 42
methyl 4-(1-diazo-2-((1,3-dioxoisoindolin-2-yl)oxy)-2-oxoethyl)benzoate **3e** 43
1,3-dioxoisoindolin-2-yl 2-cyclohexyl-2-phenylacetate **5a** 44
ethyl 2-cyclohexyl-2-phenylacetate **5a’** 45
1,3-dioxoisoindolin-2-yl 2-cyclohexyl-2-phenylacetate **5a’’** 46
1,3-dioxoisoindolin-2-yl 2-cyclopentyl-2-phenylacetate **5b** 47
1,3-dioxoisoindolin-2-yl (S)-2-cyclooctyl-2-phenylacetate **5c** 48
1,3-dioxoisoindolin-2-yl (S)-2-((3S,5S,7S)-adamantan-1-yl)-2-phenylacetate **5d** 49
1,3-dioxoisoindolin-2-yl (R)-2-((R)-1,4-dioxan-2-yl)-2-phenylacetate **5e** 50
1,3-dioxoisoindolin-2-yl 2-cyclohexyl-2-(4-methoxyphenyl)acetate **5f** 51
1,3-dioxoisoindolin-2-yl 2-cyclohexyl-2-(*p*-tolyl)acetate **5g** 52
methyl 4-(1-cyclohexyl-2-((1,3-dioxoisoindolin-2-yl)oxy)-2-oxoethyl)benzoate **5h** 53
1,3-dioxoisoindolin-2-yl acrylate **6a** 54
1,3-dioxoisoindolin-2-yl but-2-enoate **6b** 55
1,3-dioxoisoindolin-2-yl 3-methylbut-2-enoate **6c** 56
1,3-dioxoisoindolin-2-yl 2-cyclohexylideneacetate **6d** 57
1,3-dioxoisoindolin-2-yl 2,3-dimethylbut-2-enoate **6e** 58
1*,3-dioxoisoindolin-2-yl 2,3-dimethylpent-2-enoate (6fa) and 1,3-dioxoisoindolin-2-yl 2-ethyl-3-methylbut-2-enoate* ***(6fb)*** 59
2-((1,3-dioxoisoindolin-2-yl)oxy)-2-oxo-1-phenylethyl benzoate **7** 60

1. GENERAL INFORMATION

All solvents and commercially available reagents were purchased from Sigma-Aldrich, TCI, Acros Organics as reagent grade and were used without further purification, unless otherwise stated. Dry solvents were taken from Solvent Purification System (SPS) or purchased from Sigma Aldrich. Deuterated solvents used were purchased from Eurisotop.

All the photochemical reactions were performed in 10 mL glassy vials sealed with aluminum caps containing a rubber septa. Reactions were monitored by thin layer chromatography (TLC), using 0.20 mm Merck silica plates (60F-254) and visualized using UV-light, potassium permanganate, cerium molybdate or anisaldehyde stain, with heat as a developing agent. Unless otherwise noted, reactions were performed without the exclusion of air or moisture. Colum chromatography was performed on Merck silica gel 60 (230-400 mesh). All yields determined by ^{1}H NMR analysis were obtained using 1,3,5-trimethoxybenzene as the internal standard. Isolated yields refer to spectroscopically (^{1}H NMR) homogeneous materials.

NMR spectra were recorded at ambient temperature (unless otherwise stated) on Bruker 400 MHz or Varian 500, 600 MHz. Chemical shifts are reported in ppm relative to tetramethyl silane signal or a residual undeuterated solvent peak (TMS 0 ppm for ^{1}H and ^{13}C, $CHCl_3$ – 7.26 ppm for ^{1}H and 77.16 ppm for ^{13}C, $(CD_3)_2CO$ – 2.05 ppm for ^{1}H and 29.8 ppm for ^{13}C, CD_2Cl_2 – 5.32 ppm for ^{1}H and 53.5 ppm for ^{13}C, $(CD_3)_2SO$ – 2.49 ppm for ^{1}H and 39.7 ppm for ^{13}C). Multiplicities are given as: singlet (s), doublet (d), triplet (t), quartet (q), multiplet (m) broad singlet (bs).

LR and HRMS. Low-resolution mass spectra (LRMS) were recorded on an Applied Biosystems API 365 mass spectrometer using electrospray ionization (ESI) technique. High-resolution mass spectra (HRMS) were recorded on Waters SYNAPT G2-S HDMS instrument using electrospray ionization (ESI) or atmospheric-pressure chemical ionization (APCI) with time of flight detector (TOF).
Elemental analysis (C, H, N) were performed using a PERKIN-ELMER 240 Elemental Analyzer.
Melting points were recorded on a Marienfeld MPM-H2 melting point apparatus and are uncorrected.

UV-Vis absorption spectra were recorded on UV-3600i Plus UV-Vis-NIR Spectrophotometer.
Fluorescence measurements were performed on Edinburgh Instruments FS5 Spectrofluorometer.
GC-MS analyses were performed using Shimadzu GCMS-QP2010 SE gas chromatograph with FID detector and Zebron ZB 5MSi column.

2. PHOTOREACTOR SETUPS

2.1. Constructed photoreactor setup with an aluminum cooling block

The LED plates are commercially available radiators (Fischer Electronic part no. SK 105 100 SA) with 6 epoxy-glued star-cased 3 W LEDs connected in series (constant current 0.7 A power supply). Reactions were performed under blue light irradiation on a single diode (LT-2855 royal blue, λmax: 446 nm, 7W) per vial with 6 mm distance from the plate. The temperature of LED block was controlled with Huber MiniChiller 300 (T= 29-31 °C) (**Figure S1**).

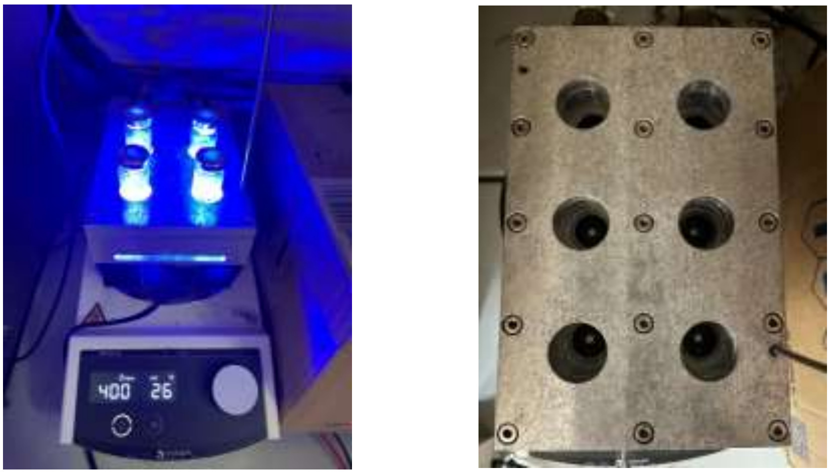

Figure S1 – Constructed photoreactor setup with an aluminum cooling block

2.2. UOSlab Miniphoto photoreactor – Commercially available

Blue (maximum at 450 nm) light was supplied to each reaction vial with the use of 7 LUMINUS LED units (of overall 25 W intensity for 100% power applied). The temperature of LED block was controlled with Huber MiniChiller 300.

3. SYNTHESIS OF REDOX-ACTIVE NHPI DIAZOACETATES

Strategy for the synthesis of NHPI Diazoacetates

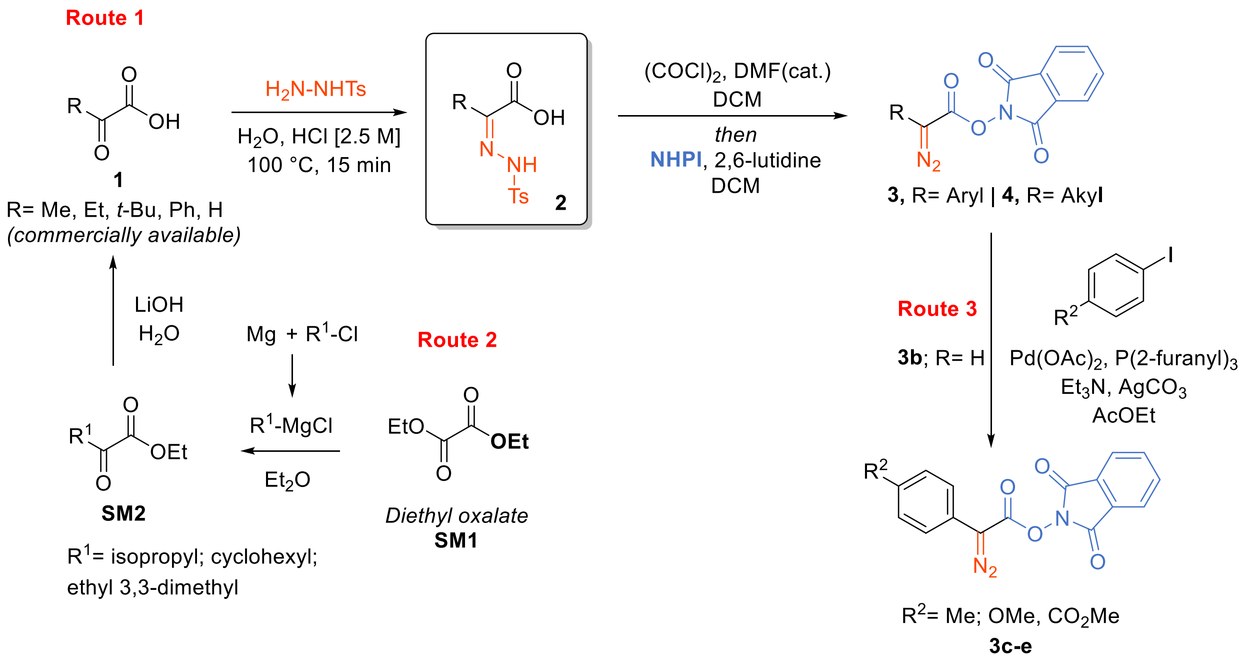

3.1. General procedure for the synthesis of Glyoxylic acid intermediates (GP1)

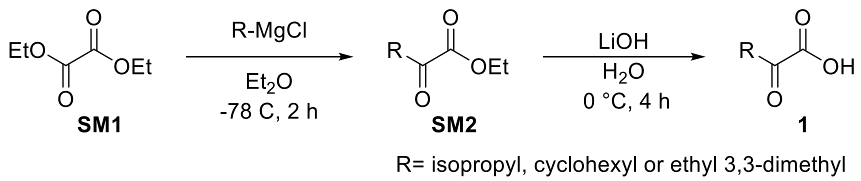

To a 100 mL round-bottom flask containing a solution of diethyl oxalate (1.46 g, 10 mmol) in dry Et_2O (50 m) was added a solution of alkylmagnesium chloride (11 mmol, 1.1 equiv., 1.3 M in ether) dropwise over a period of one hour at -78° C. The mixture was stirred at -78 °C for the next 1.5 h and then poured into a vigorously stirred solution of cold water (40 mL), diethyl ether (50 mL) and concentrated HCl (9 mL). The aqueous phase was separated, and the organic phase was washed with water (50 mL), dried over Na_2SO_4, filtered, and concentrated to furnish viscous oil. To the 25 mL round-bottom flask with the crude from the previous step was added water (4.0 mL), then solution was cooled to 0 °C. Next, LiOH (470 mg, 20 mmol, 2 equiv.) was added in portions, then the reaction was allowed to warm to room temperature and stirred for 4 hours. The reaction was quenched with 2N HCl aqueous solution (14 mL) and extracted with Et_2O (2x 10 mL). The organic phase was dried over Na_2SO_4, filtered, and concentrated to furnish glyoxylic acid derivative, which was used in the next step without further purification.

3.2. General procedure for the synthesis of NHPI-diazoacetates (GP2)

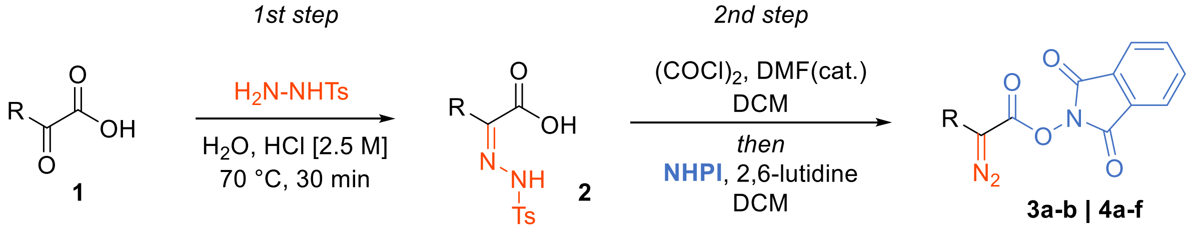

1st step

To 25 mL round-bottom flask were added the glyoxylic acid derivative (6.3 mmol, 1.0 equiv.) and water (6.3 mL). The solution was heated to 60 °C and then *p*-toluenesulfonyl hydrazine (1.17 g, 6.3 mmol, 1.0 equiv.) was added in portions 2 portions over 5 minutes. Next, an aqueous solution of HCl (3.2 mL, 7.9 mmol, 2.5 M) was added dropwise and the reaction was stirred for another 30 minutes. A white precipitate formed* and the reaction mixture was cooled down to room. The white solid was filtered off and washed with cold water. After drying overnight under vacuum, pure *N*-tosylhydrazone was then submitted to the next step without purification.

2nd step

To 150 mL round-bottom flask containing the *N*-tosylhydrazone (6.3 mmol) was added DCM (45 mL) under argon atmosphere. The solution was cooled to 0 °C with an ice-water bath, DMF (19.5 μL, 0.25 mmol, 0.04 equiv.) was added, followed by the dropwise addition of oxalyl chloride (0.65 mL, 7.4 mmol, 1.2 equiv.) over 30 min *(Caution: gas extrusion)*. The reaction was then allowed to warm-up to room temperature and was stirred overnight at room temperature. After that time, the crude reaction mixture was concentrated under vacuum furnishing a brown oil that was submitted to the next step. The solution of the crude in dry DCM (32.0 mL) was cooled down to 0 °C in an ice-water bath, then a briefly sonicated solution of *N*-hydroxyphtalimide (12.6 mmol, 2.0 equiv.) and 2,6-lutidine (2.2 mL, 19 mmol, 3.0 equiv.) in dry DCM (16.0 mL) was added dropwise over a period of 15 min. The reaction was then allowed to warm-up to room temperature and then was stirred overnight at room temperature. After this period, the crude reaction mixture was concentrated under vacuum and purified by column chromatography (silica gel) using hexanes/ethyl acetate, gradually from 98:2 to 85:15 Hex/EA to afford the final product as a yellow solid.

---

*When the precipitate did not form after 30 min, the reaction mixture was cooled down and extracted with DCM (3 x 15 mL). The organic phase was separated, dried over Na_2SO_4, filtered, and concentrated to furnish the *N*-tosylhydrazone in quantitative yield as a viscous oil.

1,3-dioxoisoindolin-2-yl 2-diazo-2-phenylacetate 3a[1]

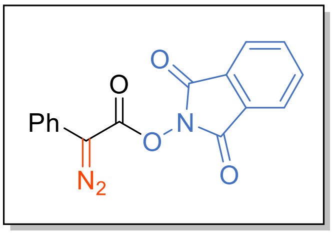

Synthesized according to the general procedure **GP2**. Product isolated as yellow solid (501 mg, 1.63 mmol, 52% yield) by flash chromatography (hexane -AcOEt mixtures, gradually from 0% to 30% of AcOEt in hexane).

^{1}H NMR (500 MHz, $CDCl_3$) δ 8.02–7.88 (m, 2H), 7.89–7.77 (m, 2H), 7.54–7.38 (m, 4H), 7.33–7.20 (m, 1H) ppm.

^{13}C NMR (126 MHz, $CDCl_3$) δ 162.3, 161.4, 135.0, 129.4, 129.1, 127.1, 124.5, 124.2, 123.5 ppm.

1,3-dioxoisoindolin-2-yl 2-diazoacetate 3b[2]

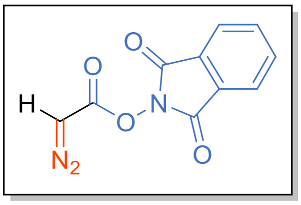

Synthesized according to the general procedure **GP2**. Product isolated as yellow solid (292 mg, 1.26 mmol, 50% yield) by flash chromatography (hexane -AcOEt mixtures, gradually from 0% to 30% of AcOEt in hexane).

^{1}H NMR (600 MHz, $CDCl_3$) δ 7.90 (dd, J = 5.4, 3.1 Hz, 2H), 7.80 (dd, J = 5.5, 3.1 Hz, 2H), 5.18 (bs, 1H) ppm.

^{13}C NMR (151 MHz, $CDCl_3$) δ 163.3, 162.2, 135.0, 129.0, 124.2, 45.2 ppm.

1,3-dioxoisoindolin-2-yl 2-diazopropanoate 4a[3]

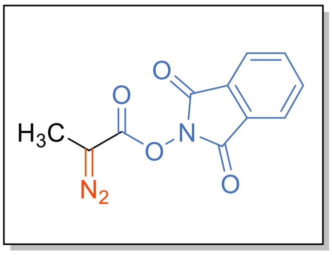

Synthesized according to the general procedure **GP2**. Product isolated as yellow solid (345 mg, 1.41 mmol, 37% yield) by flash chromatography (hexane -AcOEt mixtures, gradually from 0% to 30% of AcOEt in hexane).

^{1}H NMR (500 MHz, $CDCl_3$) δ 7.89 (dd, J = 5.4, 3.1 Hz, 2H), 7.79 (dd, J = 5.5, 3.1 Hz, 2H), 2.09 (bs, 3H) ppm.

^{13}C NMR (126 MHz, $CDCl_3$) δ 162.4, 134.9, 129.1, 124.1, 8.7 ppm.

1,3-dioxoisoindolin-2-yl 2-diazobutanoate 4b[4]

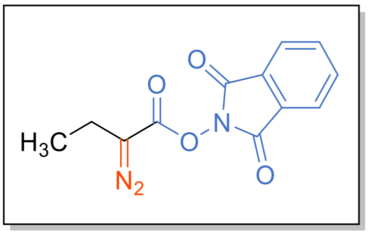

Synthesized according to the general procedure **GP2**. Product isolated as yellow solid (522 mg, 2.0 mmol, 22% yield) by flash chromatography (hexane -AcOEt mixtures, gradually from 0% to 30% of AcOEt in hexane).

^{1}H NMR (500 MHz, $CDCl_3$) δ 7.87 (dd, J = 5.5, 3.1 Hz, 2H), 7.77 (dd, J = 5.5, 3.1 Hz, 2H), 2.47 (bs, 2H), 1.21 (t, J = 7.5 Hz, 3H) ppm.

^{13}C NMR (126 MHz, $CDCl_3$) δ 162.3, 134.9, 129.0, 124.1, 17.0, 11.8 ppm.

1,3-dioxoisoindolin-2-yl 2-diazo-3-methylbutanoate 4c

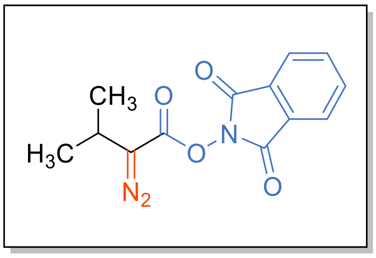

Synthesized according to the general procedure **GP2**. Product isolated as yellow solid (597 mg, 2.18 mmol, 45% yield) by flash chromatography (hexane -AcOEt mixtures, gradually from 0% to 30% of AcOEt in hexane).

^{1}H NMR (500 MHz, $CDCl_3$) δ 7.91 – 7.86 (m, 2H), 7.79 (dd, J = 5.5, 3.1 Hz, 2H), 2.85 (bs, 1H), 1.23 (d, J = 6.9 Hz, 6H) ppm.

^{13}C NMR (126 MHz, $CDCl_3$) δ 162.4, 134.9, 129.1, 124.1, 24.0, 20.6 ppm.

HRMS (EI) m/z: [M]+ calculated for $C_{13}H_{11}N_3O_4$ 273,0750; found 273.0753.

1,3-dioxoisoindolin-2-yl 2-cyclohexyl-2-diazoacetate 4d

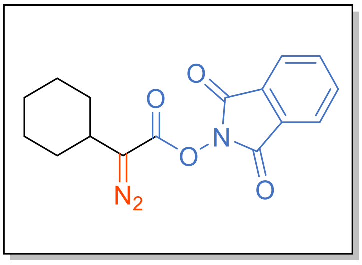

Synthesized according to the general procedure **GP2**. Product isolated as yellowish solid (1.74 g, 5.55 mmol, 60% yield) by flash chromatography (hexane -AcOEt mixtures, gradually from 0% to 30% of AcOEt in hexane).

^{1}**H NMR** (500 MHz, $CDCl_3$) δ 7.89 (dd, *J* = 5.5, 3.1 Hz, 2H), 7.78 (dd, *J* = 5.5, 3.1 Hz, 2H), 2.62–2.32 (m, 1H), 2.12–1.91 (m, 2H), 1.89–1.76 (m, 2H), 1.76–1.62 (m, 1H), 1.46–1.30 (m, 2H), 1.30–1.10 (m, 3H) ppm.

^{13}**C NMR** (126 MHz, $CDCl_3$) δ 162.4, 134.9, 129.1, 124.1, 32.4, 30.9, 26.1, 25.7 ppm.

HRMS (EI) m/z: $[M+Na]^+$ calculated for $C_{16}H_{15}N_3NaO_4$ 336.0960; found 336.0963.

1,3-dioxoisoindolin-2-yl 2-diazo-3,3-dimethylbutanoate 4e

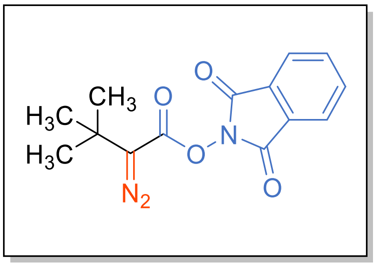

Synthesized according to the general procedure **GP2**. Product isolated as yellow solid (167 mg, 0.58 mmol, 10% yield) by flash chromatography (hexane -AcOEt mixtures, gradually from 0% to 30% of AcOEt in hexane).

^{1}**H NMR** (500 MHz, $CDCl_3$) δ 7.89 (dd, *J* = 5.4, 3.1 Hz, 2H), 7.78 (dd, *J* = 5.5, 3.1 Hz, 2H), 1.32 (s, 9H) ppm.

^{13}**C NMR** (126 MHz, $CDCl_3$) δ 162.5, 134.8, 129.1, 124.1, 30.6, 28.7 ppm.

HRMS (ESI) m/z: $[M+Na]^+$ calculated for $C_{14}H_{13}N_3NaO_4$ 310.0804; found 310.0805.

1,3-dioxoisoindolin-2-yl 2-diazo-3,3-dimethylpentanoate 4f

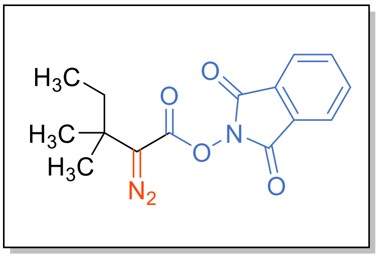

Synthesized according to the general procedure **GP2**. Product isolated as yellow solid (313 mg, 1.0 mmol, 15% yield) by flash chromatography (hexane -AcOEt mixtures, gradually from 0% to 30% of AcOEt in hexane).

^{1}**H NMR** (500 MHz, $CDCl_3$) δ 7.88 (dd, *J* = 5.5, 3.1 Hz, 2H), 7.78 (dd, *J* = 5.5, 3.1 Hz, 2H), 1.64 (q, *J* = 7.5 Hz, 2H), 1.26 (s, 6H), 0.93 (t, *J* = 7.5 Hz, 3H) ppm.

^{13}**C NMR** (126 MHz, $CDCl_3$) δ 162.5, 162.1, 134.8, 129.1, 124.1, 46.3, 34.4, 33.3, 26.2, 9.3 ppm.

HRMS (ESI) m/z: $[M+Na]^+$ calculated for $C_{15}H_{15}N_3NaO_4$ 324.0960; found 324.0959.

3.3. Arylation of 1,3-dioxoisoindolin-2-yl 2-diazoacetate 3b

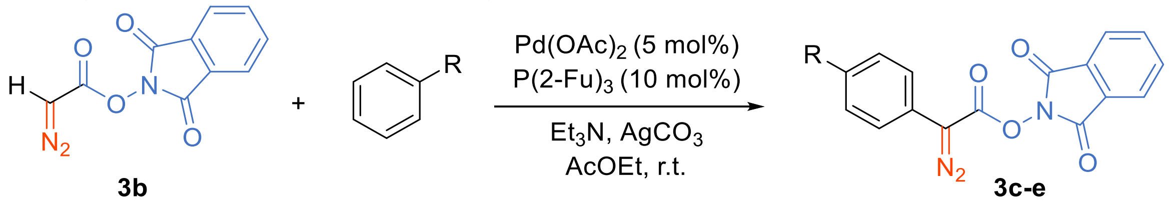

The NHPI-diazoacetates **3c-e** were synthesized according to literature procedure reported by Mendoza and coworkers[2] by a palladium-catalyzed arylation protocol[1] from the diazo **3b**, with the respective aryl iodide.

1,3-dioxoisoindolin-2-yl 2-diazo-2-(4-methoxyphenyl)acetate 3c[2]

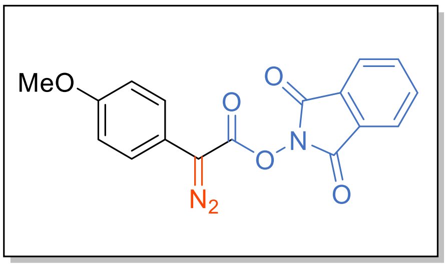

Product isolated as yellow solid (25 mg, 0.08 mmol, 38% yield) by flash chromatography (hexane - AcOEt mixtures, gradually from 0% to 30% of AcOEt in hexane).

^{1}H NMR (500 MHz, $CDCl_3$) δ 7.91 (dd, *J* = 5.5, 3.0 Hz, 2H), 7.80 (dd, *J* = 5.5, 3.1 Hz, 2H), 7.42–7.35 (m, 2H), 7.00–6.93 (m, 2H), 3.81 (s, 3H) ppm.

^{13}C NMR (126 MHz, $CDCl_3$) δ 162.3, 161.9, 159.1, 135.0, 129.0, 126.8, 124.2, 115.0, 114.8, 55.5 ppm.

1,3-dioxoisoindolin-2-yl 2-diazo-2-(p-tolyl)acetate 3d[2]

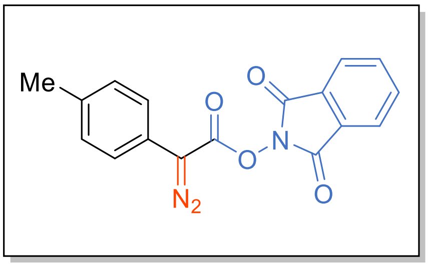

Product isolated as yellow solid (45.0 mg, 0.14 mmol, 70% yield) by flash chromatography (hexane - AcOEt mixtures, gradually from 0% to 30% of AcOEt in hexane).

^{1}H NMR (400 MHz, $CDCl_3$) δ 7.90 (dd, *J* = 5.4, 3.1 Hz, 2H), 7.79 (dd, *J* = 5.5, 3.1 Hz, 2H), 7.34 (d, *J* = 8.3 Hz, 2H), 7.22 (d, *J* = 8.1 Hz, 2H), 2.34 (s, 3H) ppm.

^{13}C NMR (101 MHz, $CDCl_3$) δ 162.3, 137.2, 135.0, 130.1, 129.0, 124.7, 124.1, 120.1, 21.1 ppm.

Methyl 4-(1-diazo-2-((1,3-dioxoisoindolin-2-yl)oxy)-2-oxoethyl)benzoate 3e[2]

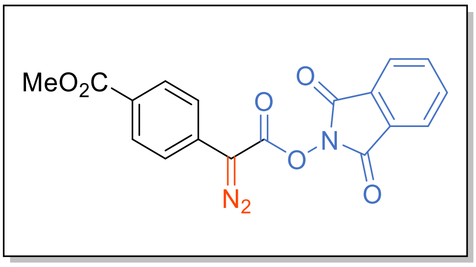

Product isolated as yellow solid (38 mg, 0.10 mmol, 52% yield) by flash chromatography (hexane - AcOEt mixtures, gradually from 0% to 30% of AcOEt in hexane).

^{1}H NMR (500 MHz, $CDCl_3$) δ 8.06 (d, *J* = 8.7 Hz, 2H), 7.92 (dd, *J* = 5.5, 3.1 Hz, 2H), 7.82 (dd, *J* = 5.5, 3.1 Hz, 2H), 7.54 (d, *J* = 8.7 Hz, 2H), 3.91 (s, 3H) ppm.

^{13}C NMR (126 MHz, $CDCl_3$) δ 166.5, 162.1, 160.7, 135.1, 130.5, 128.9 (2C), 128.4, 124.3, 123.4, 62.4, 52.3 ppm.

1,3-dioxoisoindolin-2-yl 2-diazo-2-(3-methoxyphenyl)acetate 3f[1]

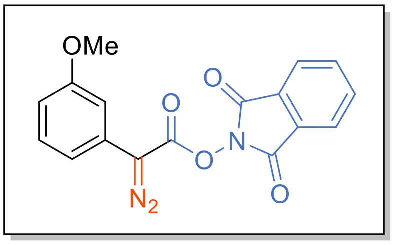

Synthesized according to the general procedure **GP2**. Product isolated as yellow solid (23 mg, 0.07 mmol, 25% yield) by flash chromatography (hexane -AcOEt mixtures, gradually from 0% to 30% of AcOEt in hexane).

^{1}H NMR (500 MHz, $CDCl_3$) δ 7.94 – 7.86 (m, 2H), 7.83 – 7.77 (m, 2H), 7.32 (t, *J* = 8.1 Hz, 1H), 7.10 (bs, 1H), 6.98 (d, *J* = 7.9 Hz, 1H), 6.79 (d, *J* = 7.5 Hz, 1H), 3.80 (s, 3H) ppm.

^{13}C NMR (126 MHz, $CDCl_3$) δ 162.3, 161.3, 160.4, 135.0, 130.3, 129.0, 124.9, 124.2, 116.4, 112.9, 110.1, 55.4 ppm.

4. Reactivity of NHPI diazoacetates

4.1. C-H insertion reaction with phenyl NHPI diazoacetate **3a**

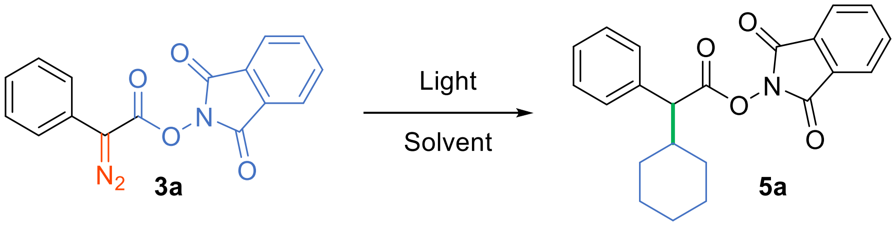

Entry	Solvent (ratio v/v)	Light	Time (h)	Yield (%)[a]
1	Cyclohexane	Violet (13 W)	16	14
2	DCM/Cyclohexane (1:1)	Violet (13 W)	16	26
3	DCM/Cyclohexane (1:1)	Blue (13 W)	16	60
4	DCM/Cyclohexane (1:1)	Blue (25 W)	16	43
**5**	**DCM/Cyclohexane (1:1)**	**Blue (7 W)**	**16**	**85 [82]**
6[b]	DCM/Cyclohexane (1:1)	Blue (7 W)	16	67
7	DCM/Cyclohexane (1:1)	Blue (3 W)	16	69
8	DCM/Cyclohexane (3:1)	Blue (7 W)	16	60
9	DCM/Cyclohexane (9:1)	Blue (7 W)	16	56
10	Cyclohexane	Blue (7 W)	16	23
11	DCM/Cyclohexane (1:1)	Blue (7 W)	1	67 [63]
12	DCE/Cyclohexane (1:1)	Blue (7 W)	16	58
13	MeCN/Cyclohexane (1:1)	Blue (7 W)	16	21
14	$CHCl_3$/Cyclohexane (1:1)	Blue (7 W)	16	74
15	AcOEt/Cyclohexane (1:1)	Blue (7 W)	16	19

Reaction conditions: NHPI diazoacetate (0.1 mmol) in 1.0 mL solvent/cyclohexane (v:v) (0.1 M) irradiated with LED (Violet = 405 nm/ Blue= 450 nm) under Argon atmosphere | [a] yield determined by quantitative ^{1}H NMR analysis with 1,3,5-trimethoxybenzene as internal standard | [b] DCM pure for analysis (not dry) | DCM = dichloromethane; DCE = 1,2-dichloroethane; [ ]= isolated yields.

4.2. General procedure for C-H insertion (GP3)

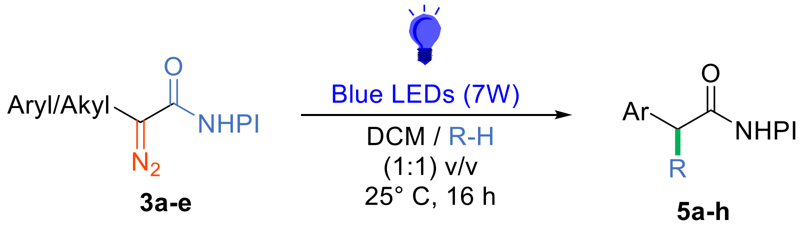

A glass vial equipped with a stirring bar was charged with NHPI diazoacetate **3a** (31 mg, 0.1 mmol), then sealed with an aluminum cap with a rubber septum. Dry DCM (0.5 mL) and cyclohexane (0.5 ml) were added into the vial under the argon atmosphere, followed by the oxygen removal from the solution by freeze-pump-thaw technique. The reaction vial was placed in a photoreactor and was irradiated with blue LED (450 nm, 7 W) for 16 h. After that time, the crude reaction mixture was concentrated under vacuum and purified by column chromatography (silica gel) using hexanes/ethyl acetate to afford the final product.

1,3-dioxoisoindolin-2-yl 2-cyclohexyl-2-phenylacetate 5a[5,6]

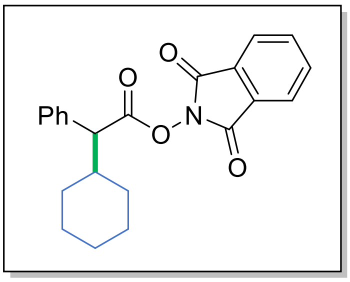

Synthesized according to the general procedure **GP3**. Product isolated as white solid (30 mg, 0.08 mmol, 82% yield) by flash chromatography (hexane -AcOEt mixtures, gradually from 0% to 30% of AcOEt in hexane).

^{1}H NMR (500 MHz, $CDCl_3$) δ 7.84 (m, 2H), 7.75 (dd, *J* = 5.5, 3.1 Hz, 2H), 7.41–7.34 (m, 4H), 7.34–7.28 (m, 1H), 3.64 (d, *J* = 9.8 Hz, 1H), 2.19–2.04 (m, 2H), 1.89–1.78 (m, 1H), 1.75–1.61 (m, 2H), 1.51–1.41 (m, 1H), 1.40–1.28 (m, 1H), 1.29–1.12 (m, 3H), 0.96–0.81 (m, 1H) ppm.

^{13}C NMR (126 MHz, $CDCl_3$) δ 170.2, 162.0, 135.7, 134.8, 129.1, 128.8 (2C), 128.0, 124.0, 55.7, 41.6, 31.7, 30.5, 26.3, 26.1, 26.1 ppm.

Ethyl 2-cyclohexyl-2-phenylacetate 5a'[7]

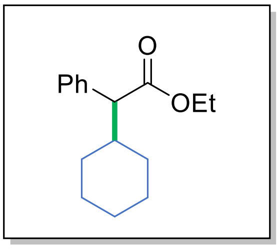

Synthesized according to the general procedure **GP3**. Product isolated as viscous oil (30 mg, 0.07 mmol, 65% yield) by flash chromatography (hexane -AcOEt mixtures, gradually from 0% to 30% of AcOEt in hexane).

^{1}H NMR (500 MHz, $CDCl_3$) δ 7.94 (d, *J* = 7.3 Hz, 1H), 7.58 – 7.50 (m, 2H), 7.38 – 7.25 (m, 2H), 4.28 (q, *J* = 7.1 Hz, 2H), 3.46 (bs, 1H), 2.14 – 2.02 (m, 1H), 2.00 – 1.85 (m, 1H), 1.75 (s, 1H), 1.63 (s, 1H), 1.41 – 1.22 (m, 7H), 1.20 – 0.97 (m, 2H), 0.90 – 0.77 (m, 1H) ppm.

^{13}C NMR (126 MHz, $CDCl_3$) δ 171.9, 136.4, 132.3, 128.8, 127.8, 62.1, 56.4, 41.4, 31.9, 30.5, 26.3, 26.0, 22.8, 14.0 ppm.

1,3-dioxoisoindolin-2-yl 2-cyclohexyl-2-phenylacetate 5a''

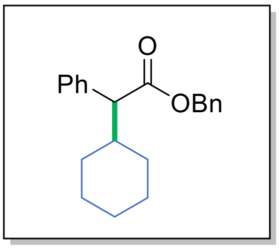

Synthesized according to the general procedure **GP3**. Product isolated as white solid (15.3 mg, 0.05 mmol, 50% yield) by flash chromatography (hexane -AcOEt mixtures, gradually from 0% to 30% of AcOEt in hexane).

^{1}H NMR (500 MHz, $CDCl_3$) δ 7.35 – 7.27 (m, 6H), 7.28 – 7.21 (m, 1H), 5.14 (d, *J* = 12.5 Hz, 1H), 5.03 (d, *J* = 12.5 Hz, 1H), 3.29 (d, *J* = 10.7 Hz, 1H), 2.09 – 1.99 (m, 1H), 1.83 – 1.75 (m, 1H), 1.75 – 1.67 (m, 1H), 1.65 – 1.56 (m, 2H), 1.36 – 1.23 (m, 2H), 1.18 – 1.09 (m, 1H), 1.11 – 0.99 (m, 1H), 0.80 – 0.68 (m, 1H).

ppm.

^{13}C NMR (126 MHz, $CDCl_3$) δ 173.9, 137.9, 136.2, 128.9, 128.6, 128.6, 128.2, 128.1, 127.3, 66.4, 59.0, 41.2, 32.1, 30.6, 26.1, 26.1 ppm.

1,3-dioxoisoindolin-2-yl 2-cyclopentyl-2-phenylacetate 5b[6]

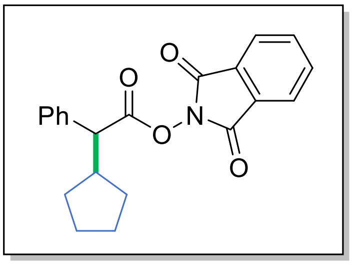

Synthesized according to the general procedure **GP3**. Product isolated as white solid (28 mg, 0.08 mmol, 80% yield) by flash chromatography (hexane -AcOEt mixtures, gradually from 0% to 30% of AcOEt in hexane).

^{1}H NMR (500 MHz, $CDCl_3$ δ 7.90–7.80 (m, 2H), 7.76 (dd, *J* = 5.5, 3.1 Hz, 2H), 7.46–7.34 (m, 4H), 7.34–7.28 (m, 1H), 3.68 (d, *J* = 10.8 Hz, 1H), 2.71–2.55 (m, 1H), 2.16–2.07 (m, 1H), 1.81–1.59 (m, 3H), 1.58–1.46 (m, 3H), 1.21–1.07 (m, 1H) ppm.

^{13}C NMR (126 MHz, $CDCl_3$) δ 170.2, 162.0, 136.9, 134.8, 129.1, 128.9, 128.5, 128.0, 124.0, 54.7, 44.0, 31.5, 31.0, 25.3, 24.9 ppm.

1,3-dioxoisoindolin-2-yl 2-cyclooctyl-2-phenylacetate 5c

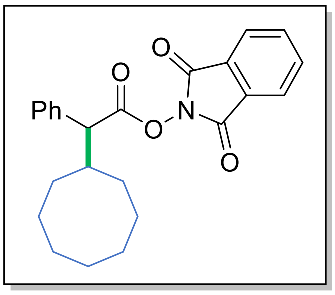

Synthesized according to the general procedure **GP3**. Product isolated as white solid 33 mg (0.08 mmol, 84% yield) by flash chromatography (hexane -AcOEt mixtures, gradually from 0% to 30% of AcOEt in hexane).

m.p. 142 – 144 °C

^{1}H NMR (500 MHz, $CDCl_3$) δ 7.88–7.79 (m, 2H), 7.78–7.71 (m, 2H), 7.45–7.34 (m, 4H), 7.34–7.28 (m, 1H), 3.70 (d, *J* = 10.5 Hz, 1H), 2.50–2.34 (m, 1H), 1.99–1.89 (m, 1H), 1.85–1.74 (m, 1H), 1.71–1.47 (m, 9H), 1.47–1.38 (m, 1H), 1.37–1.28 (m, 1H), 1.24–1.13 (m, 1H) ppm.

^{13}C NMR (126 MHz, $CDCl_3$) δ 170.5, 162.0, 136.4, 134.8, 129.1, 128.9 (2C), 128.0, 124.0, 56.1, 41.0, 31.0, 29.6, 27.0, 26.9, 26.6, 25.8, 25.1 ppm.

HRMS (ESI) m/z: [M+Na]+ calculated for $C_{24}H_{25}NNaO_4$ 414.1681; found 414.1683.

1,3-dioxoisoindolin-2-yl 2-((3r,5r,7r)-adamantan-1-yl)-2-phenylacetate 5d

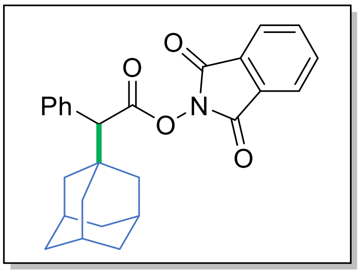

Synthesized according to the general procedure **GP3**. Product isolated as white solid (30 mg, 0.07 mmol, 72% yield) by flash chromatography (hexane -AcOEt mixtures, gradually from 0% to 30% of AcOEt in hexane).

m.p. 208 – 210 °C

^{1}H NMR (500 MHz, $CDCl_3$) δ 7.86 (bs, 2H), 7.81–7.74 (m, 2H), 7.43–7.29 (m, 5H), 3.64 (s, 1H), 2.01 (s, 3H), 1.78–1.70 (m, 6H), 1.70–1.58 (m, 6H) ppm.

^{13}C NMR (126 MHz, $CDCl_3$) δ 168.7, 162.1, 134.8, 132.9, 130.2, 129.2, 128.1, 127.9, 124.0, 59.9, 39.9, 37.0, 36.8, 28.8 ppm.

HRMS (ESI) m/z: $[M+Na]^+$ calculated for $C_{26}H_{25}NNaO_4$ 438.1681; found 438.1683.

1,3-dioxoisoindolin-2-yl 2-(1,4-dioxan-2-yl)-2-phenylacetate 5e

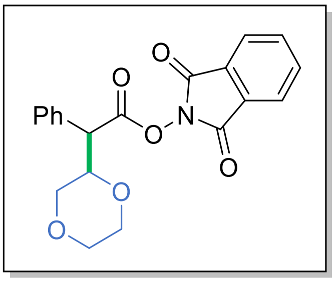

Synthesized according to the general procedure **GP3**. Product isolated as white solid (21 mg, 0.06 mmol, 57% yield) by flash chromatography (hexane -AcOEt mixtures, gradually from 0% to 30% of AcOEt in hexane).

m.p. 196 – 199 °C

^{1}H NMR (500 MHz, $CDCl_3$) δ 7.85 (dd, *J* = 5.5, 3.1 Hz, 2H), 7.77 (dd, *J* = 5.5, 3.1 Hz, 2H), 7.69–7.62 (m, 2H), 7.48–7.42 (m, 2H), 7.41–7.34 (m, 1H), 4.15 (ddd, *J* = 13.9, 7.3, 1.4 Hz, 1H), 4.04 (ddd, *J* = 13.9, 5.1, 1.5 Hz, 1H), 3.98–3.88 (m, 2H), 3.88–3.74 (m, 2H), 3.14 (dd, *J* = 16.1, 7.8 Hz, 1H), 2.70 (dd, *J* = 16.1, 7.6 Hz, 1H) ppm.

^{13}C NMR (126 MHz, $CDCl_3$) δ 170.1, 161.7, 139.7, 134.9, 129.1, 128.9, 128.8, 126.0, 124.1, 83.7, 71.8, 69.0, 65.3, 42.3 ppm.

HRMS (ESI) m/z: $[M+Na]^+$ calculated for $C_{20}H_{17}NNaO_6$ 390.0954; found 390.0953.

1,3-dioxoisoindolin-2-yl 2-cyclohexyl-2-(4-methoxyphenyl)acetate 5f

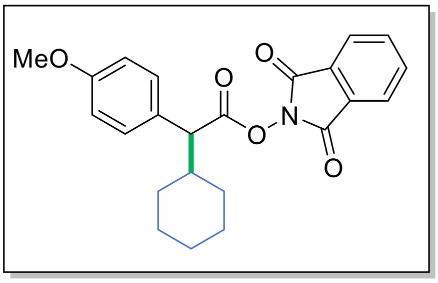

Synthesized according to the general procedure **GP3**. Product isolated as white semisolid (21 mg (0.05 mmol, 53% yield) by flash chromatography (hexane -AcOEt mixtures, gradually from 0% to 30% of AcOEt in hexane).

^{1}H NMR (400 MHz, $CDCl_3$) δ 7.84 (dd, *J* = 5.5, 3.1 Hz, 2H), 7.76 (dd, *J* = 5.5, 3.1 Hz, 2H), 7.31–7.27 (m, 2H), 6.92–6.86 (m, 2H), 3.81 (s, 3H), 3.58 (d, *J* = 9.9 Hz, 1H), 2.12–1.99 (m, 2H), 1.86–1.77 (m, 1H), 1.70–1.62 (m, 2H), 1.51–1.43 (m, 1H), 1.40–1.30 (m, 1H), 1.30–1.10 (m, 3H), 0.93–0.78 (m, 1H) ppm.

^{13}C NMR (101 MHz, $CDCl_3$) δ 170.4, 159.4, 134.8, 129.9, 129.2, 127.8, 124.0, 114.3, 55.4, 54.9, 41.6, 31.8, 30.5, 26.4, 26.1 (2C) ppm.

HRMS (ESI) m/z: $[M+Na]^+$ calculated for $C_{23}H_{23}NNaO_5$ 416.1474; found 416.1477.

1,3-dioxoisoindolin-2-yl 2-cyclohexyl-2-(3-methoxyphenyl)acetate 5f’

HRMS (ESI) m/z: [M+Na]$^+$ calculated for $C_{23}H_{23}NNaO_5$ 416.1474; found 416.1475.

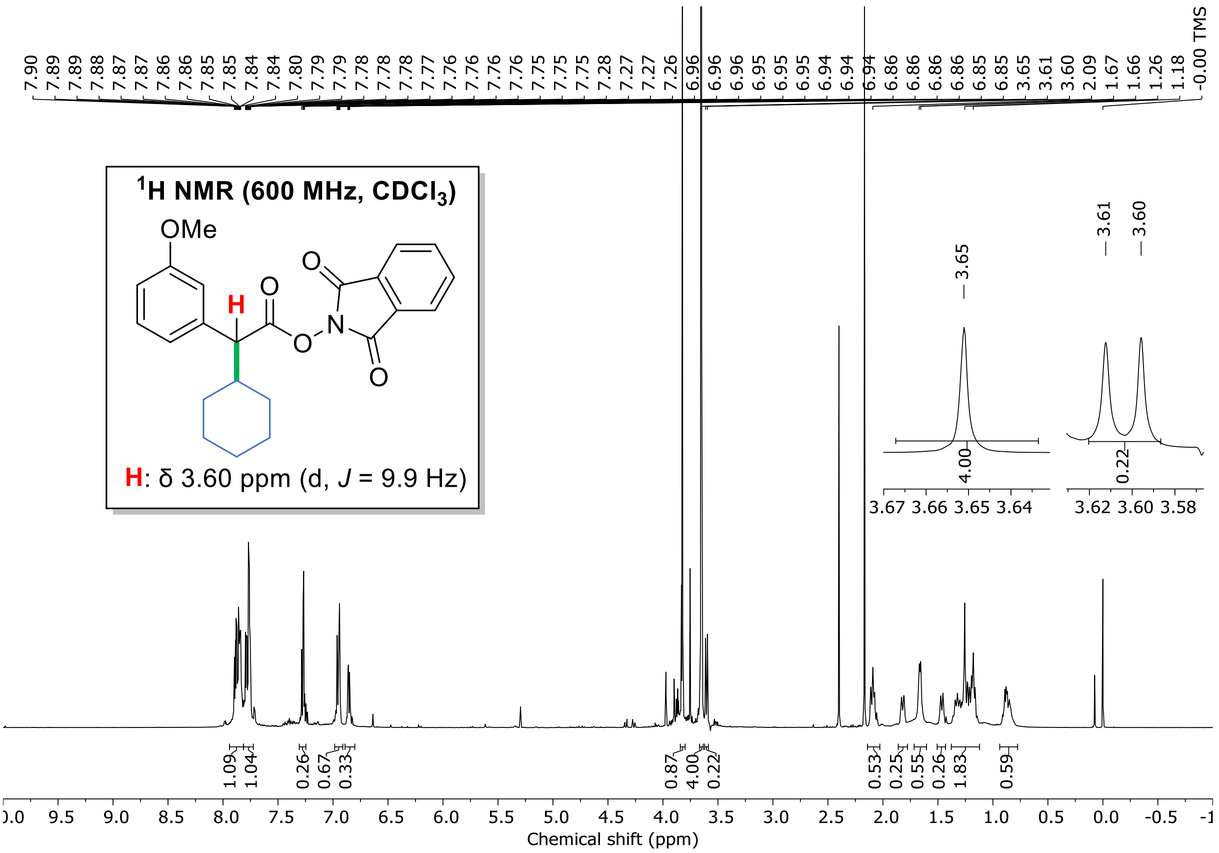

1,2-dibromoethane as internal standard (δ 3.65 ppm)

1,3-dioxoisoindolin-2-yl 2-cyclohexyl-2-(*p*-tolyl)acetate 5g

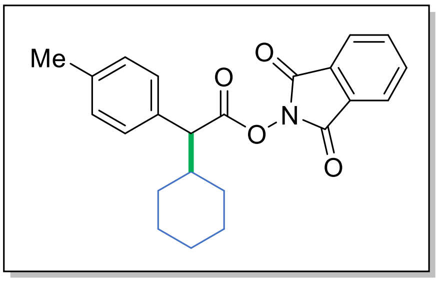

Synthesized according to the general procedure **GP3**. Product isolated as white solid (20 mg, 0.05 mmol, 53% yield) by flash chromatography (hexane -AcOEt mixtures, gradually from 0% to 30% of AcOEt in hexane).

m.p. 149 – 151 °C

^{1}H NMR (500 MHz, $CDCl_3$) δ 7.89–7.81 (m, 2H), 7.77 (dd, *J* = 5.5, 3.1 Hz, 2H), 7.30–7.23 (m, 2H), 7.18 (d, *J* = 7.8 Hz, 2H), 3.61 (d, *J* = 9.9 Hz, 1H), 2.36 (s, 3H), 2.16–2.02 (m, 2H), 1.83 (d, *J* = 11.4 Hz, 1H), 1.73–1.61 (m, 2H), 1.47 (d, *J* = 12.2 Hz, 1H), 1.41–1.12 (m, 4H), 0.95–0.80 (m, 1H) ppm.

^{13}C NMR (126 MHz, $CDCl_3$) δ 170.3, 162.1, 137.6, 134.8, 132.7, 129.6, 129.2, 128.7, 124.0, 55.3, 41.6, 31.8, 30.5, 26.4, 26.2, 26.1, 21.3 ppm.

HRMS (ESI) m/z: [M+Na]$^+$ calculated for $C_{23}H_{23}NNaO_4$ 400.1525; found 400.1527.

Methyl 4-(1-cyclohexyl-2-((1,3-dioxoisoindolin-2-yl)oxy)-2-oxoethyl)benzoate 5h

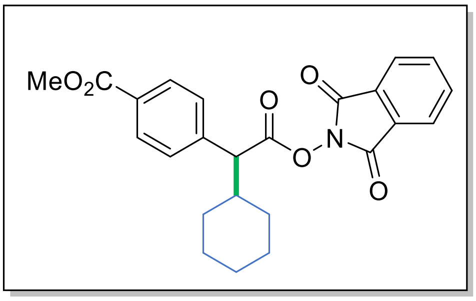

Synthesized according to the general procedure **GP3**. Product isolated as white solid (22 mg, 0.05 mmol, 52% yield) by flash chromatography (hexane -AcOEt mixtures, gradually from 0% to 30% of AcOEt in hexane).

m.p. 146 – 149 °C

^{1}H NMR (500 MHz, $CDCl_3$) δ 8.04 (d, *J* = 8.2 Hz, 2H), 7.95–7.81 (m, 2H), 7.81–7.66 (m, 2H), 7.47 (d, *J* = 8.3 Hz, 2H), 3.92 (s, 3H), 3.71 (d, *J* = 9.7 Hz, 1H), 2.19–2.05 (m, 2H), 1.88–1.77 (m, 1H), 1.75–1.60 (m, 2H), 1.44–1.28 (m, 2H), 1.28–1.11 (m, 3H), 0.96–0.82 (m, 1H) ppm.

^{13}C NMR (126 MHz, $CDCl_3$) δ 169.7, 166.9, 161.9, 140.8, 134.9, 130.1, 130.0, 129.1, 128.9, 124.1, 55.6, 52.3, 41.8, 31.7, 30.5, 26.2, 26.1, 26.0 ppm.

HRMS (ESI) m/z: $[M+Na]^+$ calculated for $C_{24}H_{23}NNaO_6$ 444.1423; found 444.1423.

C-H insertion reaction with n-pentane

A mixture of 3 C-H insertion products were formed in 38% yield (NMR) and **5j**:**5j'**:**5j''**= 4: 1: 1.4.
HRMS (ESI) m/z: $[M+Na]^+$ calculated for $C_{21}H_{21}NNaO_4$ 374.1368; found 374.1371.

^{1}H NMR (600 MHz, CDCl$_3$)

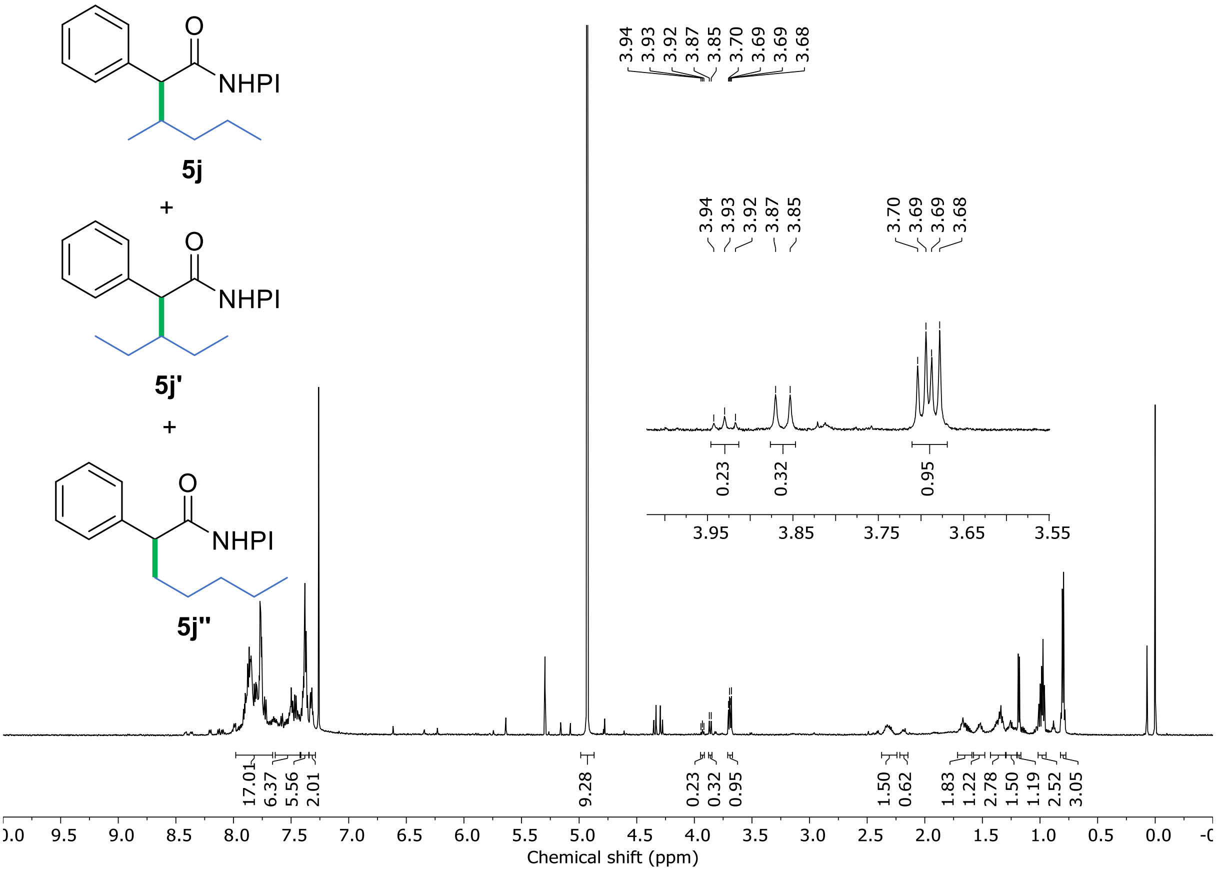

4.3. General procedure for 1,2-shift rearrangement (GP4)

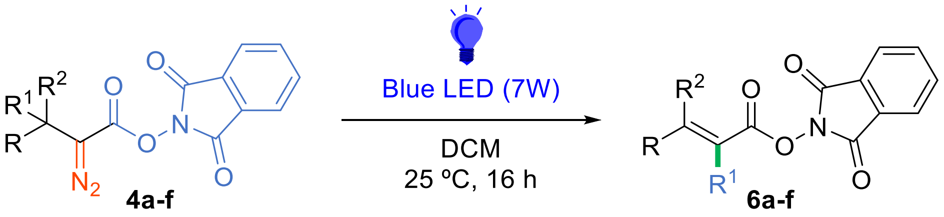

R, R^1 and R^2= H or C

A glass vial equipped with a stirring bar was charged with alkyl NHPI-diazoacetate (0.1 mmol), then sealed with an aluminum cap with a rubber septum. Dry DCM (1.0 mL) was added into the vial under the argon atmosphere, followed by the oxygen removal from the solution by freeze-pump-thaw technique. The reaction mixture was placed in a photoreactor and irradiated with blue LED (450 nm, 7 W) for 16 h. After that time, the crude reaction mixture was concentrated under vacuum and purified by column chromatography using ethyl hexanes/ethyl acetate to afford the final product.

1,3-dioxoisoindolin-2-yl acrylate 6a[8]

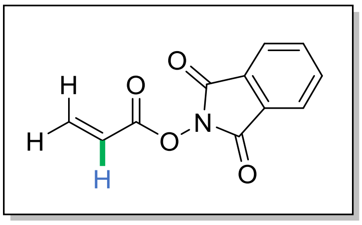

Synthesized according to the general procedure **GP4**. Product isolated as white solid (17 mg, 0.08 mmol, 79% yield) by flash chromatography (hexane -AcOEt mixtures, gradually from 0% to 30% of AcOEt in hexane).

^{1}H NMR (500 MHz, $CDCl_3$) δ 7.90 (dd, *J* = 5.5, 3.1 Hz, 2H), 7.80 (dd, *J* = 5.5, 3.1 Hz, 2H), 6.74 (d, *J* = 17.4 Hz, 1H), 6.39 (dd, *J* = 17.4, 10.7 Hz, 1H), 6.19 (d, *J* = 10.7 Hz, 1H) ppm.

^{13}C NMR (126 MHz, $CDCl_3$) δ 162.2, 162.0, 136.3, 134.9, 129.1, 124.1, 123.2 ppm.

1,3-dioxoisoindolin-2-yl but-2-enoate 6b

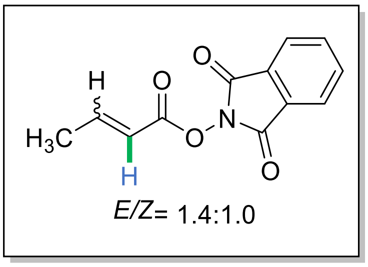

Synthesized according to the general procedure **GP4**. Product isolated as white solid (21 mg, 0.09 mmol, 89% yield) by flash chromatography (hexane -AcOEt mixtures, gradually from 0% to 30% of AcOEt in hexane).

m.p. 116 – 120 °C

^{1}H NMR (500 MHz, CDCl3) [mixture of *E*/*Z* isomers] δ 7.92–7.86 (m, 4H), 7.81–7.76 (m, 4H), 7.33 (dq, *J* = 15.3, 6.9 Hz, 1H), 6.74 (dq, *J* = 11.3, 7.3 Hz, 1H), 6.17–6.06 (m, 2H), 2.22 (dd, *J* = 7.3, 1.8 Hz, 3H), 2.02 (dd, *J* = 7.0, 1.7 Hz, 3H) ppm.

^{13}C NMR (126 MHz, CDCl3) [mixture of *E/Z* isomers] δ 162.3 (2C), 162.2, 161.8, 152.5, 151.3, 134.8, 129.2 (2C), 124.1, 117.3, 114.9, 18.9, 16.3 ppm.

HRMS (ESI) m/z: [M+Na]$^{+}$ calculated for $C_{12}H_{9}NNaO_{4}$ 254.0429; found 254.0432.

benzyl (E/Z)-but-2-enoate 6b'*

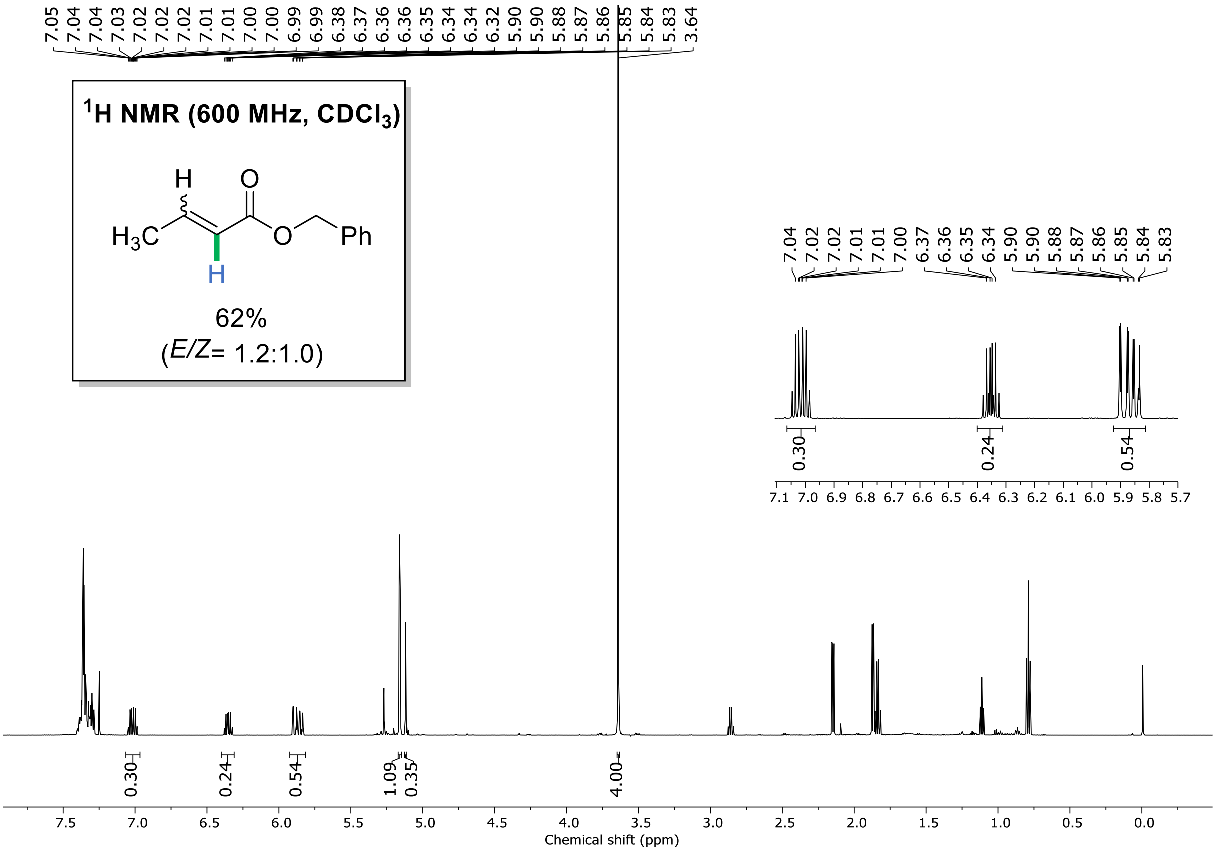

1,2-dibromoethane as internal standard (δ 3.65 ppm).

1,3-dioxoisoindolin-2-yl 3-methylbut-2-enoate 6c

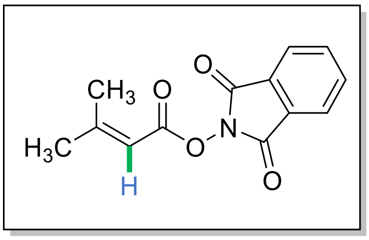

Synthesized according to the general procedure **GP4**. Product isolated as white solid (19 mg, 0.08 mmol, 78% yield) by flash chromatography (hexane -AcOEt mixtures, gradually from 0% to 30% of AcOEt in hexane).

m.p. 112 – 113 °C

^{1}H NMR (500 MHz, $CDCl_3$) δ 7.88 (dd, *J* = 5.5, 3.1 Hz, 2H), 7.78 (dd, *J* = 5.5, 3.1 Hz, 2H), 5.99 (s, 1H), 2.23 (s, 3H), 2.04 (s, 3H) ppm.

^{13}C NMR (126 MHz, $CDCl_3$) δ 165.6, 162.5, 162.0, 134.8, 129.2, 124.0, 110.2, 28.1, 21.2 ppm.

HRMS (ESI) m/z: $[M+Na+MeOH]^+$ calculated for $C_{14}H_{15}NNaO_5$ 300.0848; found 300.0852.

1,3-dioxoisoindolin-2-yl 2-cyclohexylideneacetate 6d

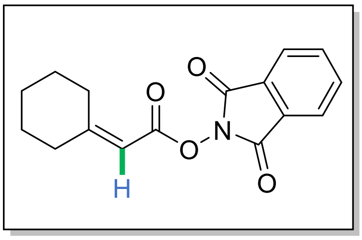

Synthesized according to the general procedure **GP4**. Product isolated as white solid (22 mg, 0.08 mmol, 76% yield) by flash chromatography (hexane -AcOEt mixtures, gradually from 0% to 30% of AcOEt in hexane).

m.p. 127 – 130 °C

^{1}H NMR (500 MHz, $CDCl_3$) δ 7.88 (dd, *J* = 5.4, 3.1 Hz, 2H), 7.77 (dd, *J* = 5.4, 3.1 Hz, 2H), 5.91 (s, 1H), 2.83 (t, *J* = 6.1 Hz, 2H), 2.32 (t, *J* = 6.2 Hz, 2H), 1.77–1.69 (m, 2H), 1.68–1.59 (m, 4H) ppm.

^{13}C NMR (126 MHz, $CDCl_3$) δ 172.6, 162.6, 162.0, 134.8, 129.2, 124.0, 107.0, 38.6, 30.8, 28.7, 27.9, 26.1 ppm.

HRMS (ESI) m/z: $[M+Na]^+$ calculated for $C_{16}H_{15}NNaO_4$ 308.0899; found 308.0903.

Benzyl 2-cyclohexylideneacetate 6d'*

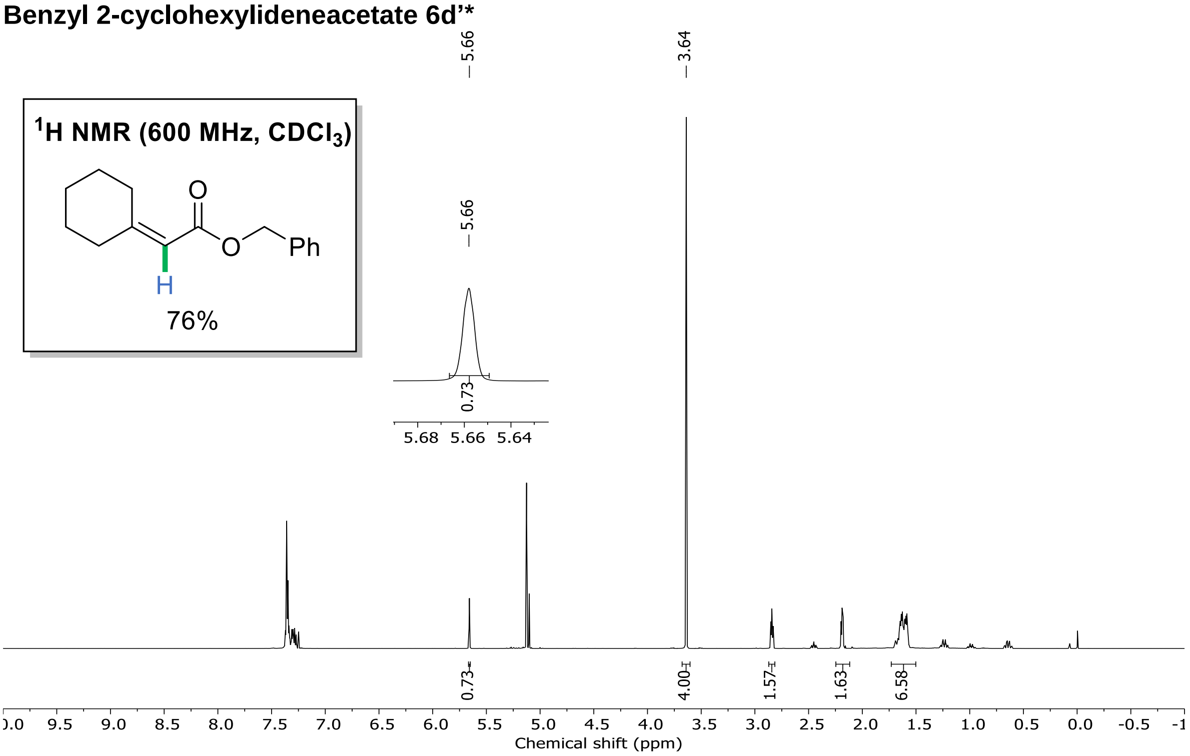

1,2-dibromoethane as internal standard (δ 3.65 ppm).

1,3-dioxoisoindolin-2-yl 2,3-dimethylbut-2-enoate 6e

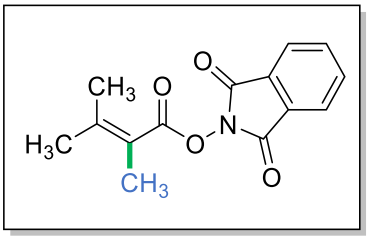

Synthesized according to the general procedure **GP4**. Product isolated as white solid (19 mg, 0.07 mmol, 68% yield) by flash chromatography (hexane -AcOEt mixtures, gradually from 0% to 30% of AcOEt in hexane).

m.p. 98 – 101 °C

^{1}H NMR (500 MHz, $CDCl_3$) δ 7.89 (dd, J = 5.5, 3.1 Hz, 2H), 7.78 (dd, J = 5.5, 3.1 Hz, 2H), 2.19–2.14 (m, 3H), 2.10–2.05 (m, 3H), 1.95 (s, 3H) ppm.

^{13}C NMR (126 MHz, $CDCl_3$) δ 164.6, 162.6, 152.9, 134.8, 129.3, 124.0, 117.8, 23.9, 23.5, 15.5 ppm.

HRMS (ESI) m/z: $[M+Na]^+$ calculated for $C_{14}H_{13}NNaO_4$ 282.0742; found 282.0744.

1,3-dioxoisoindolin-2-yl 2,3-dimethylpent-2-enoate (6fa) and 1,3-dioxoisoindolin-2-yl 2-ethyl-3-methylbut-2-enoate (6fb)

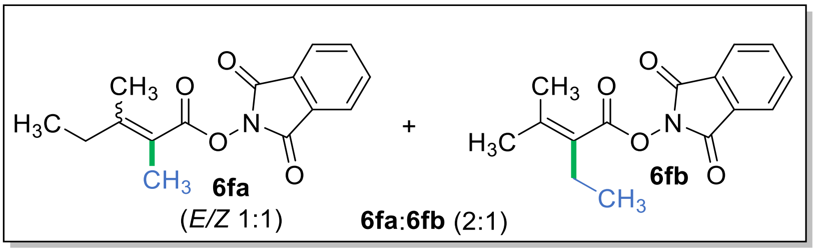

Synthesized according to the general procedure **GP4**. Product isolated as white solid (21 mg, 0.08 mmol, 73% yield) by flash chromatography (hexane -AcOEt mixtures, gradually from 0% to 30% of AcOEt in hexane).

m.p. 71 – 75 °C

^{1}H NMR (500 MHz, $CDCl_3$) [Mixture of isomers] δ 7.92–7.83 (m, 6H), 7.81–7.74 (m, 6H), 2.50 (q, J = 7.5 Hz, 4H), 2.27 (q, J = 7.6 Hz, 2H), 2.16–2.10 (m, 5H), 2.07 (s, 2H), 2.05 (s, 3H), 1.93 (s, 2H), 1.91 (s, 3H), 1.31–1.20 (m, 3H), 1.17 (m, 3H), 1.08 (t, J = 7.6 Hz, 6H [overlapped]) ppm.

^{13}C NMR (151 MHz, $CDCl_3$) δ 165.1, 164.8, 164.5, 162.6, 162.5, 157.9, 157.4, 150.1, 134.8, 129.3, 124.0, 117.3, 117.2, 30.3, 29.9, 23.8, 23.3, 22.3, 21.5, 20.7, 15.6, 14.8, 13.5, 12.8, 11.6 ppm.

HRMS (ESI) m/z: $[M+Na+MeOH]^+$ calculated for $C_{16}H_{19}NNaO_5$ 328.1161; found 328.1165.

General procedure for O-H insertion (GP5)

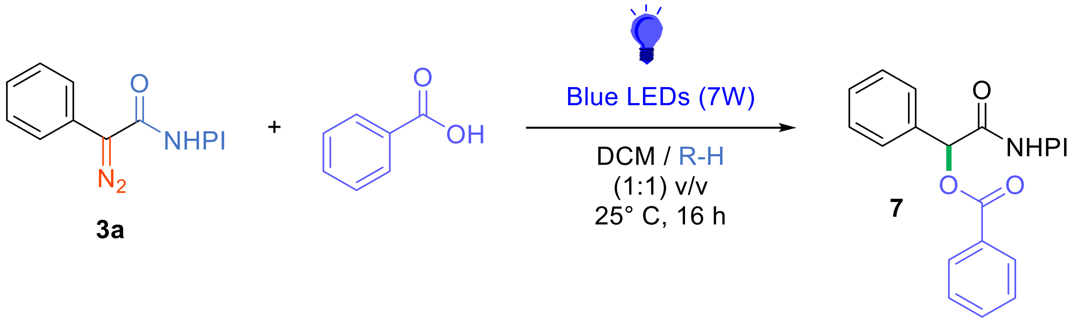

A glass vial equipped with a stirring bar was charged with NHPI diazoacetate **3a** (31 mg, 0.1 mmol) and benzoic acid (24.4 mg, 0.2 mmol, 2.0 equiv) then sealed with an aluminum cap with a rubber septum. Dry DCM (1.0 mL) was added into the vial under the argon atmosphere, followed by the oxygen removal from the solution by freeze-pump-thaw technique. The reaction vial was placed in a photoreactor and was irradiated with blue LED (450 nm, 7 W) for 16 h. After that time, the crude reaction mixture was concentrated under vacuum and purified by column chromatography (silica gel) using hexanes/ethyl acetate to afford the final product.

2-((1,3-dioxoisoindolin-2-yl)oxy)-2-oxo-1-phenylethyl benzoate 7

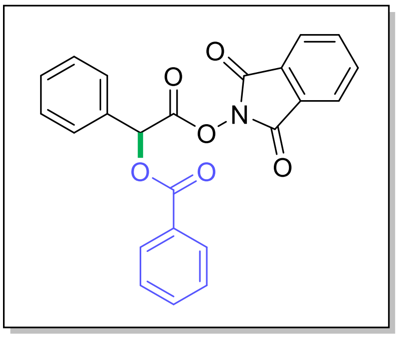

Synthesized according to the general procedure GP4. Product isolated as viscous oil (30.0 mg, 0.08 mmol, 75% yield) by flash chromatography (hexane -AcOEt mixtures, gradually from 0% to 30% of AcOEt in hexane).

^{1}H NMR (500 MHz, $CDCl_3$) δ 8.18 – 8.13 (m, 2H), 7.89 – 7.83 (m, 2H), 7.77 (dd, *J* = 5.5, 3.1 Hz, 2H), 7.74 – 7.68 (m, 2H), 7.62 – 7.57 (m, 1H), 7.52 – 7.43 (m, 5H), 6.64 (s, 1H) ppm.

^{13}C NMR (126 MHz, $CDCl_3$) δ 165.82, 165.46, 161.43, 134.98, 134.47, 133.87, 132.54, 130.27, 130.20, 129.28, 128.64, 128.32, 124.17, 73.06 ppm.

HRMS (ESI) m/z: $[M+Na]^+$ calculated for $C_{23}H_{15}NNaO_6$ 424.0797; found 424.0800.

5. REFERENCES

(1) Yu, Z.; Mendoza, A. Enantioselective Assembly of Congested Cyclopropanes Using Redox-Active Aryldiazoacetates. *ACS Catal.* **2019**, *9* (9), 7870–7875. https://doi.org/10.1021/acscatal.9b02615.

(2) Montesinos-Magraner, M.; Costantini, M.; Ramírez-Contreras, R.; Muratore, M. E.; Johansson, M. J.; Mendoza, A. General Cyclopropane Assembly by Enantioselective Transfer of a Redox-Active Carbene to Aliphatic Olefins. *Angew. Chemie Int. Ed.* **2019**, *58* (18), 5930–5935. https://doi.org/10.1002/anie.201814123.

(3) Hashimoto, T.; Miyamoto, H.; Naganawa, Y.; Maruoka, K. Stereoselective Synthesis of α-Alkyl-β-Keto Imides via Asymmetric Redox C−C Bond Formation between α-Alkyl-α-Diazocarbonyl Compounds and Aldehydes. *J. Am. Chem. Soc.* **2009**, *131* (32), 11280–11281. https://doi.org/10.1021/ja903500w.

(4) Modak, A.; Alegre-Requena, J. V.; de Lescure, L.; Rynders, K. J.; Paton, R. S.; Race, N. J. Homologation of Electron-Rich Benzyl Bromide Derivatives via Diazo C–C Bond Insertion. *J. Am. Chem. Soc.* **2022**, *144* (1), 86–92. https://doi.org/10.1021/jacs.1c11503.

(5) Wang, D.; Zhu, N.; Chen, P.; Lin, Z.; Liu, G. Enantioselective Decarboxylative Cyanation Employing Cooperative Photoredox Catalysis and Copper Catalysis. *J. Am. Chem. Soc.* **2017**, *139* (44), 15632–15635. https://doi.org/10.1021/jacs.7b09802.

(6) Chen, K.-Q.; Shen, J.; Wang, Z.-X.; Chen, X.-Y. A Donor–Acceptor Complex Enables the Synthesis of E -Olefins from Alcohols, Amines and Carboxylic Acids. *Chem. Sci.* **2021**, *12* (19), 6684–6690. https://doi.org/10.1039/D1SC01024G.

(7) Matuszewski, B. Photolysis of ethyl α-chlorophenylacetate in cyclohexane**.** *Journal of Photochemistry, 7,* ***1977****, 1-15*

(8) Schnaar, R. L.; Lee, Y. C. Polyacrylamide Gels Copolymerized with Active Esters. New Medium for Affinity Systems. *Biochemistry* **1975**, *14* (7), 1535–1541. https://doi.org/10.1021/bi00678a030.

6. UV/VIS SPECTRA

1,3-dioxoisoindolin-2-yl 2-diazo-2-phenylacetate 3a
λ max= 421 nm (15.0 mM in DCM)

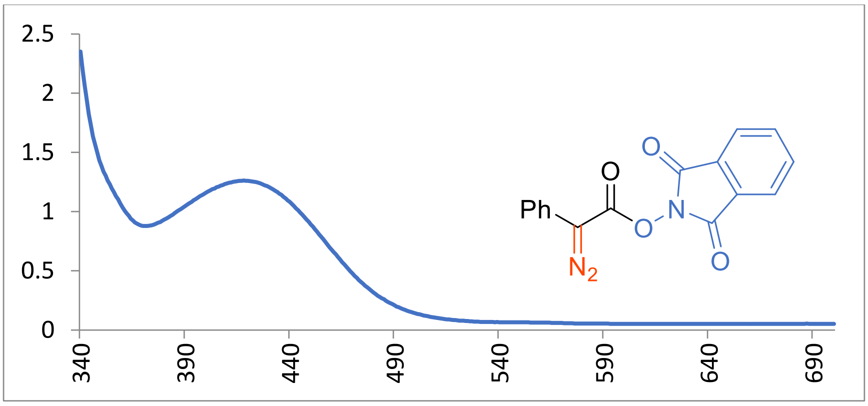

1,3-dioxoisoindolin-2-yl 2-diazopropanoate 4a
λ max= 356 nm (15.0 mM in DCM)

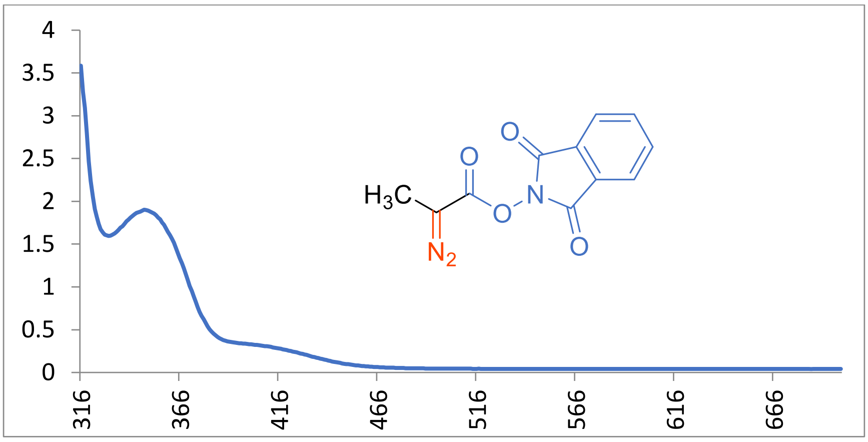

1,3-dioxoisoindolin-2-yl 2-diazobutanoate 4b
λ max= 396 nm (15.0 mM in DCM)

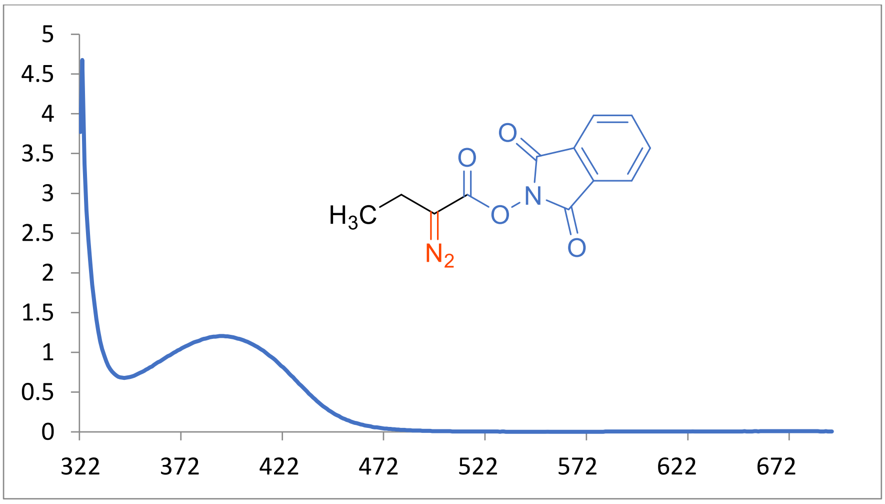

1,3-dioxoisoindolin-2-yl 2-diazo-3-methylbutanoate 4c

λ max= 397 nm (15.0 mM in DCM)

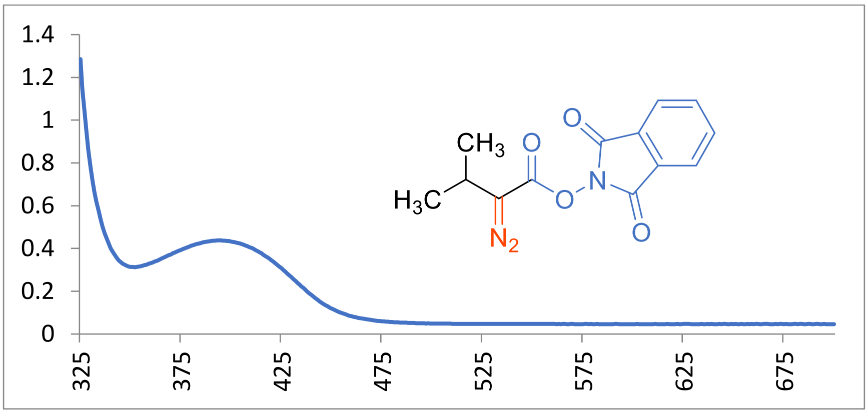

1,3-dioxoisoindolin-2-yl 2-cyclohexyl-2-diazoacetate 4d
λ max= 397 nm (15.0 mM in DCM)

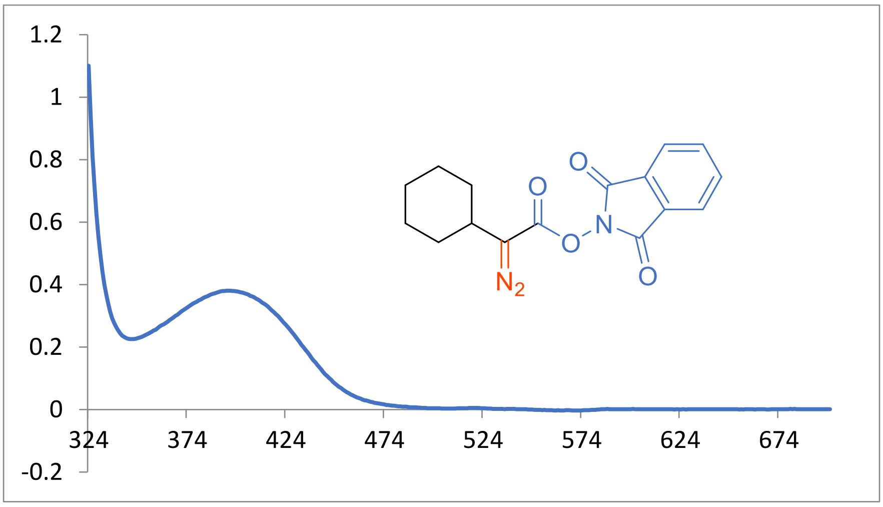

1,3-dioxoisoindolin-2-yl 2-diazo-3,3-dimethylbutanoate 4e
λ max= 393 nm (15.0 mM in DCM)

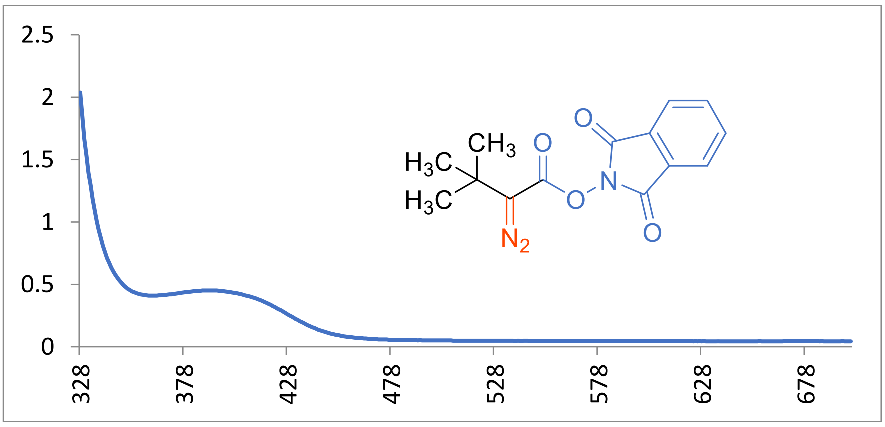

1,3-dioxoisoindolin-2-yl 2-diazo-3,3-dimethylpentanoate 4f

λ max= 392 nm (15.0 mM in DCM)

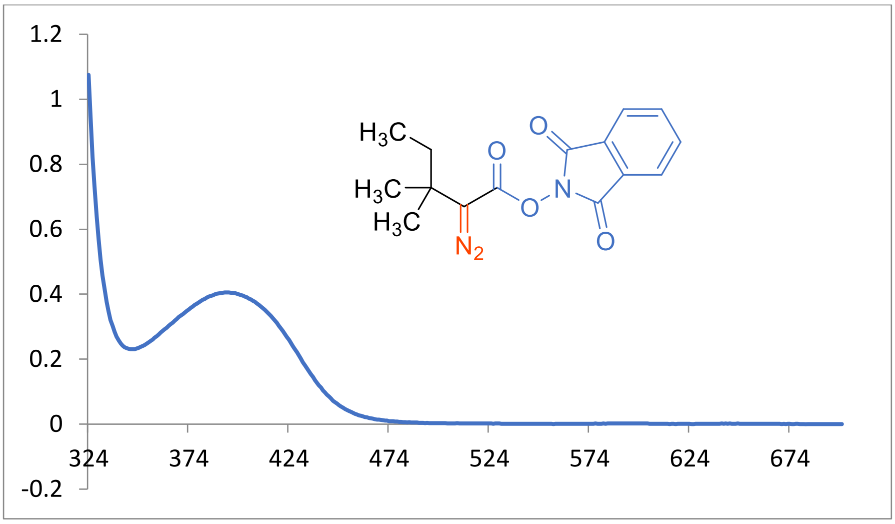

1,3-dioxoisoindolin-2-yl 2-diazo-2-(4-methoxyphenyl)acetate 3c

λ max= 422 nm (15.0 mM in DCM)

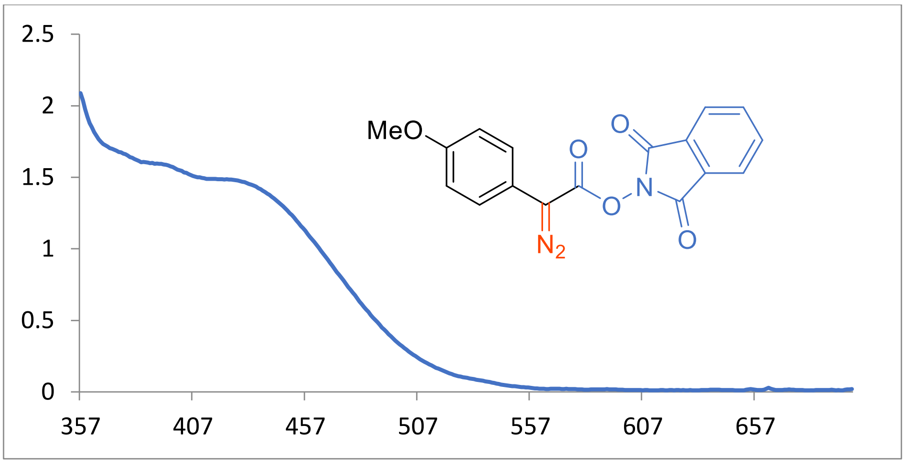

1,3-dioxoisoindolin-2-yl 2-diazo-2-(p-tolyl)acetate 3d

λ max= 424 nm (15.0 mM in DCM)

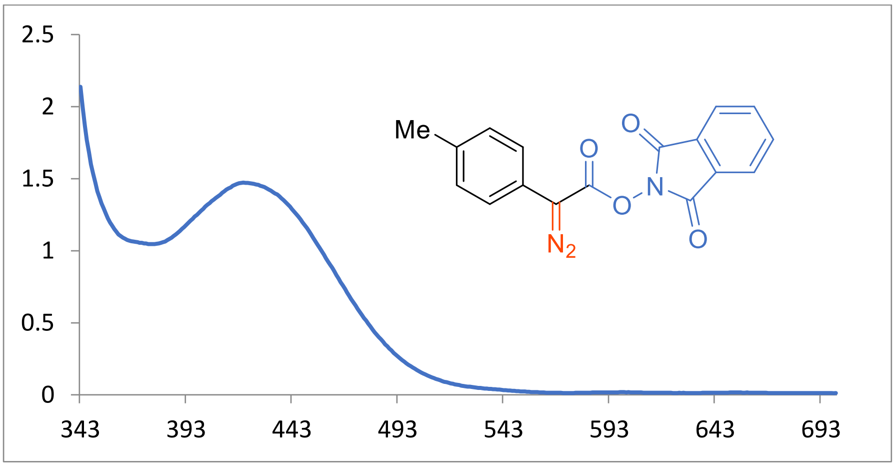

methyl 4-(1-diazo-2-((1,3-dioxoisoindolin-2-yl)oxy)-2-oxoethyl)benzoate 3e

λ max= 412 nm (15.0 mM in DCM)

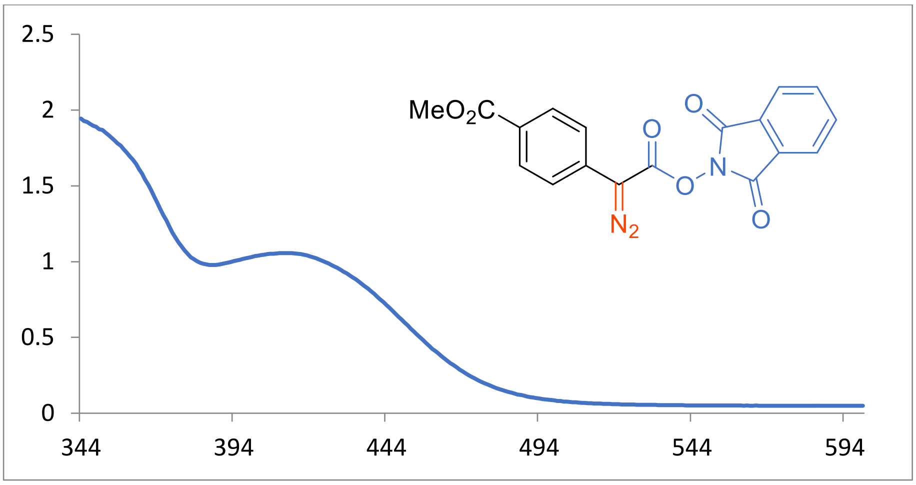

benzyl 2-cyclohexyl-2-diazoacetate 4d’

λ max= 415nm (15.0 mM in DCM)

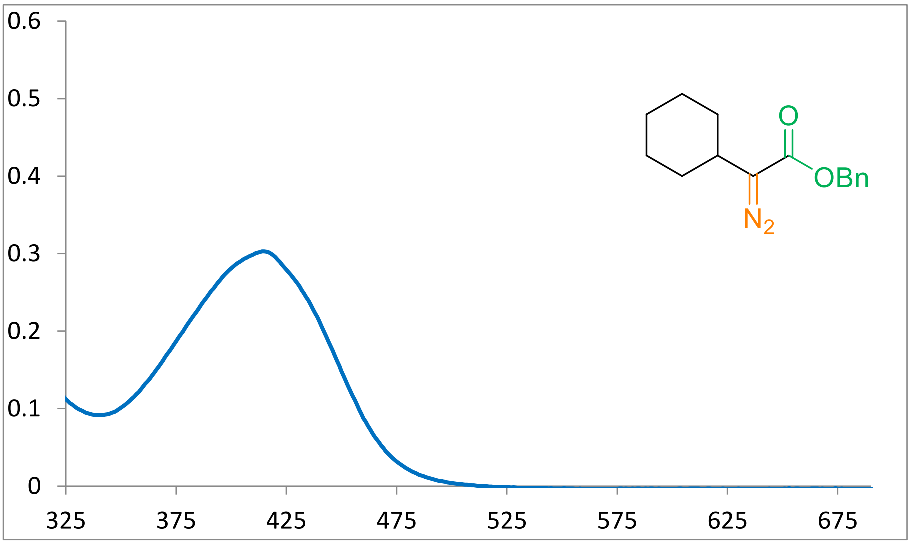

benzyl 2-diazobutanoate 4b’

λ max= 410nm (15.0 mM in DCM)

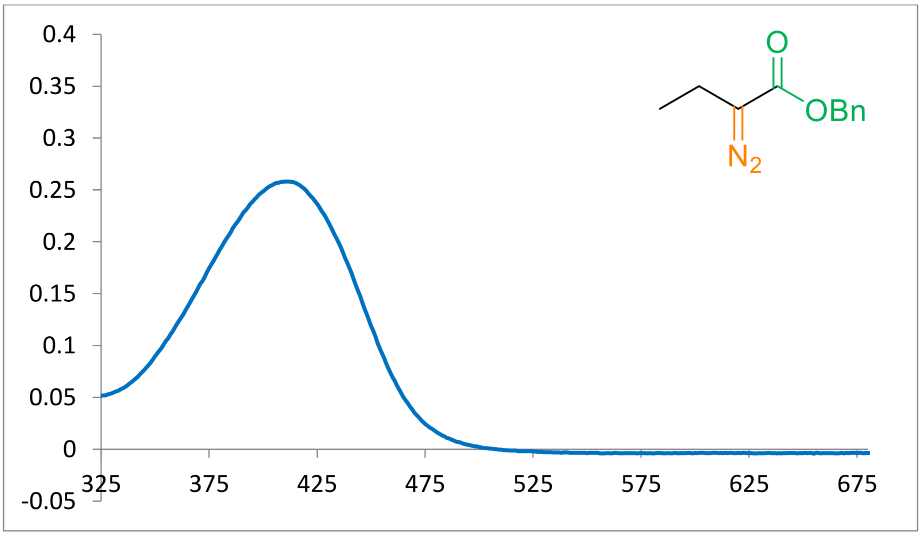

1,3-dioxoisoindolin-2-yl 2-diazo-2-(3-methoxyphenyl)acetate 3g
λ max= 416 nm (15.0 mM in DCM)

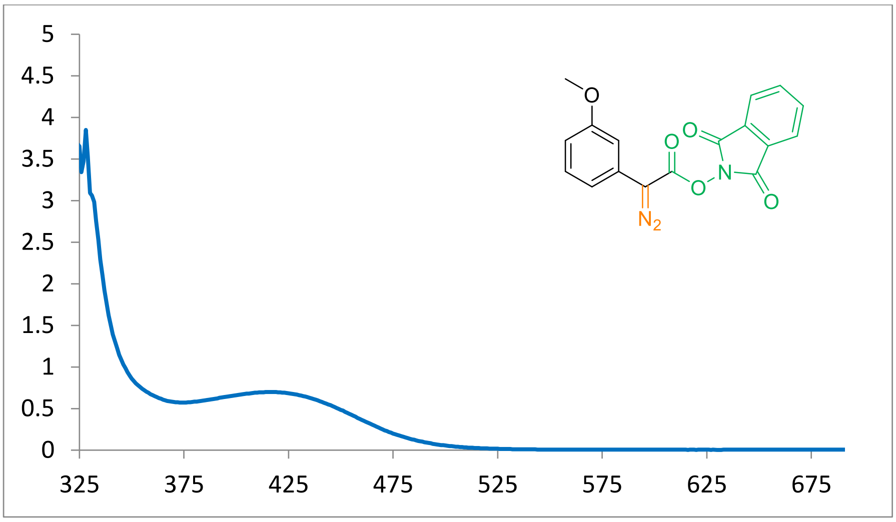

Combined UV/VIS spectra of NHPI diazoacetates

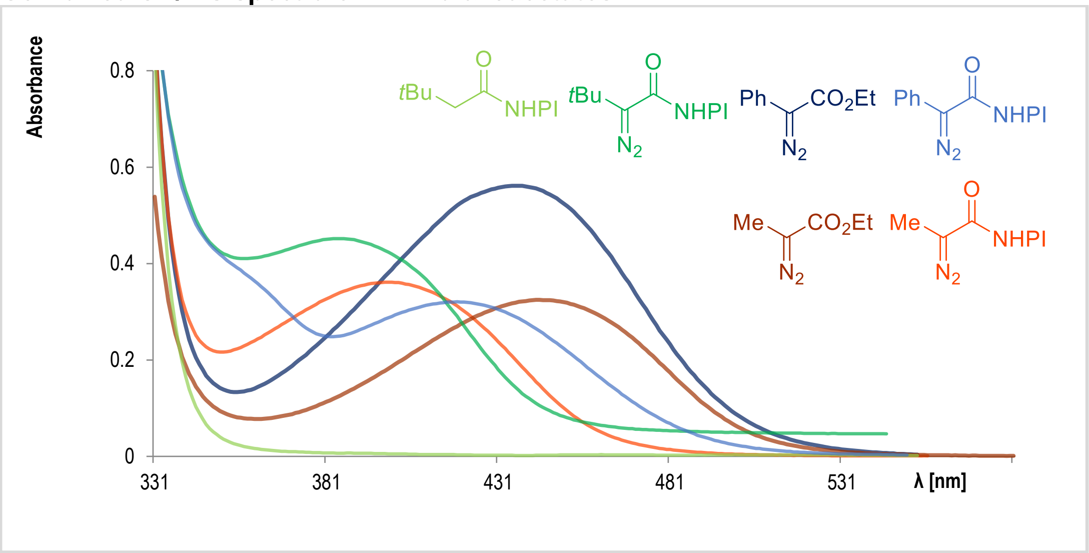

Concentration dependend UV/Vis spectra of **3a**

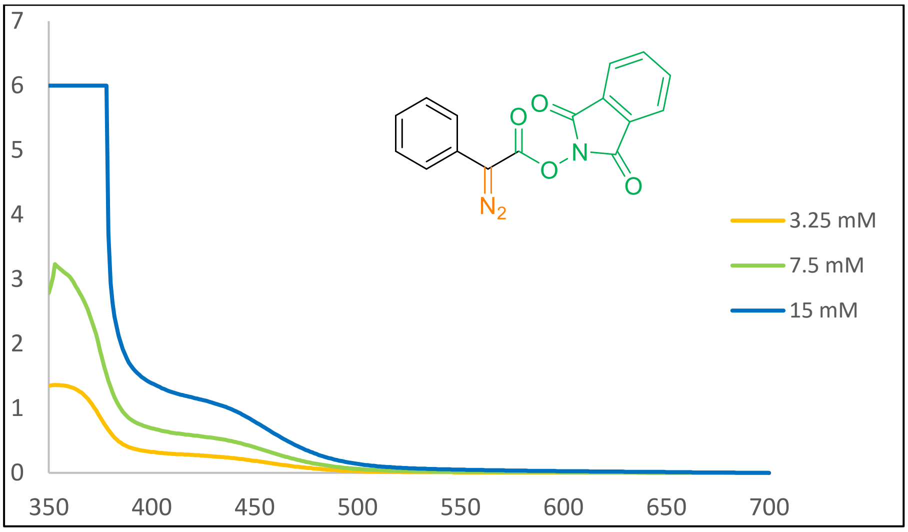

For concentration 0.1 M detector overload occurred, the measurement was repeated in cuvette d = 1.0 mm:

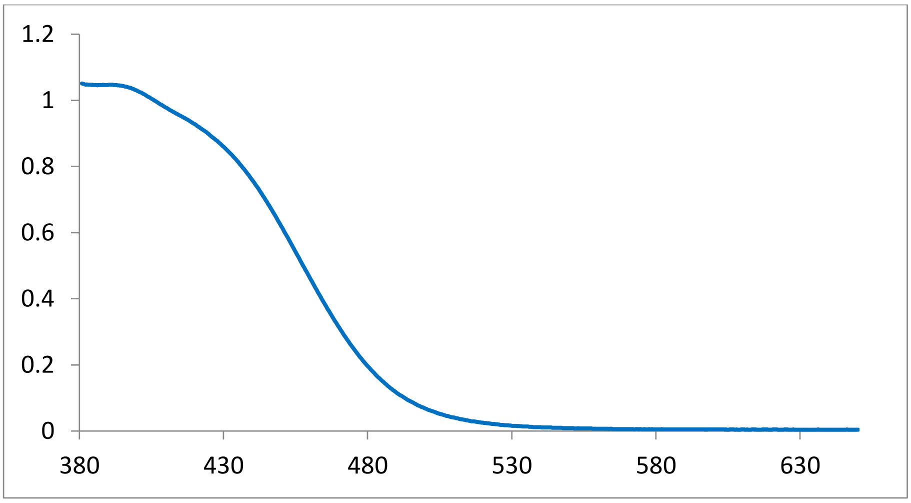

Concentration dependend UV/Vis spectra of **3a**

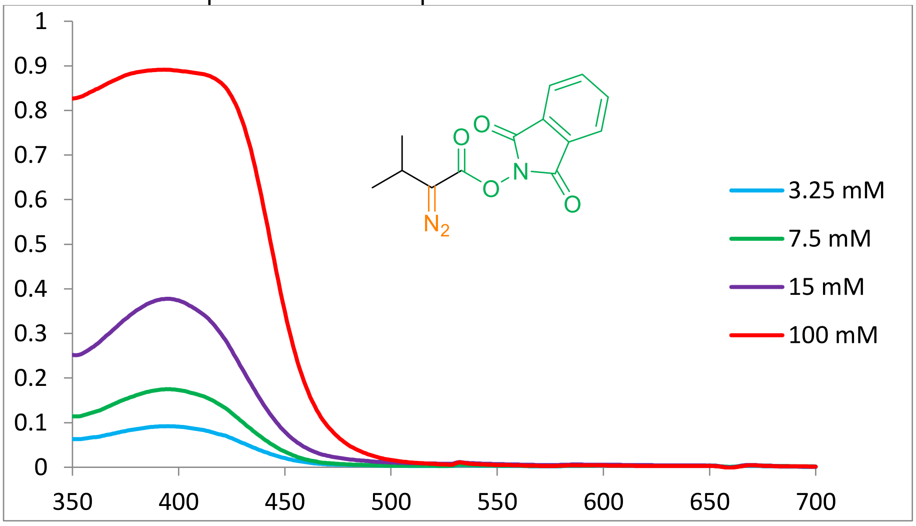

7. ^{1}H and ^{13}C NMR DATA

1,3-dioxoisoindolin-2-yl 2-diazo-2-phenylacetate 3a

^{1}H NMR (500 MHz, $CDCl_3$)

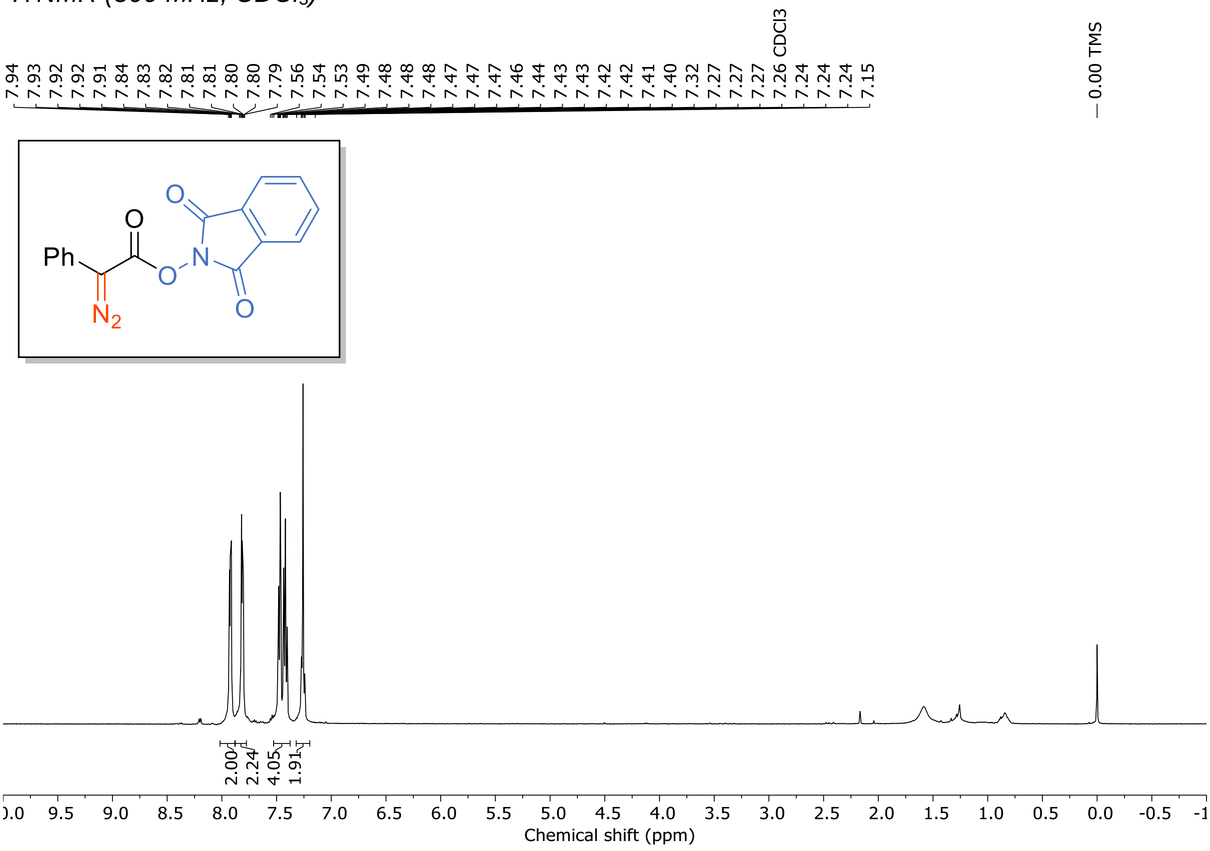

^{13}C NMR (126 MHz, $CDCl_3$)

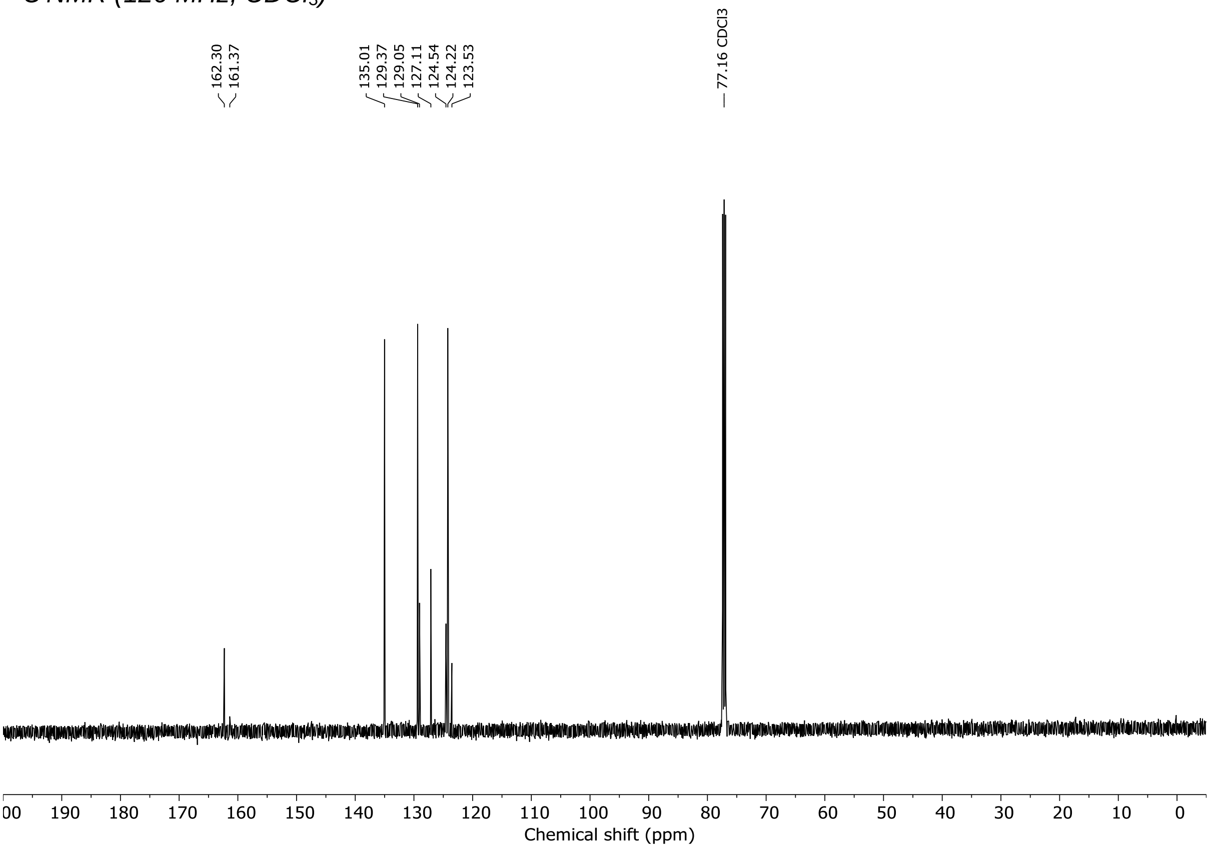

1,3-dioxoisoindolin-2-yl 2-diazoacetate 3b

^{1}H NMR (600 MHz, $CDCl_3$)

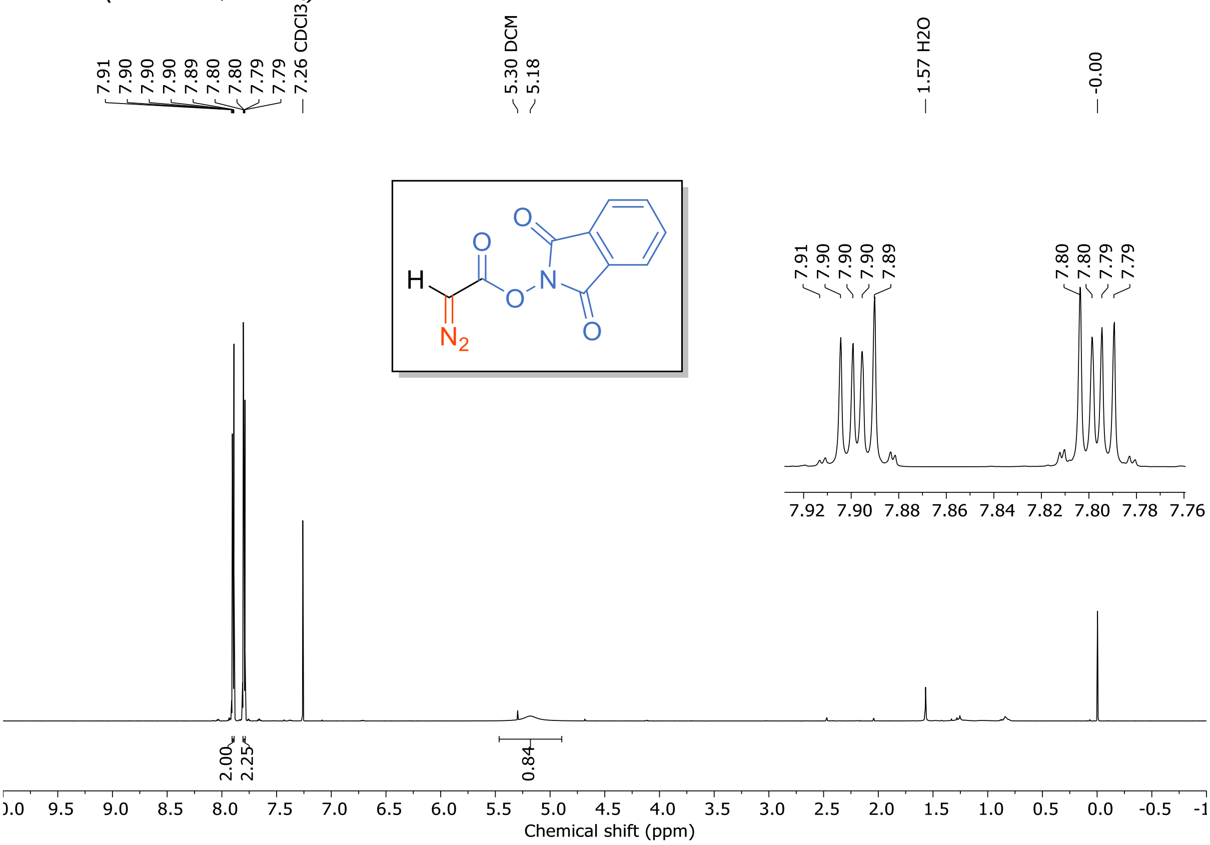

^{13}C NMR (151 MHz, $CDCl_3$)

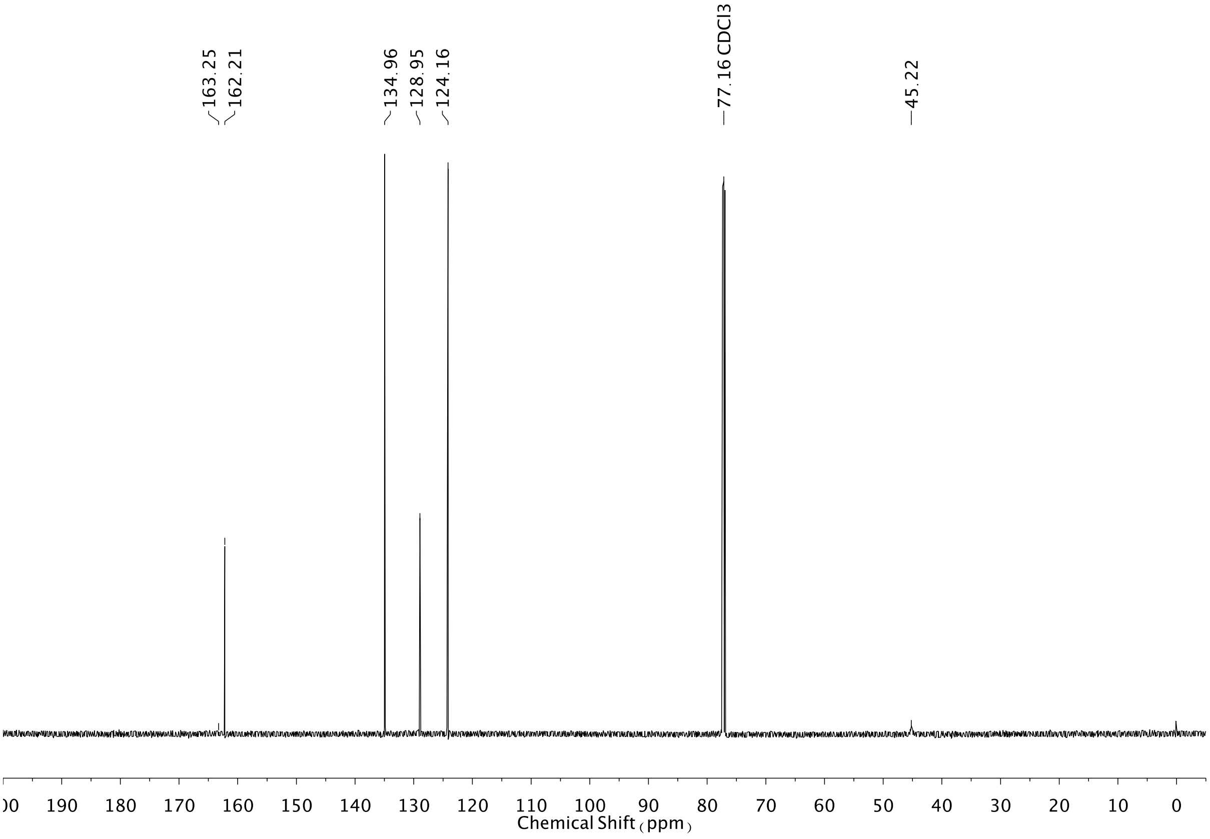

1,3-dioxoisoindolin-2-yl 2-diazopropanoate 4a

^{1}H NMR (500 MHz, $CDCl_3$)

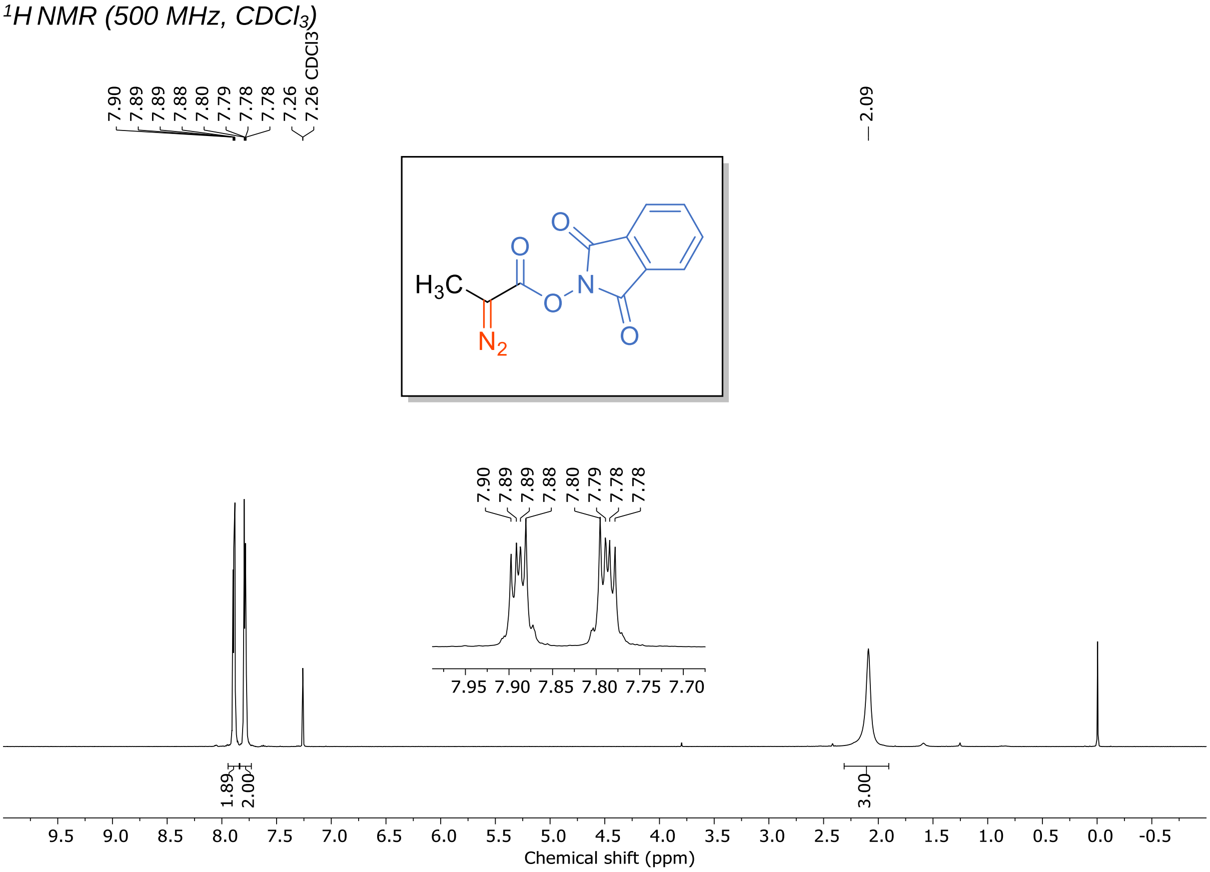

^{13}C NMR (126 MHz, $CDCl_3$)

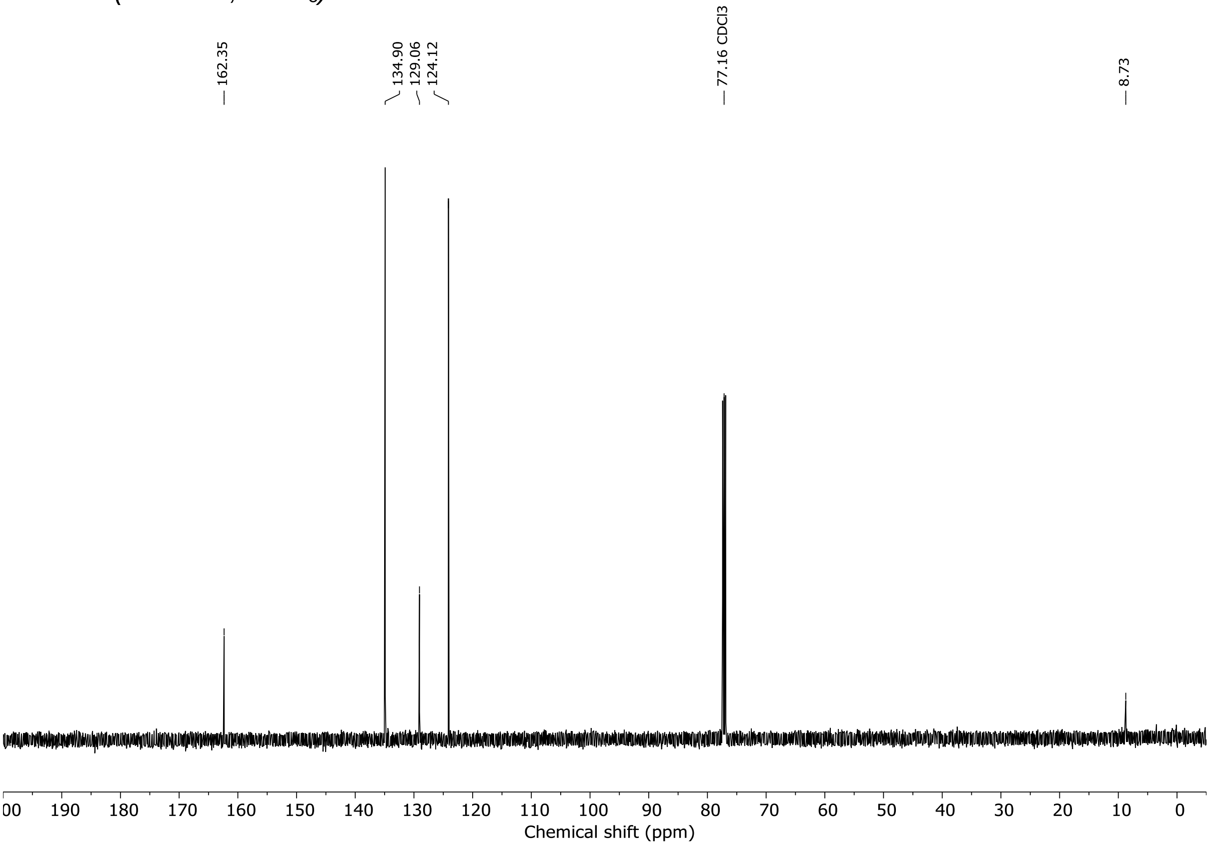

1,3-dioxoisoindolin-2-yl 2-diazobutanoate 4b

^{1}H NMR (500 MHz, $CDCl_3$)

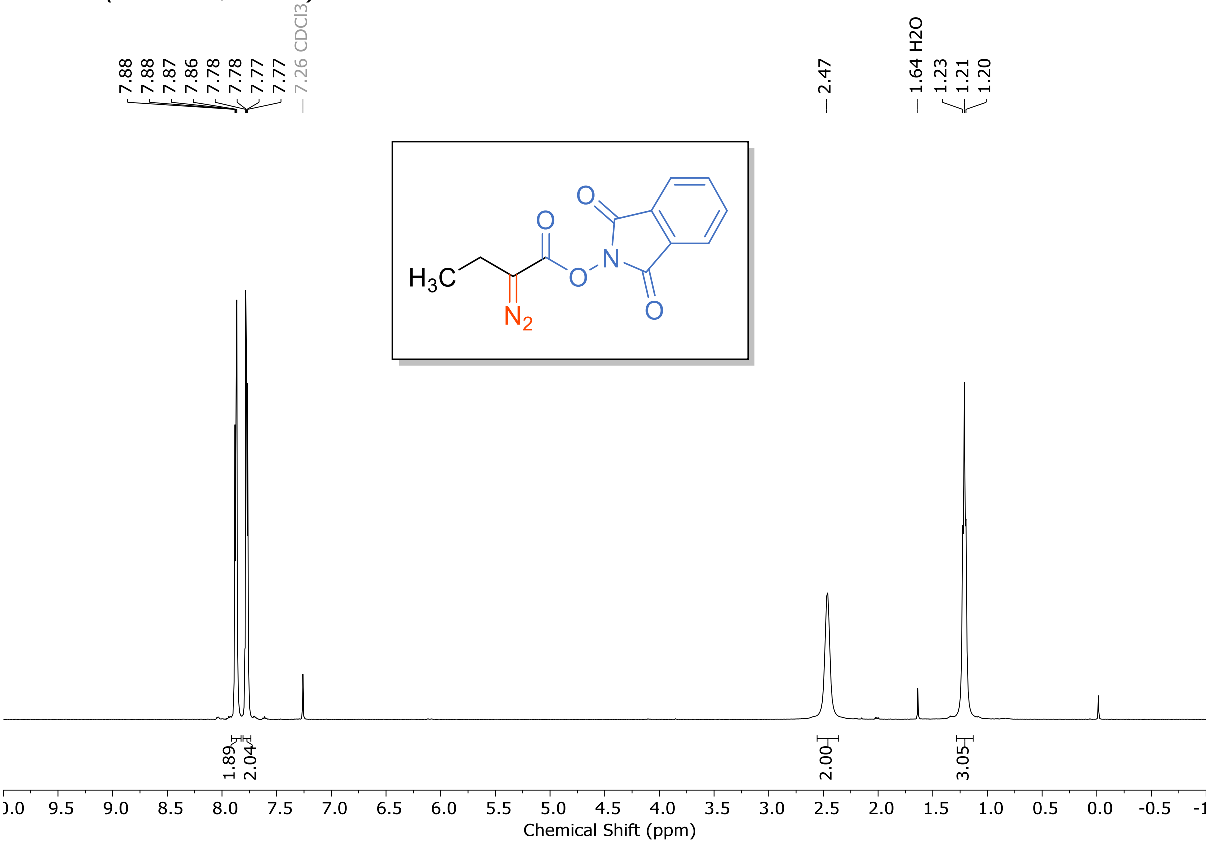

^{13}C NMR (126 MHz, $CDCl_3$)

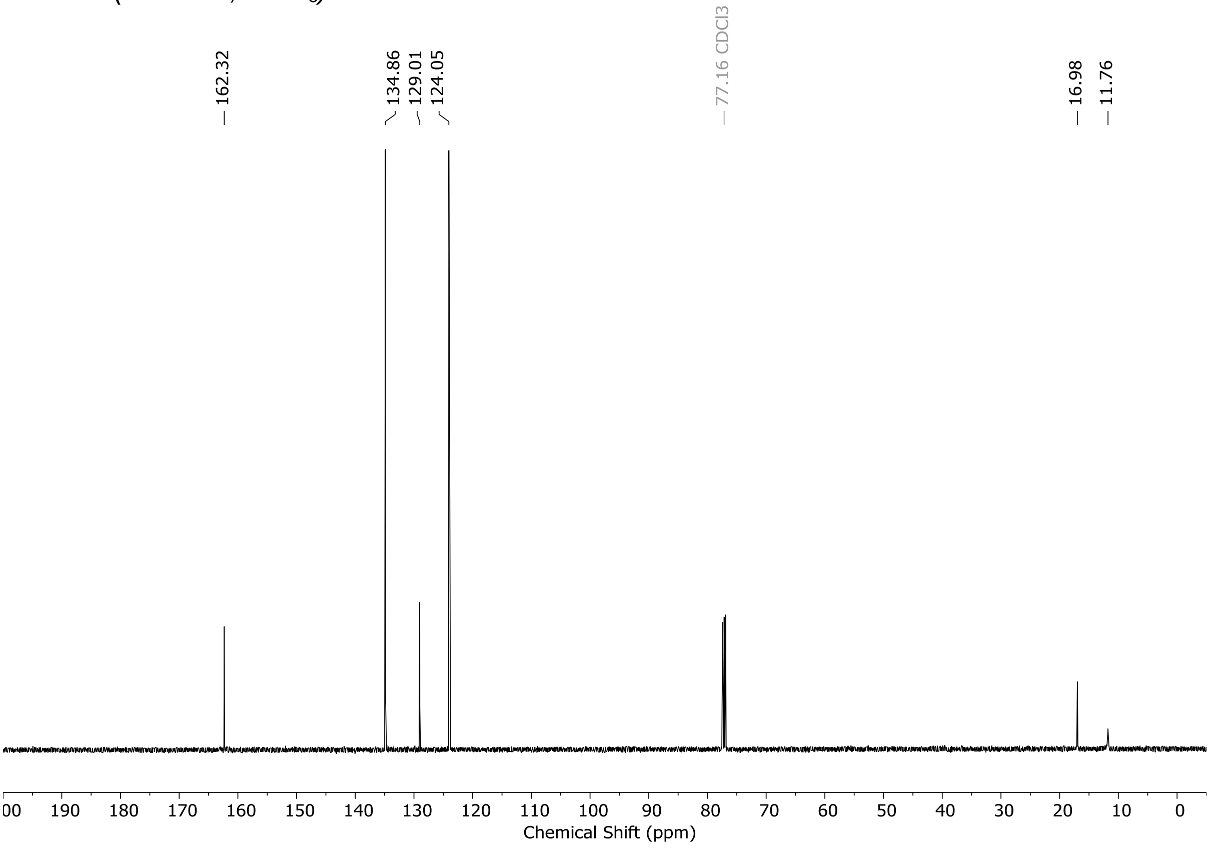

1,3-dioxoisoindolin-2-yl 2-diazo-3-methylbutanoate 4c

^{1}H NMR (500 MHz, $CDCl_3$)

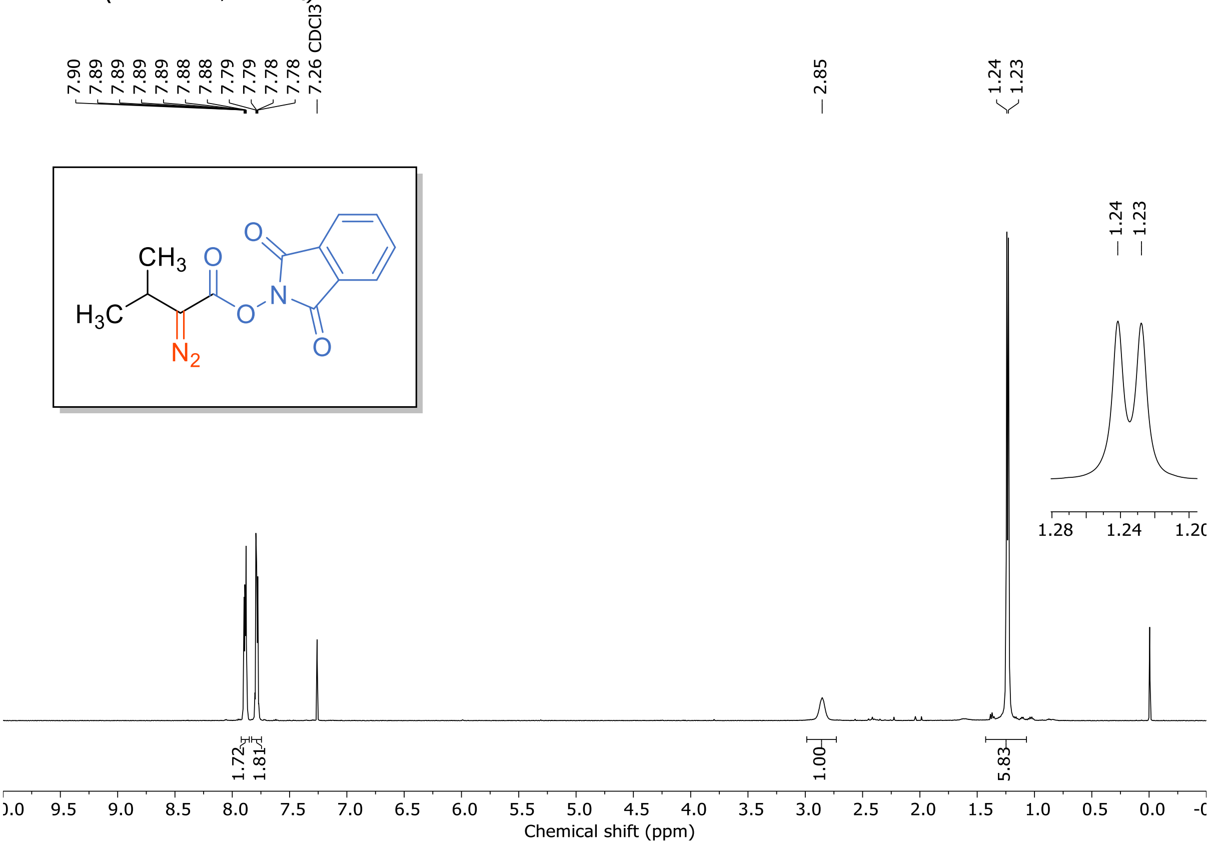

^{13}C NMR (126 MHz, $CDCl_3$)

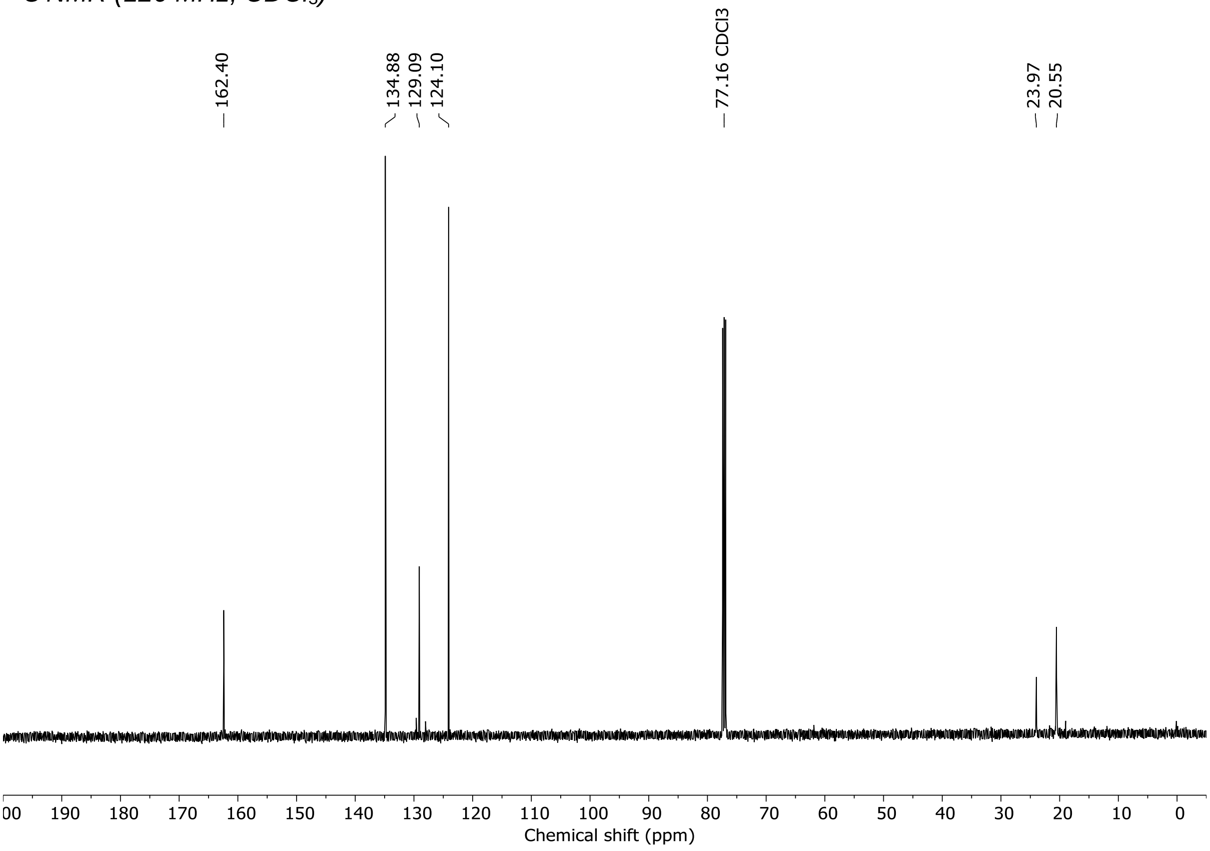

1,3-dioxoisoindolin-2-yl 2-cyclohexyl-2-diazoacetate 4d

^{1}H NMR (500 MHz, $CDCl_3$)

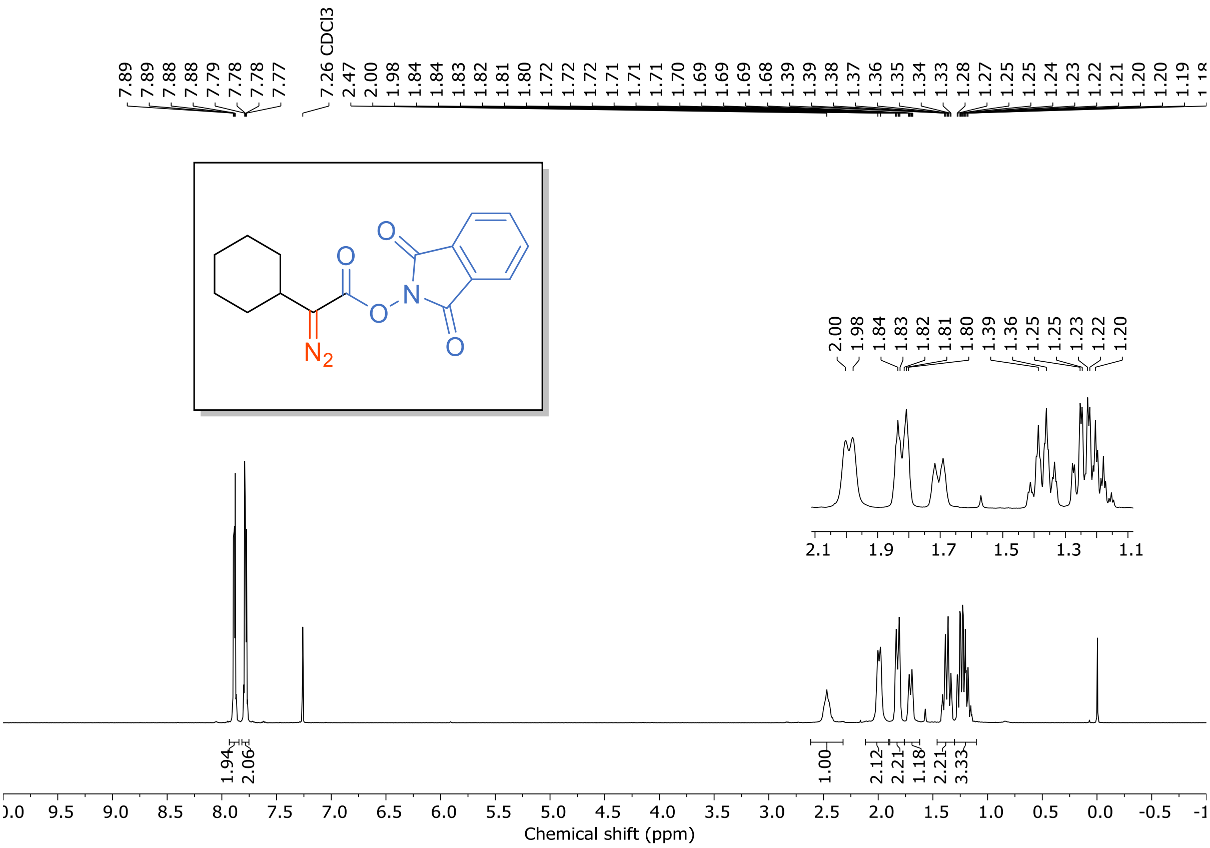

^{13}C NMR (126 MHz, $CDCl_3$)

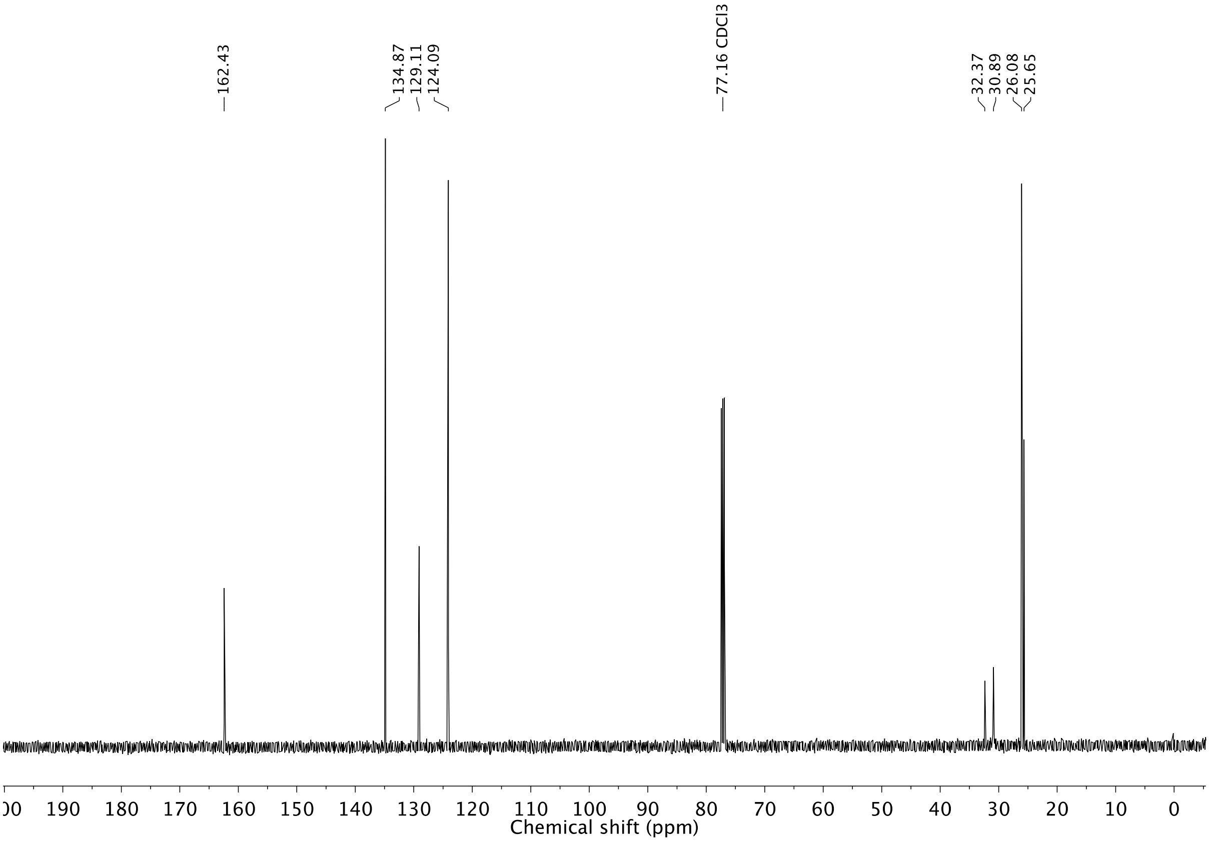

1,3-dioxoisoindolin-2-yl 2-diazo-3,3-dimethylbutanoate 4e

¹H NMR (500 MHz, CDCl₃)

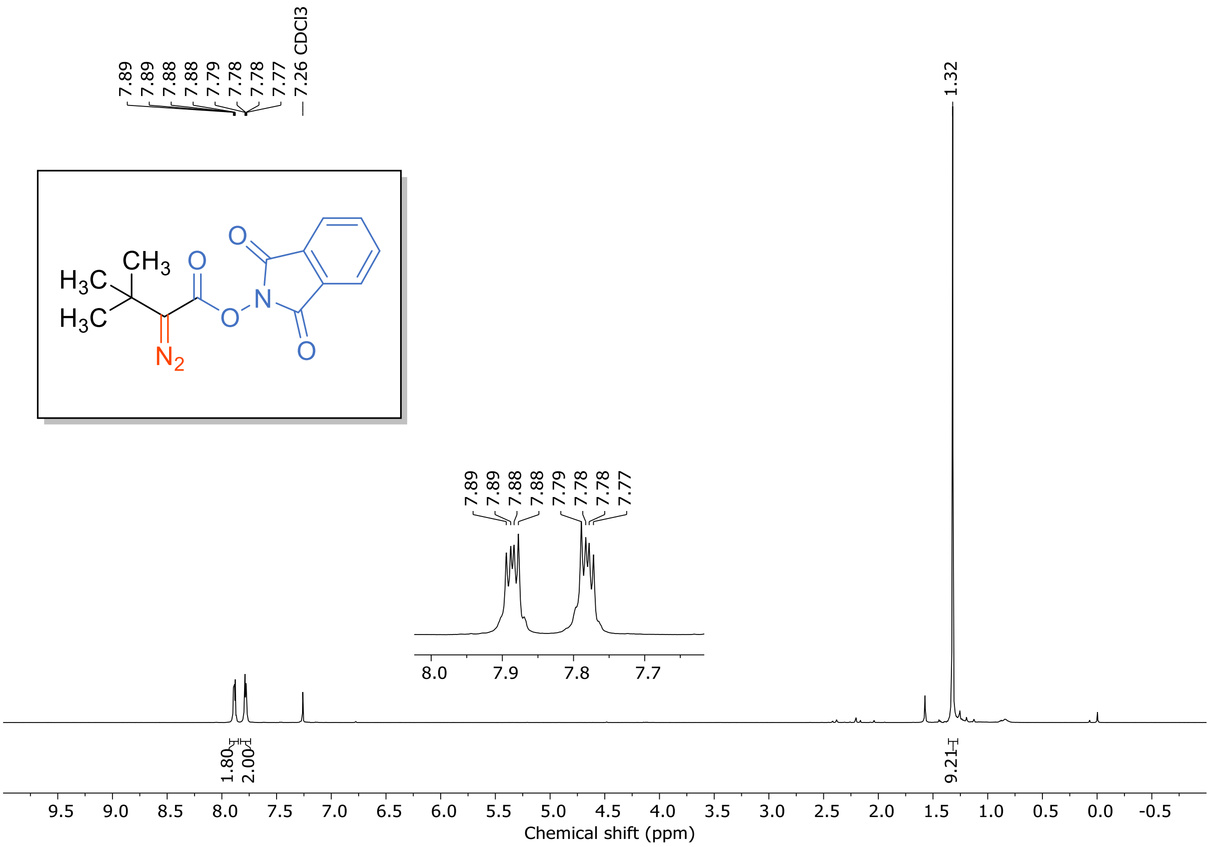

¹³C NMR (126 MHz, CDCl₃)

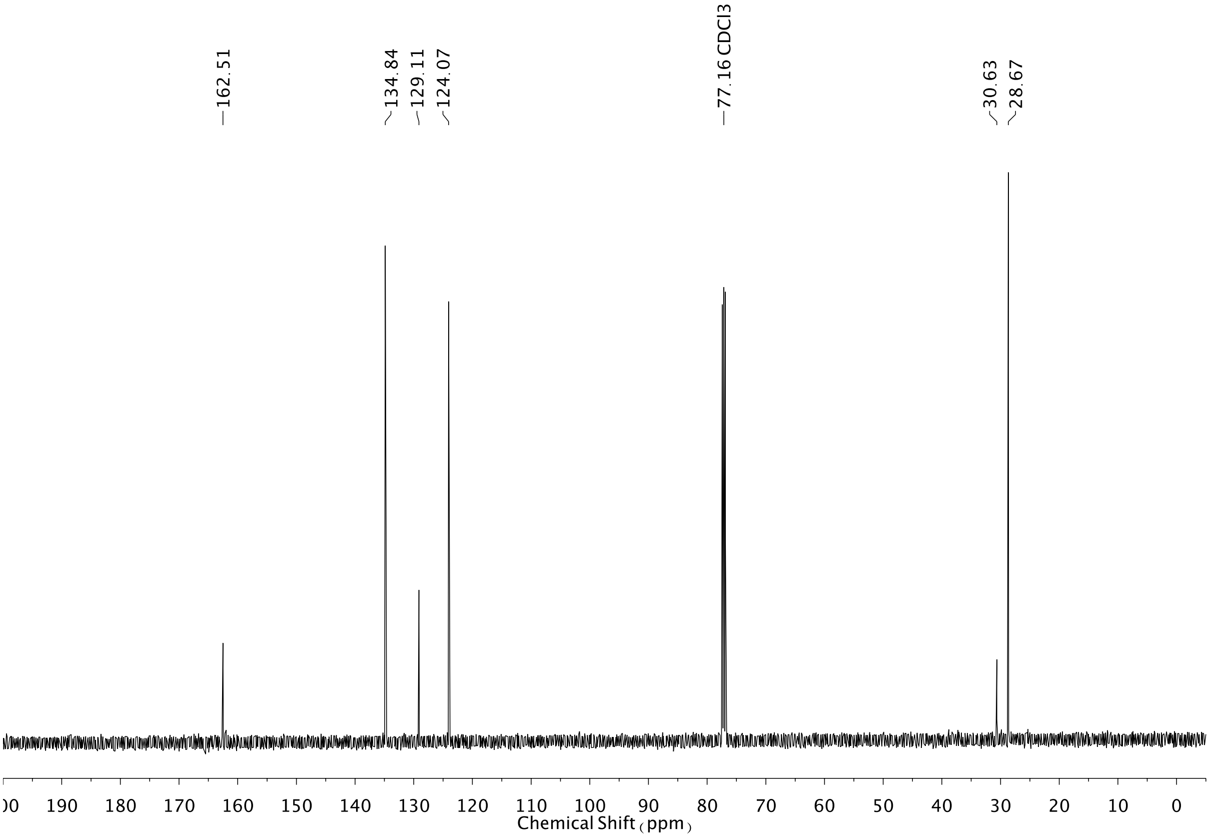

1,3-dioxoisoindolin-2-yl 2-diazo-3,3-dimethylpentanoate 4f

^{1}H NMR (500 MHz, CDCl$_3$)

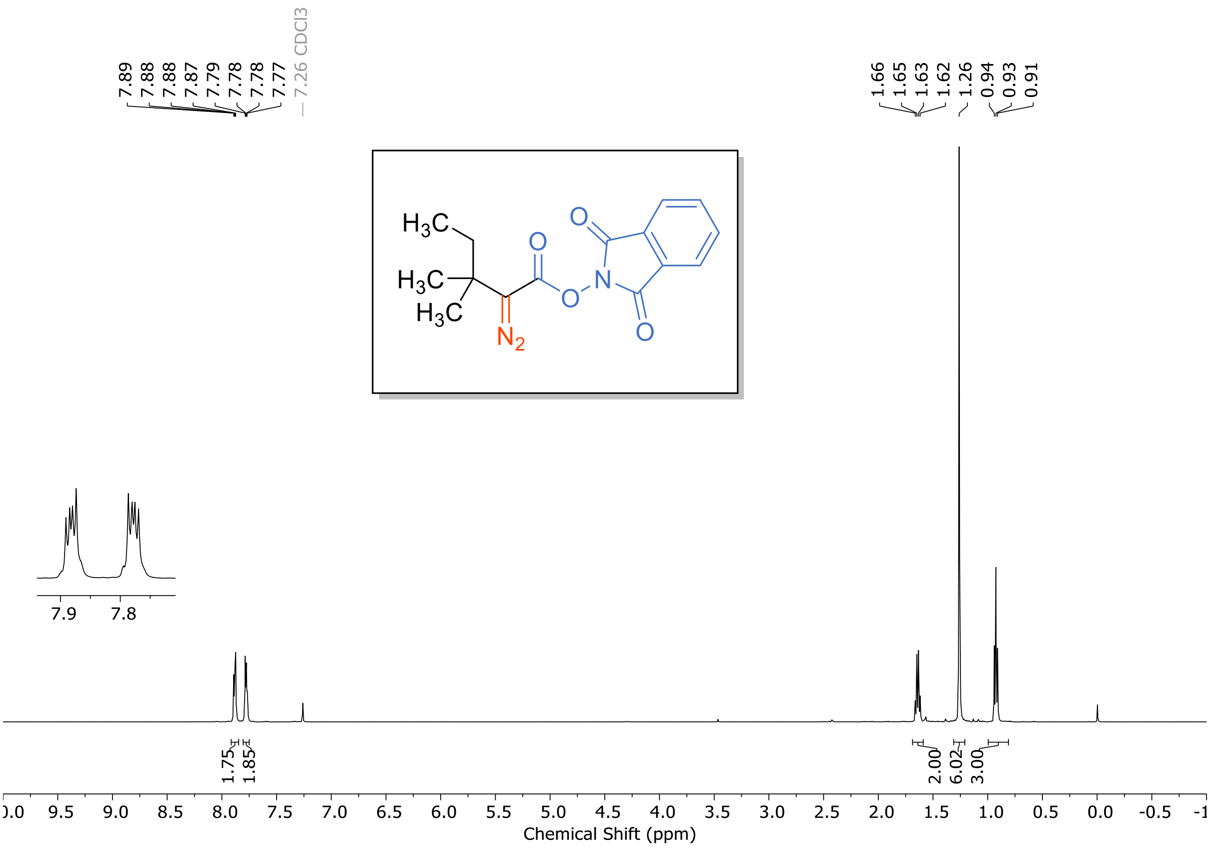

^{13}C NMR (126 MHz, CDCl$_3$)

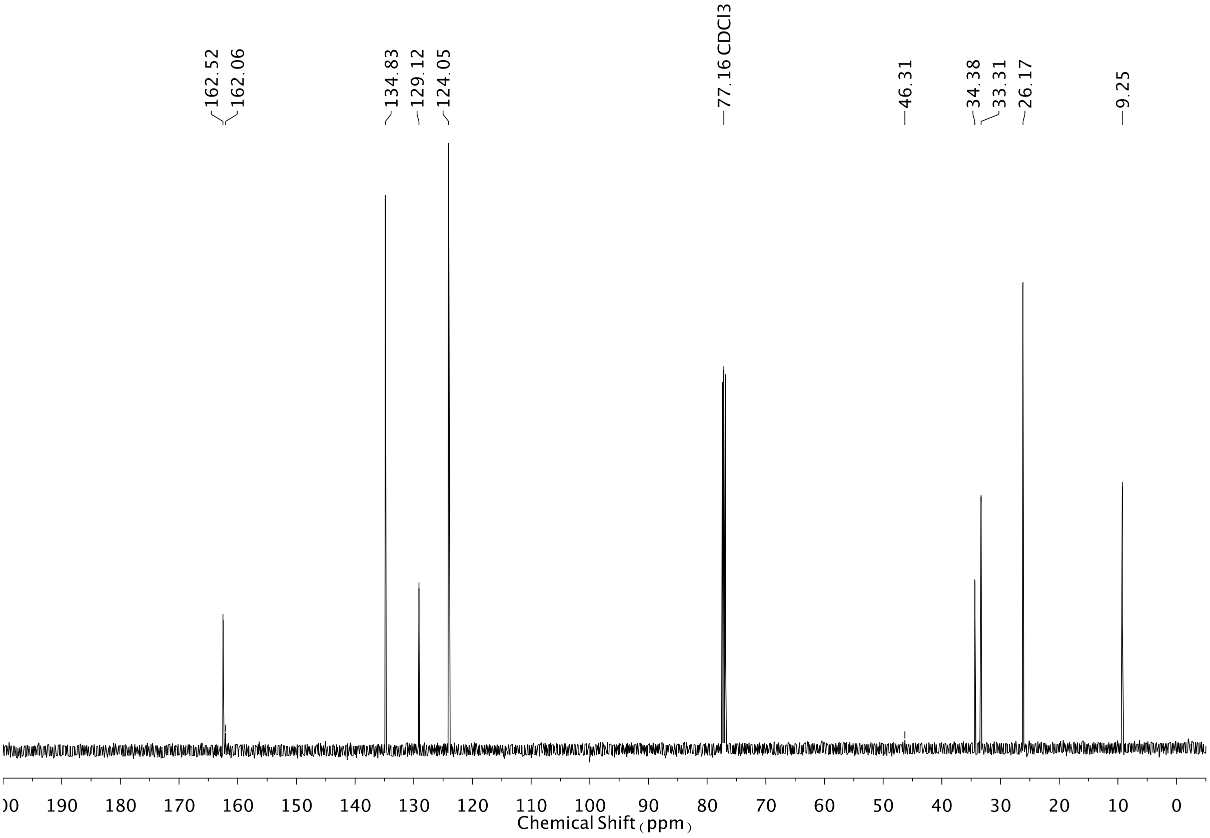

1,3-dioxoisoindolin-2-yl 2-diazo-2-(4-methoxyphenyl)acetate 3c

^{1}H NMR (500 MHz, $CDCl_3$)

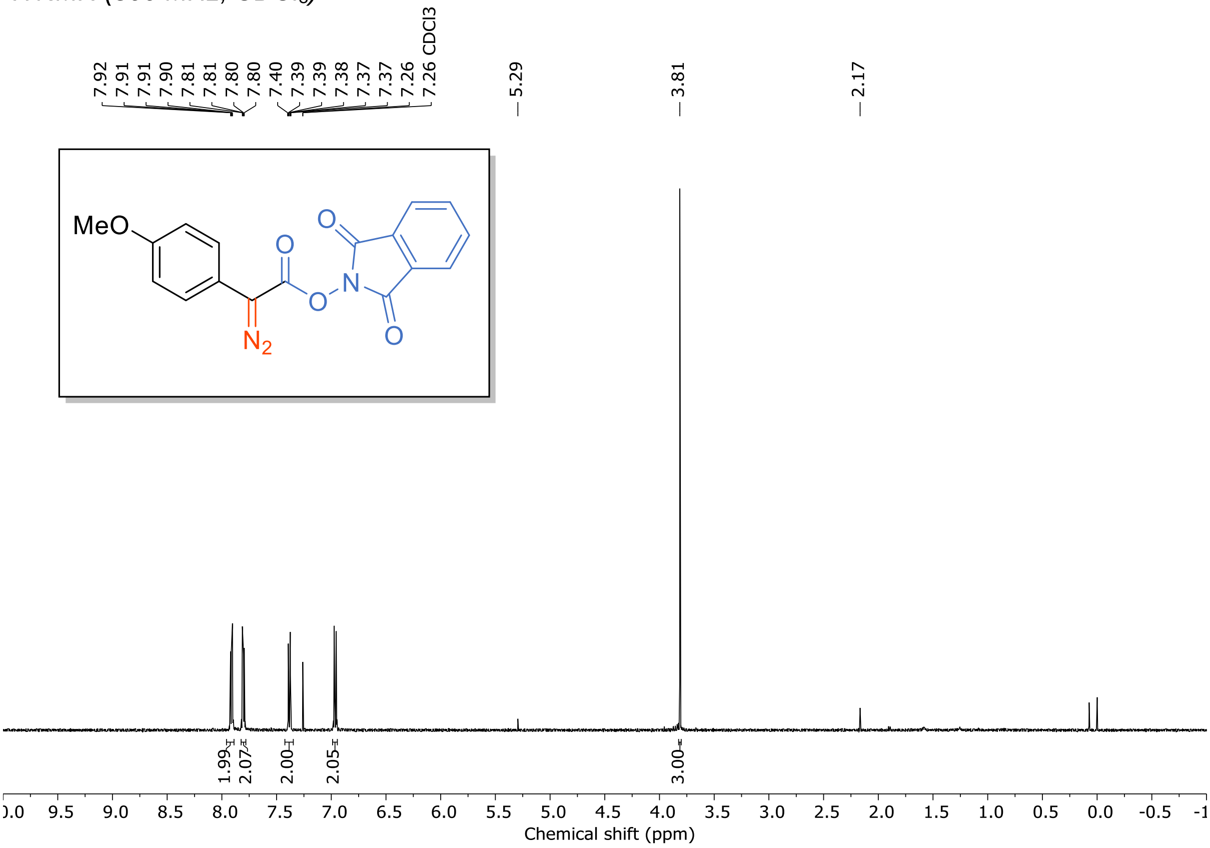

^{13}C NMR (126 MHz, $CDCl_3$)

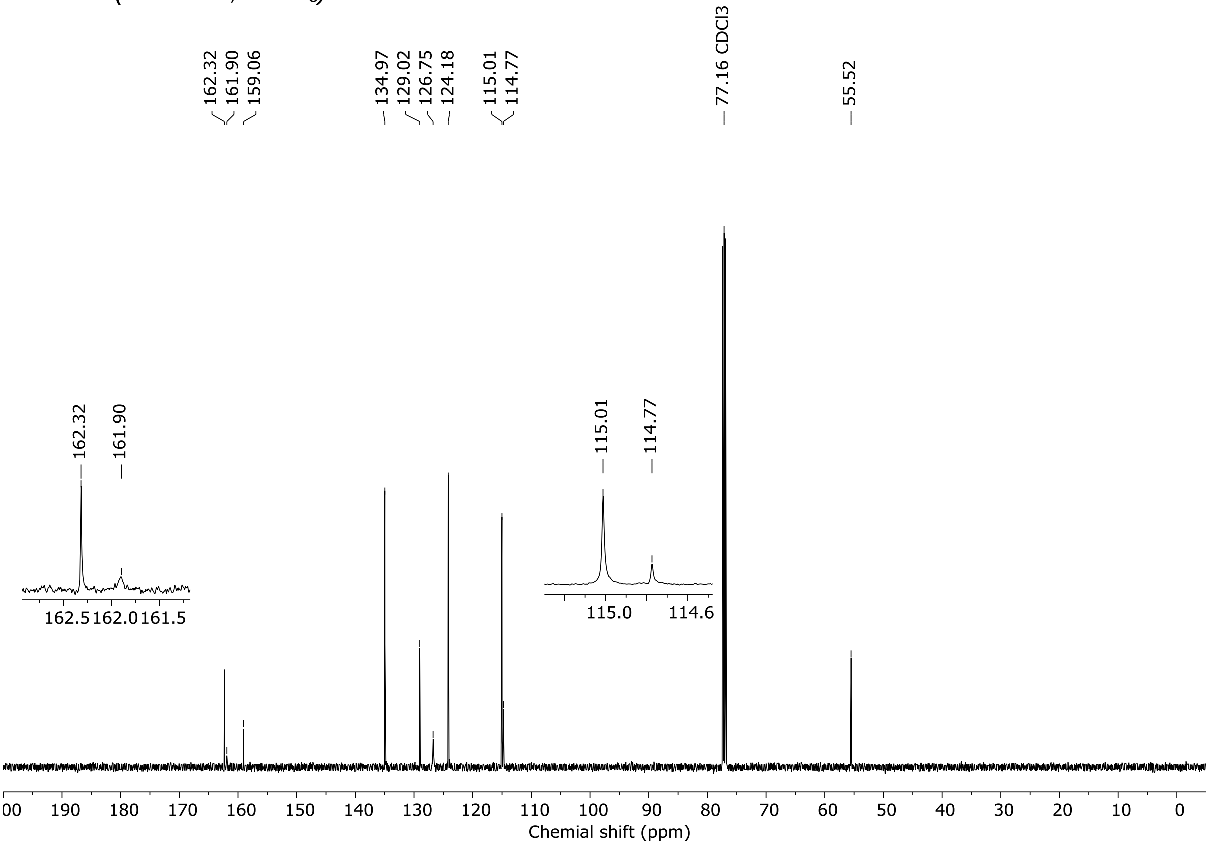

1,3-dioxoisoindolin-2-yl 2-diazo-2-(p-tolyl)acetate 3d

^{1}H NMR (400 MHz, CDCl$_3$)

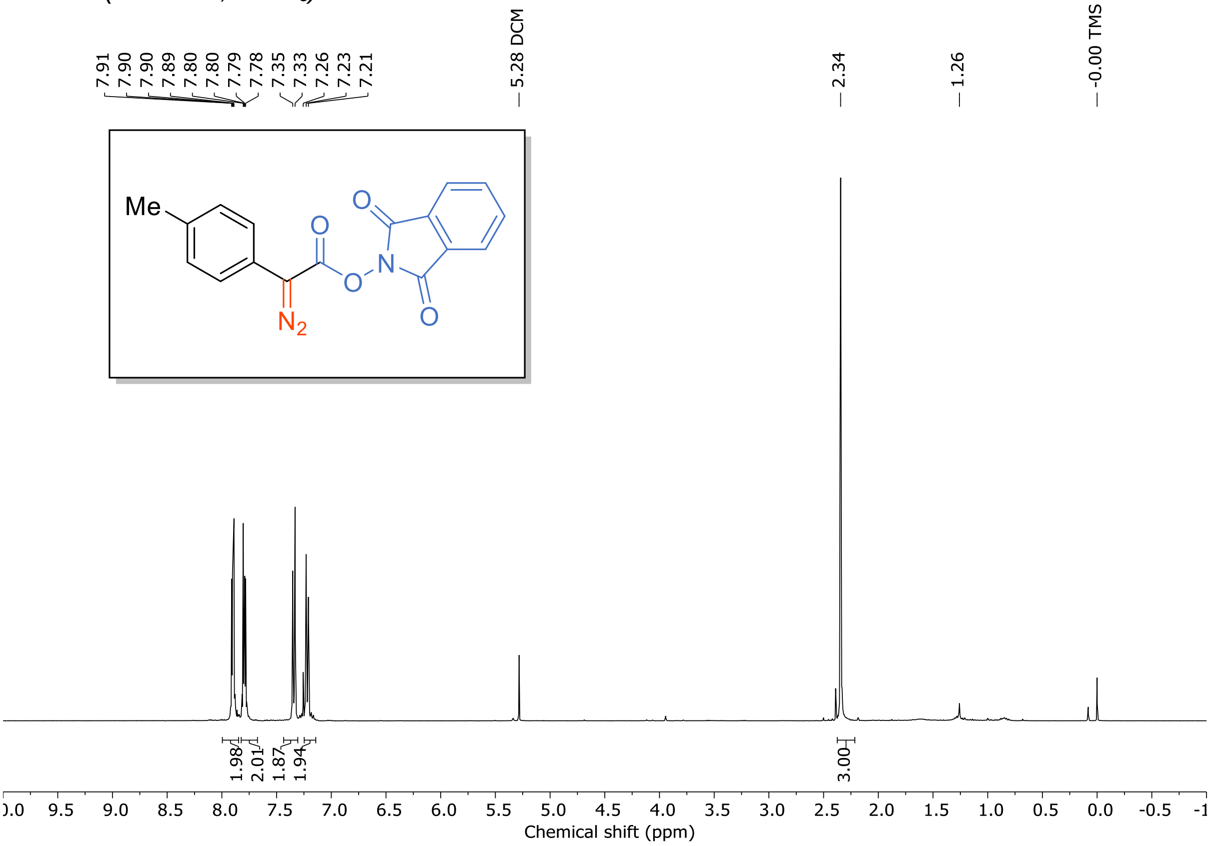

^{13}C NMR (101 MHz, CDCl$_3$)

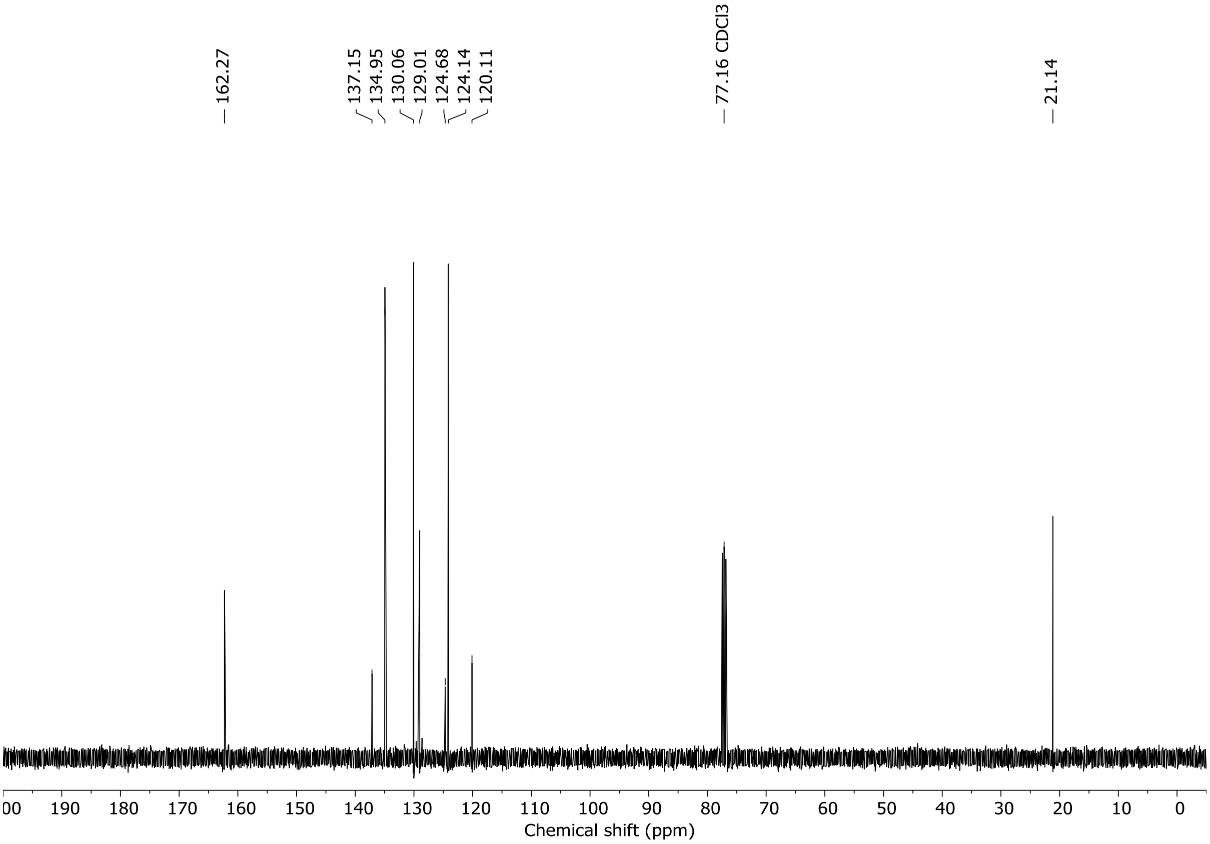

methyl 4-(1-diazo-2-((1,3-dioxoisoindolin-2-yl)oxy)-2-oxoethyl)benzoate 3e

^{1}H NMR (500 MHz, $CDCl_3$)

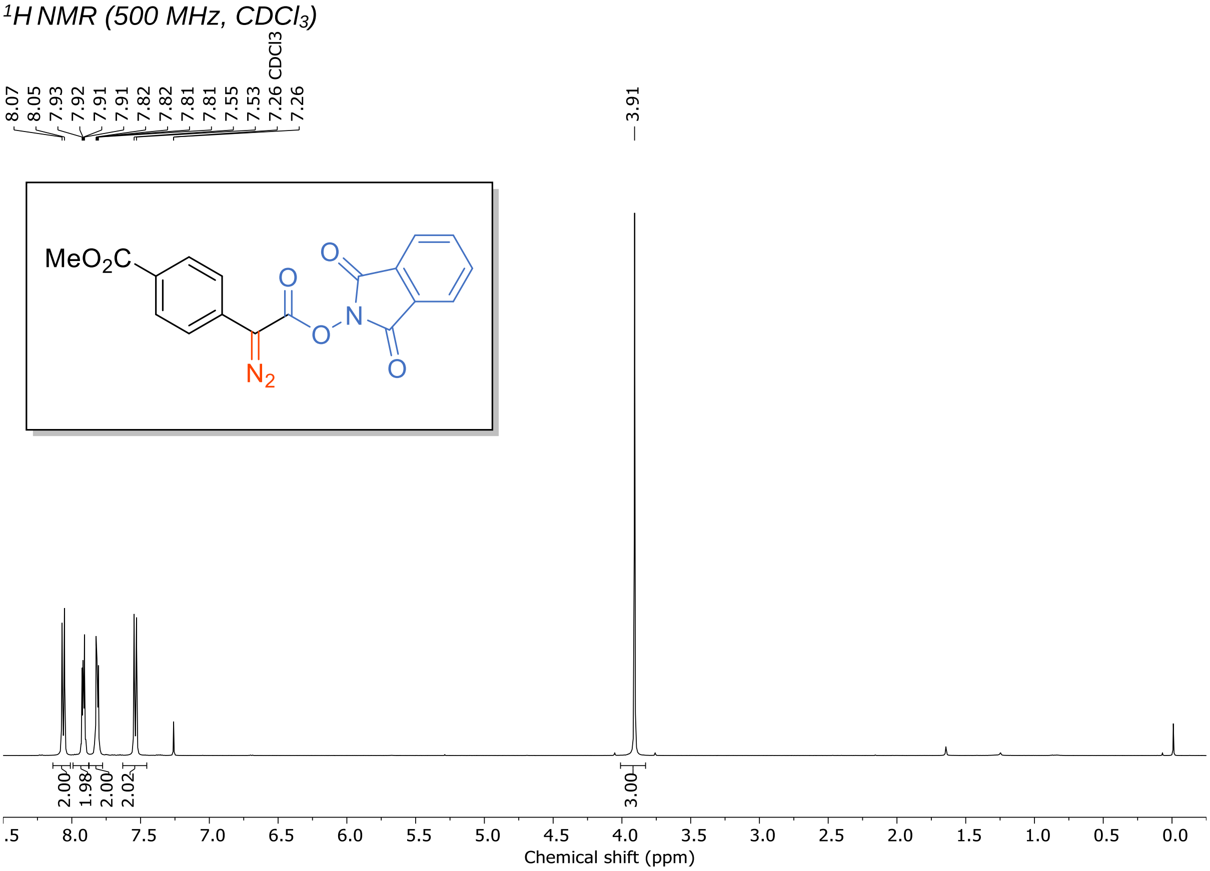

^{13}C NMR (126 MHz, $CDCl_3$)

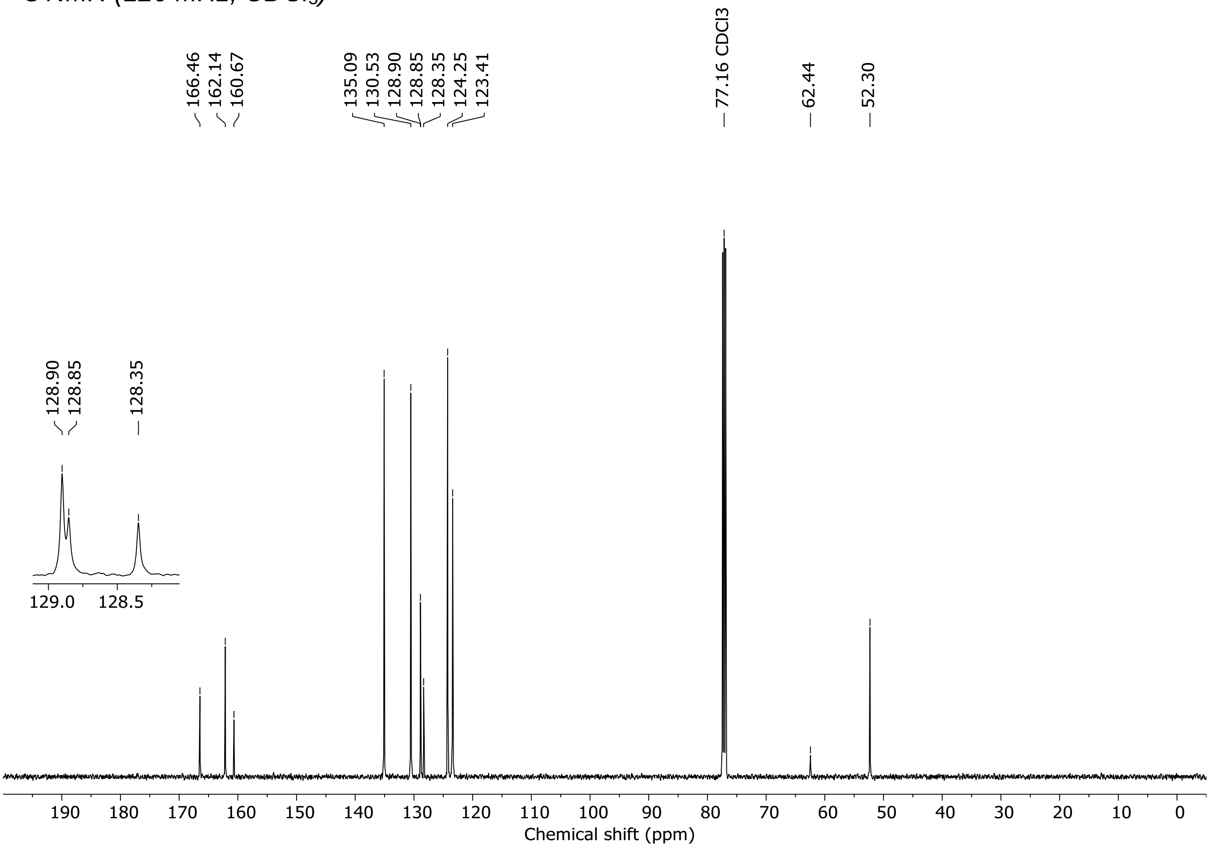

1,3-dioxoisoindolin-2-yl 2-cyclohexyl-2-phenylacetate 5a

^{1}H NMR (500 MHz, $CDCl_3$)

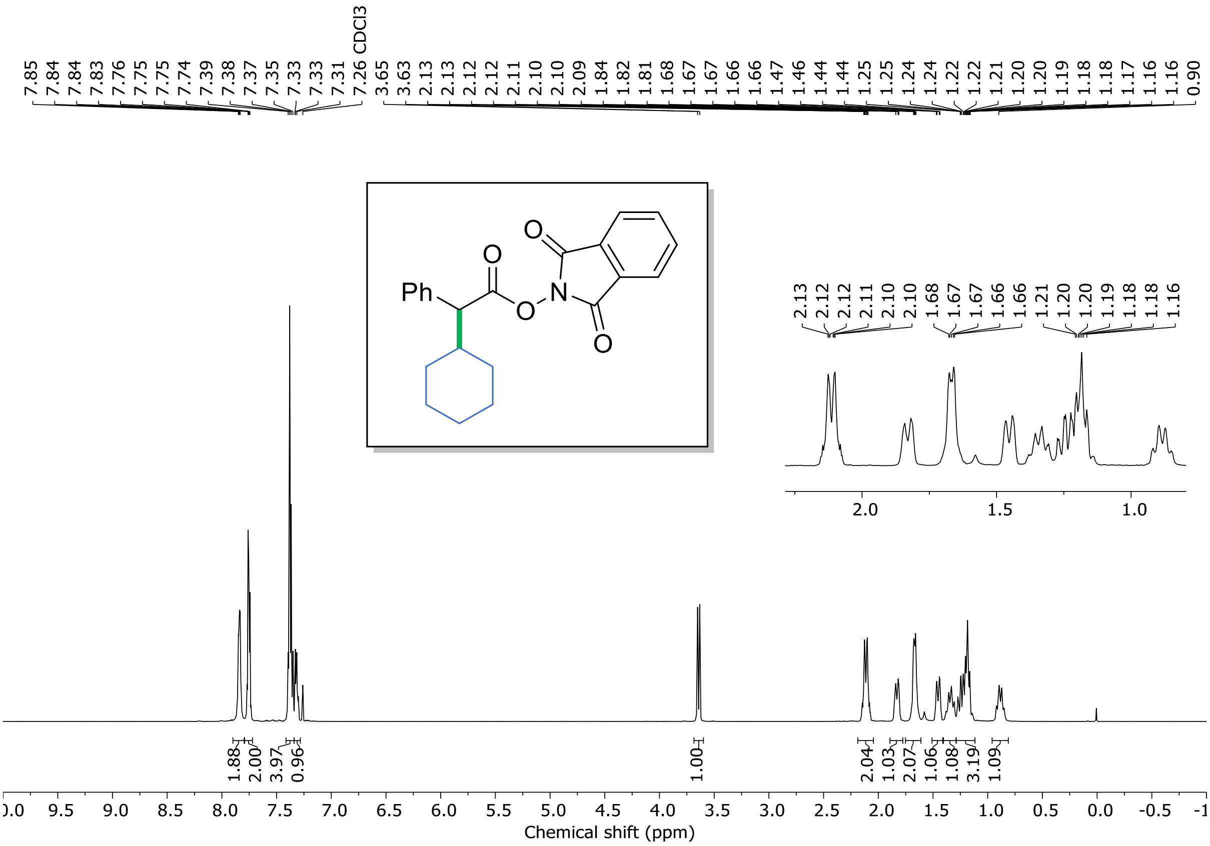

^{13}C NMR (126 MHz, $CDCl_3$)

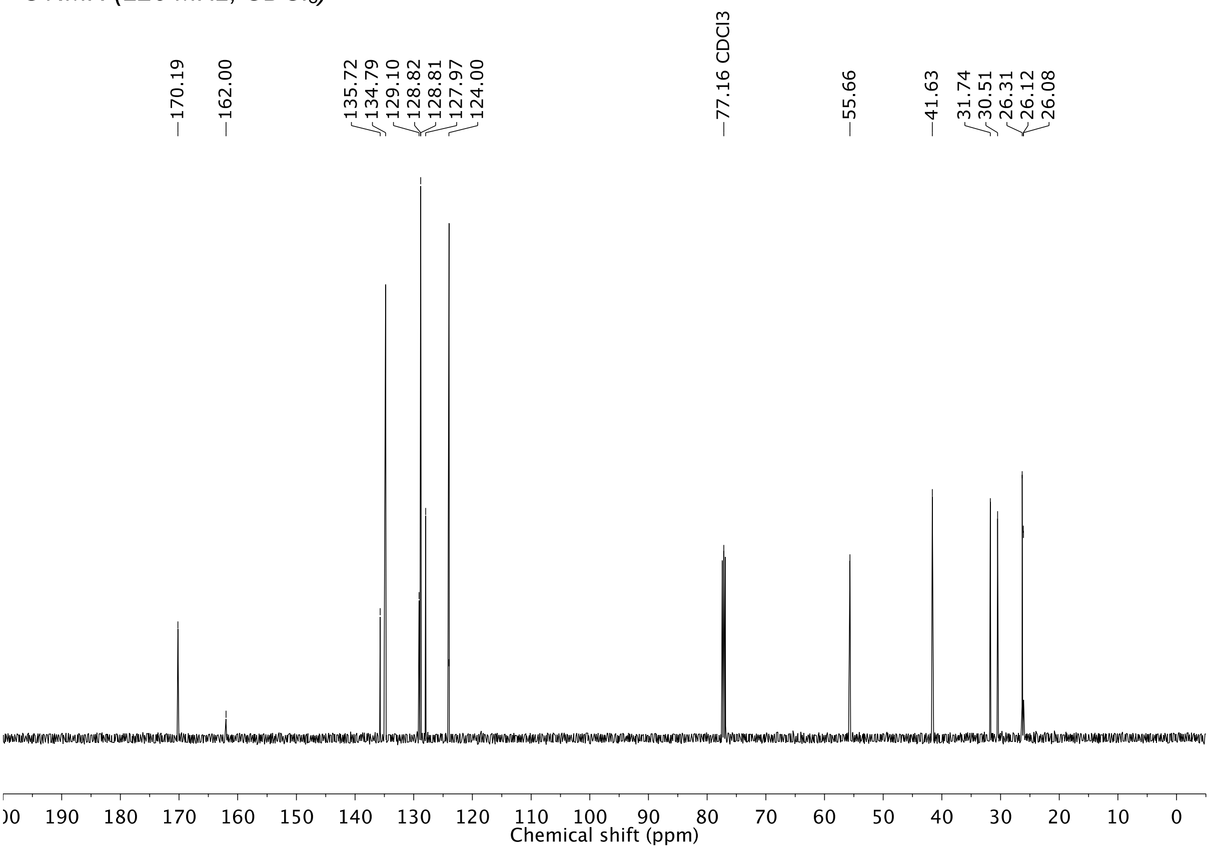

ethyl 2-cyclohexyl-2-phenylacetate 5a'

^{1}H NMR (500 MHz, $CDCl_3$)

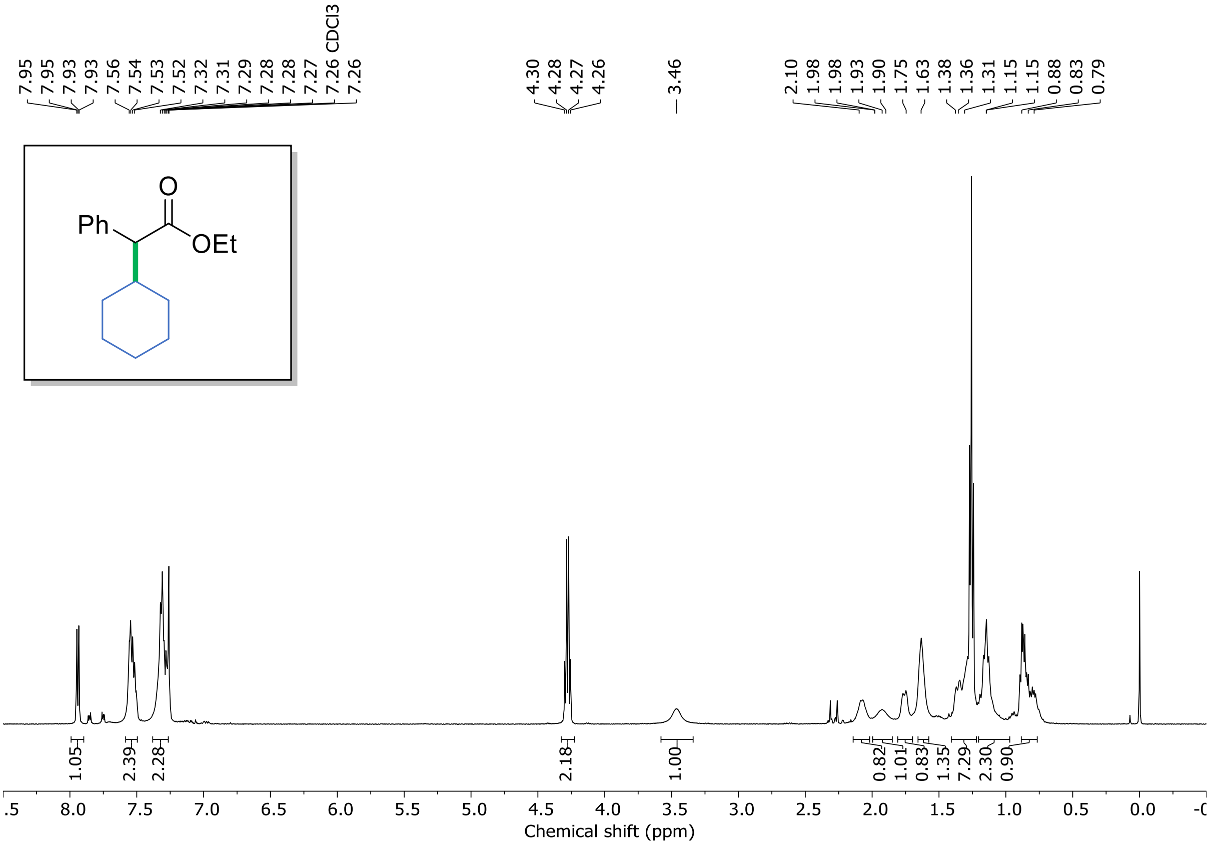

^{13}C NMR (126 MHz, $CDCl_3$)

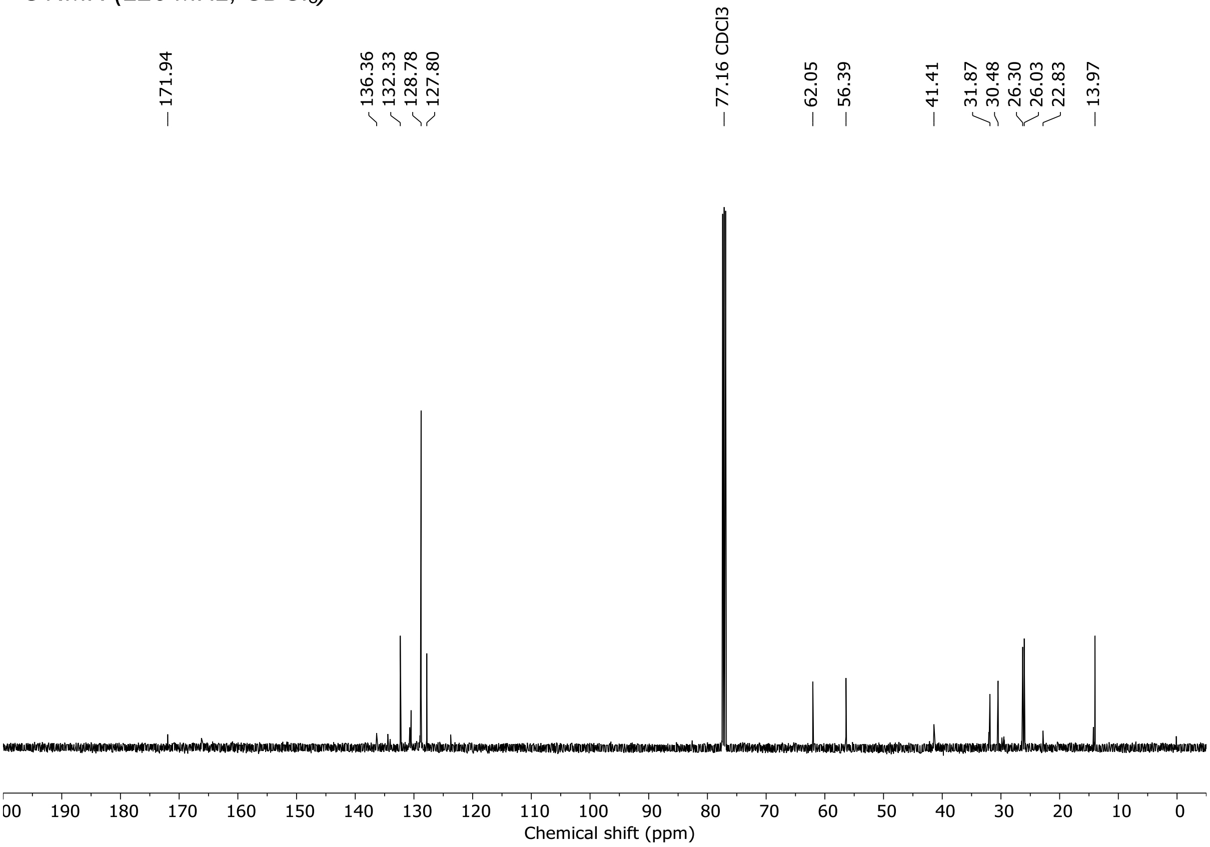

1,3-dioxoisoindolin-2-yl 2-cyclohexyl-2-phenylacetate 5a’’

^{1}H NMR (500 MHz, $CDCl_3$)

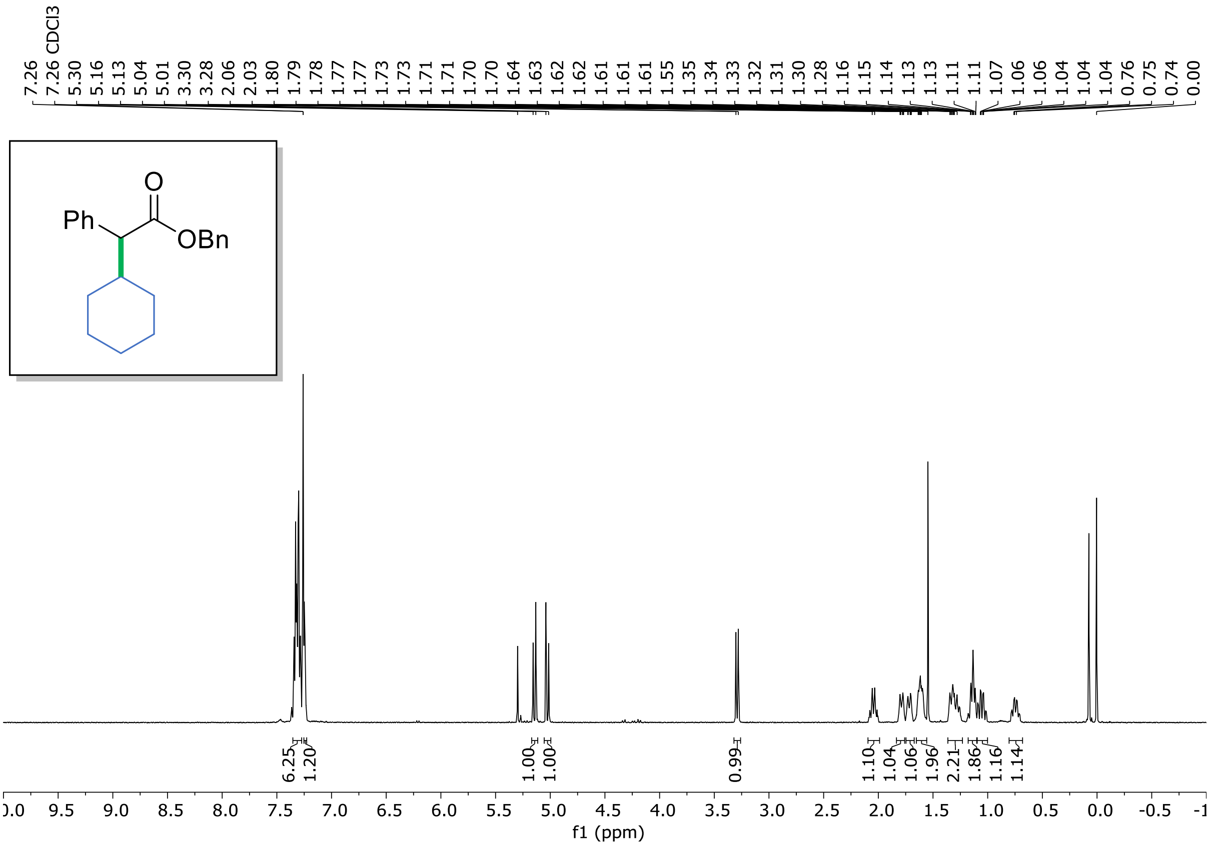

^{13}C NMR (126 MHz, $CDCl_3$)

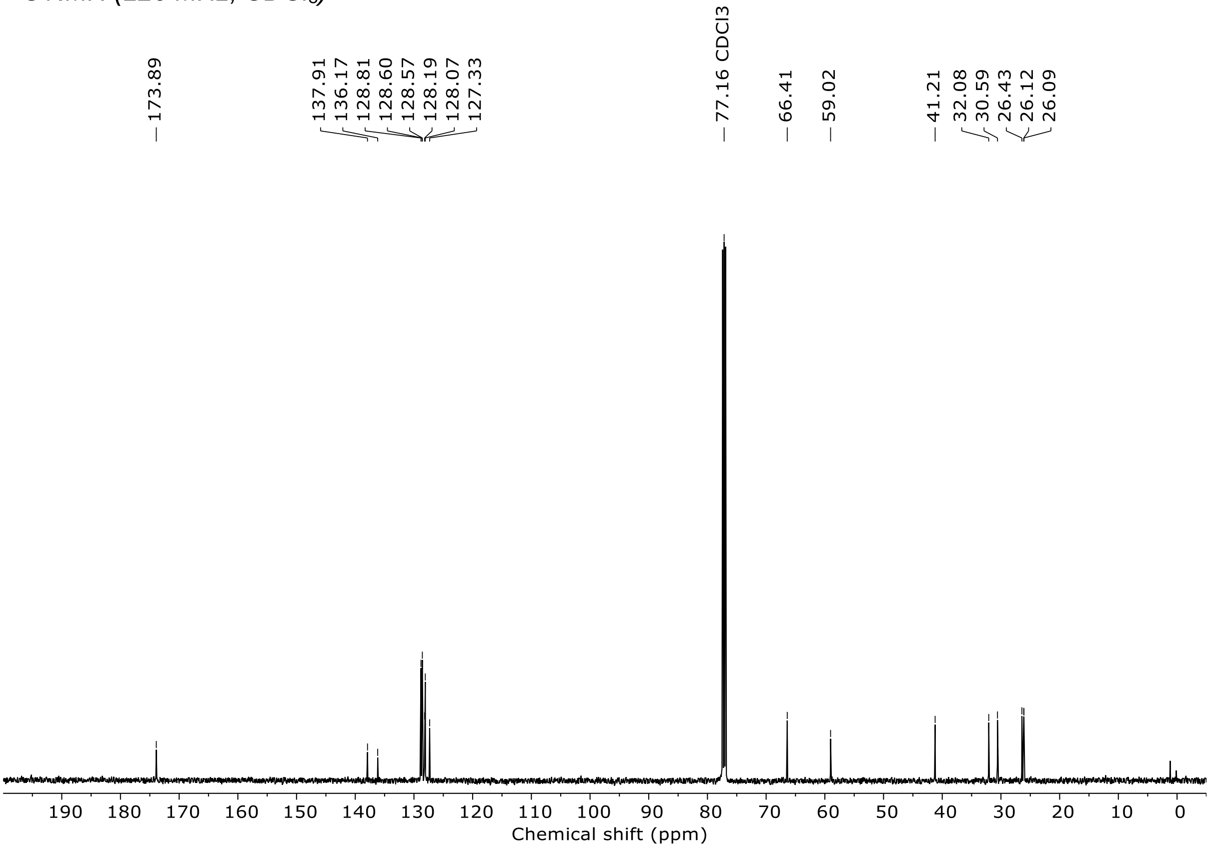

1,3-dioxoisoindolin-2-yl 2-cyclopentyl-2-phenylacetate 5b

^{1}H NMR (500 MHz, $CDCl_3$)

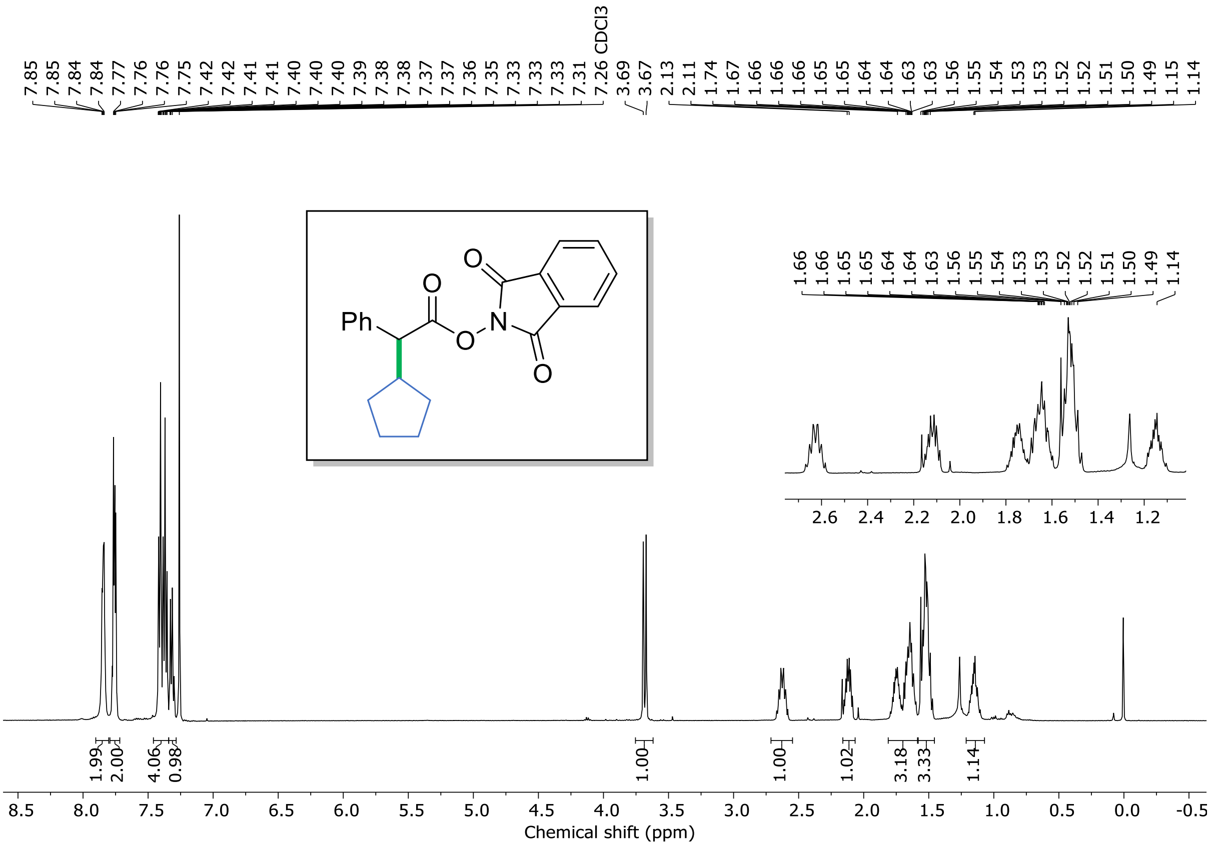

^{13}C NMR (126 MHz, $CDCl_3$)

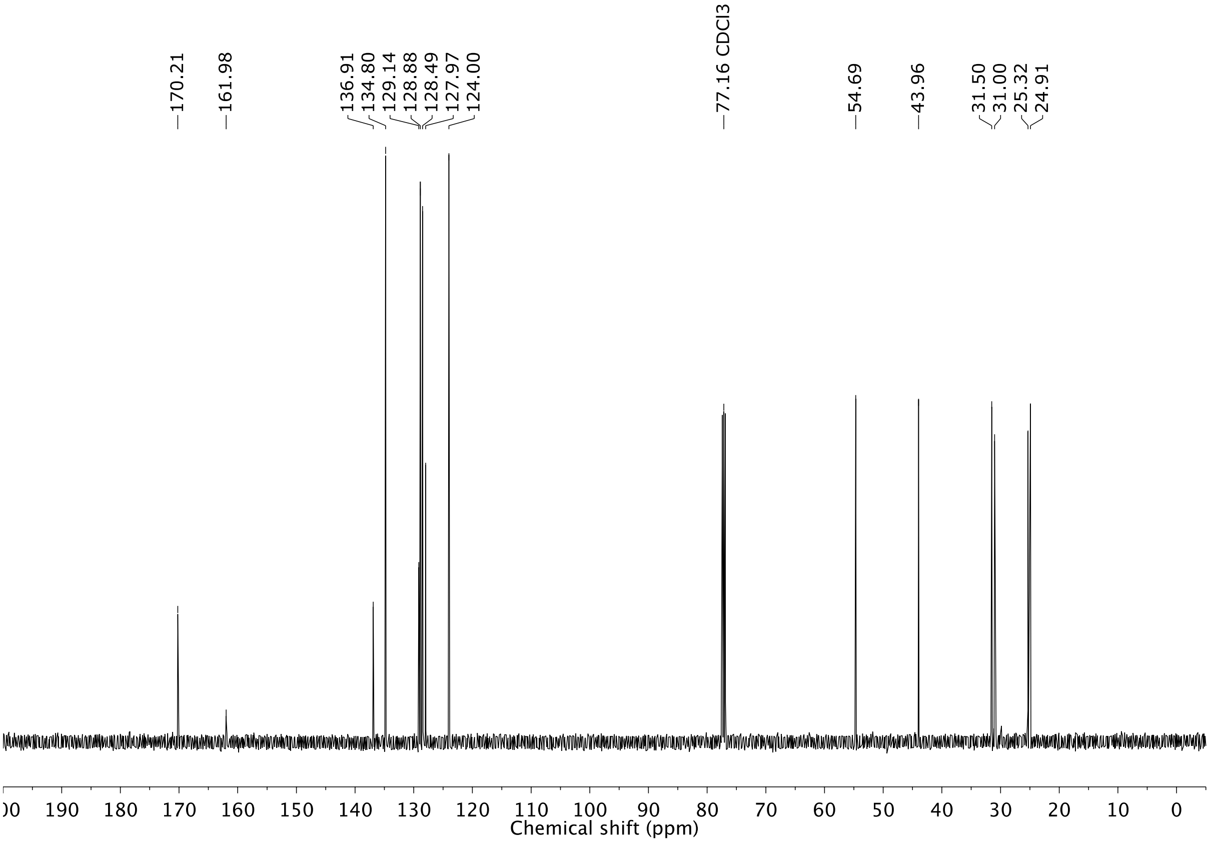

1,3-dioxoisoindolin-2-yl (S)-2-cyclooctyl-2-phenylacetate 5c

^{1}H NMR (500 MHz, $CDCl_3$)

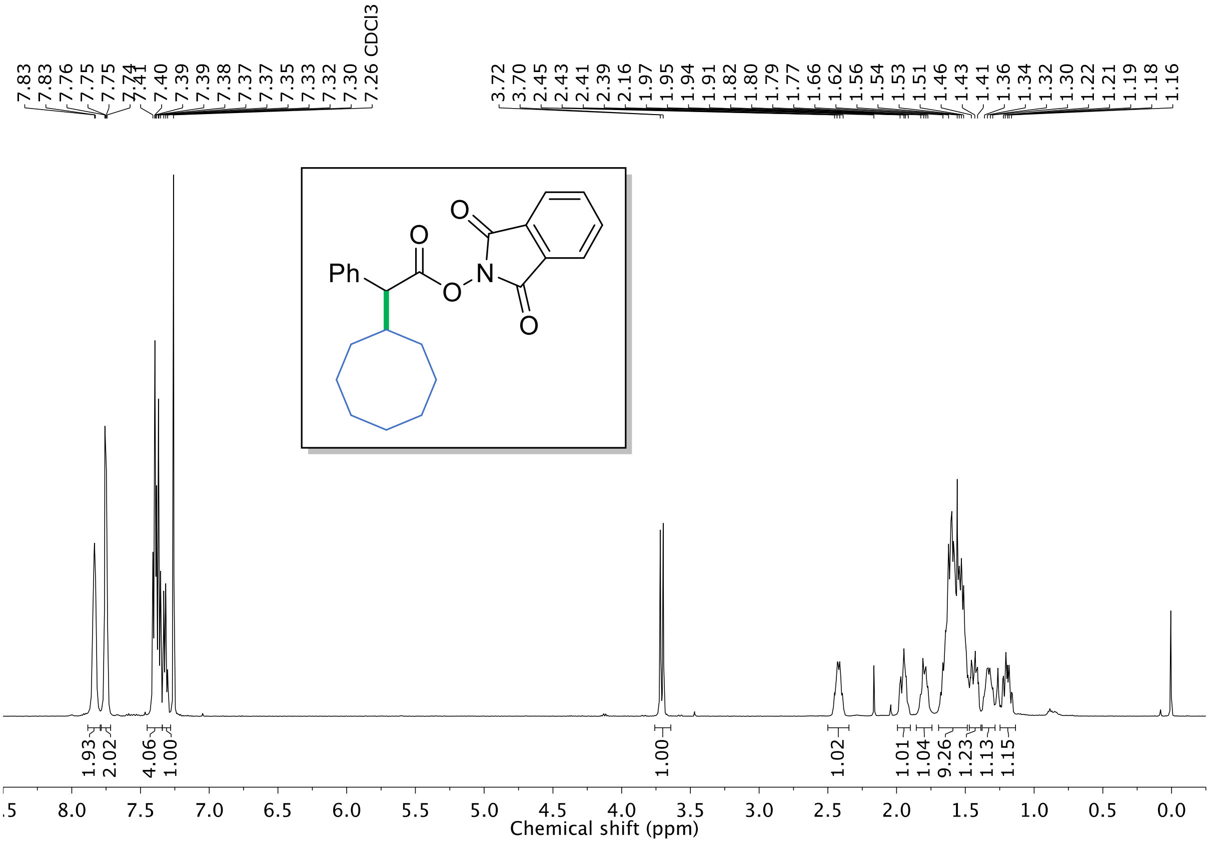

^{13}C NMR (126 MHz, $CDCl_3$)

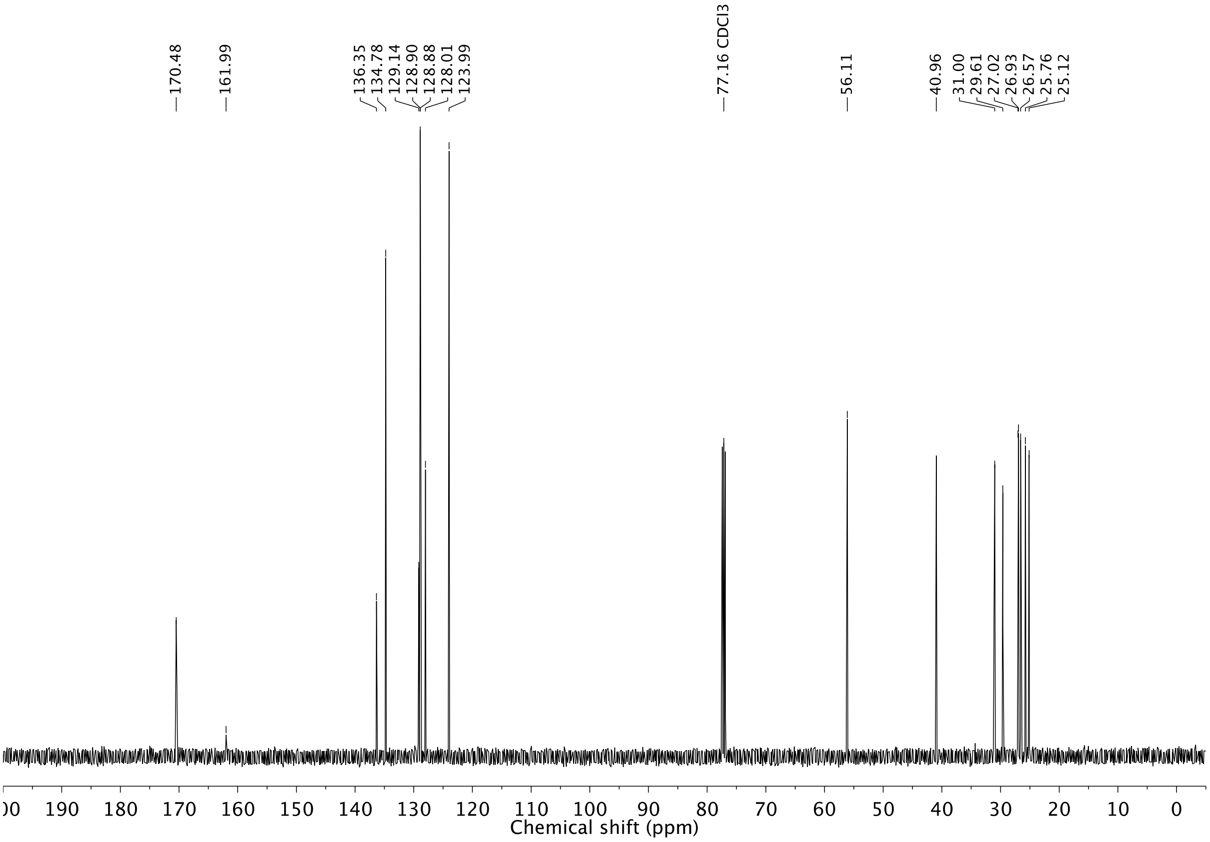

1,3-dioxoisoindolin-2-yl (S)-2-((3S,5S,7S)-adamantan-1-yl)-2-phenylacetate 5d

^{1}H NMR (500 MHz, $CDCl_3$)

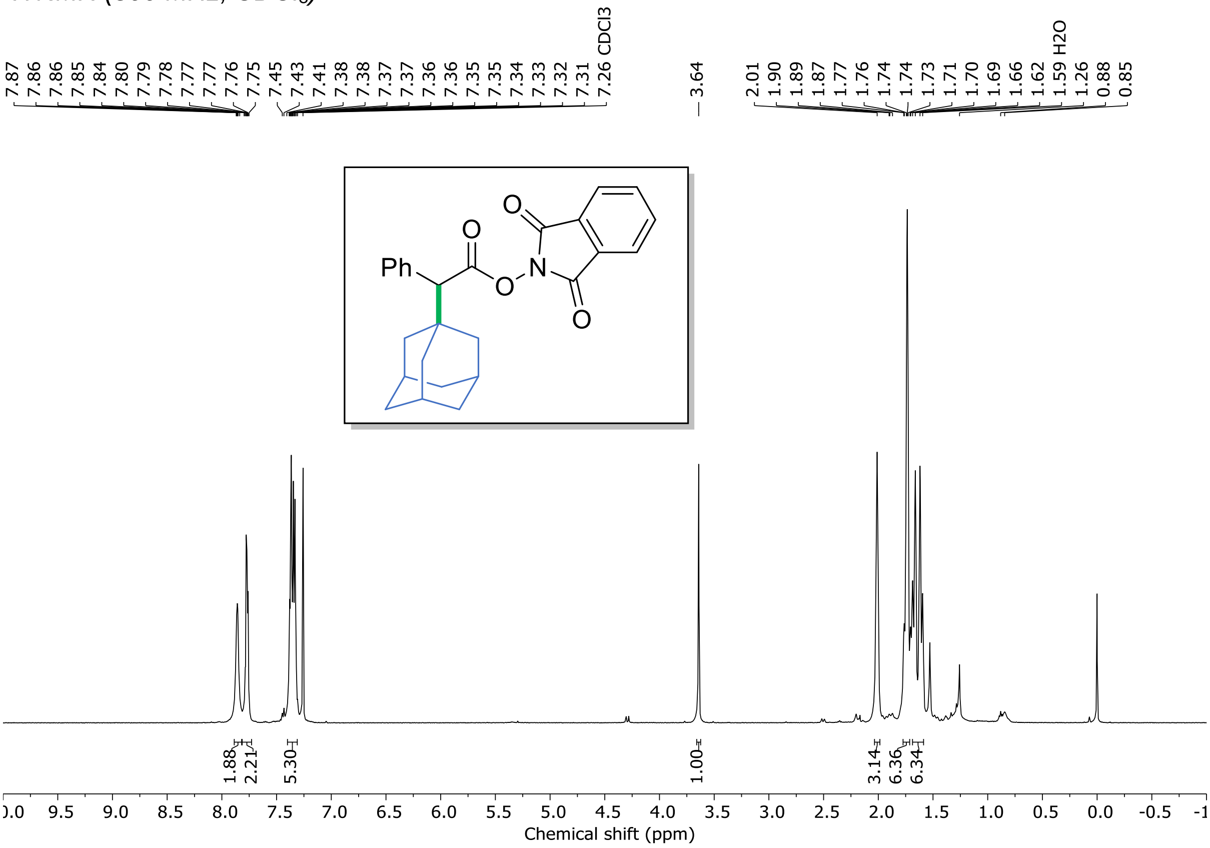

^{13}C NMR (126 MHz, $CDCl_3$)

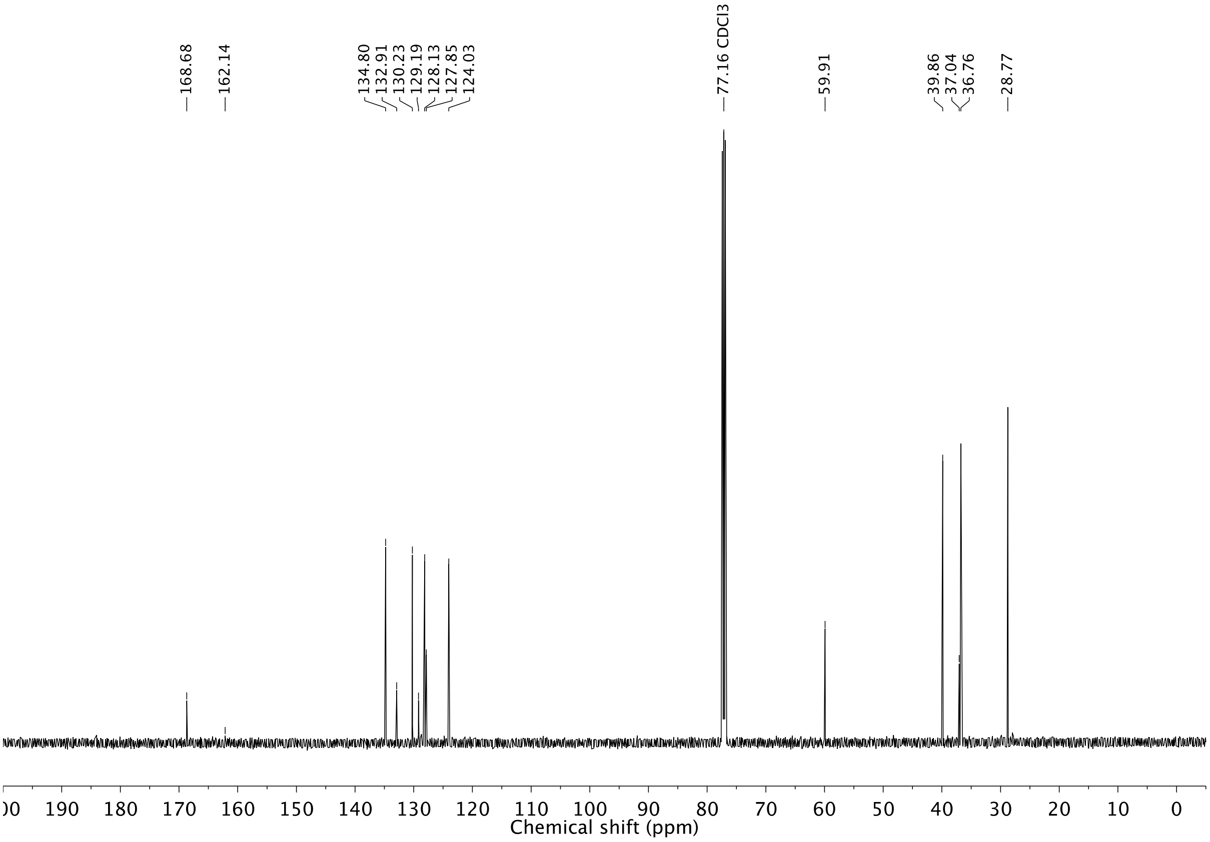

1,3-dioxoisoindolin-2-yl (R)-2-((R)-1,4-dioxan-2-yl)-2-phenylacetate 5e

^{1}H NMR (500 MHz, CDCl$_3$)

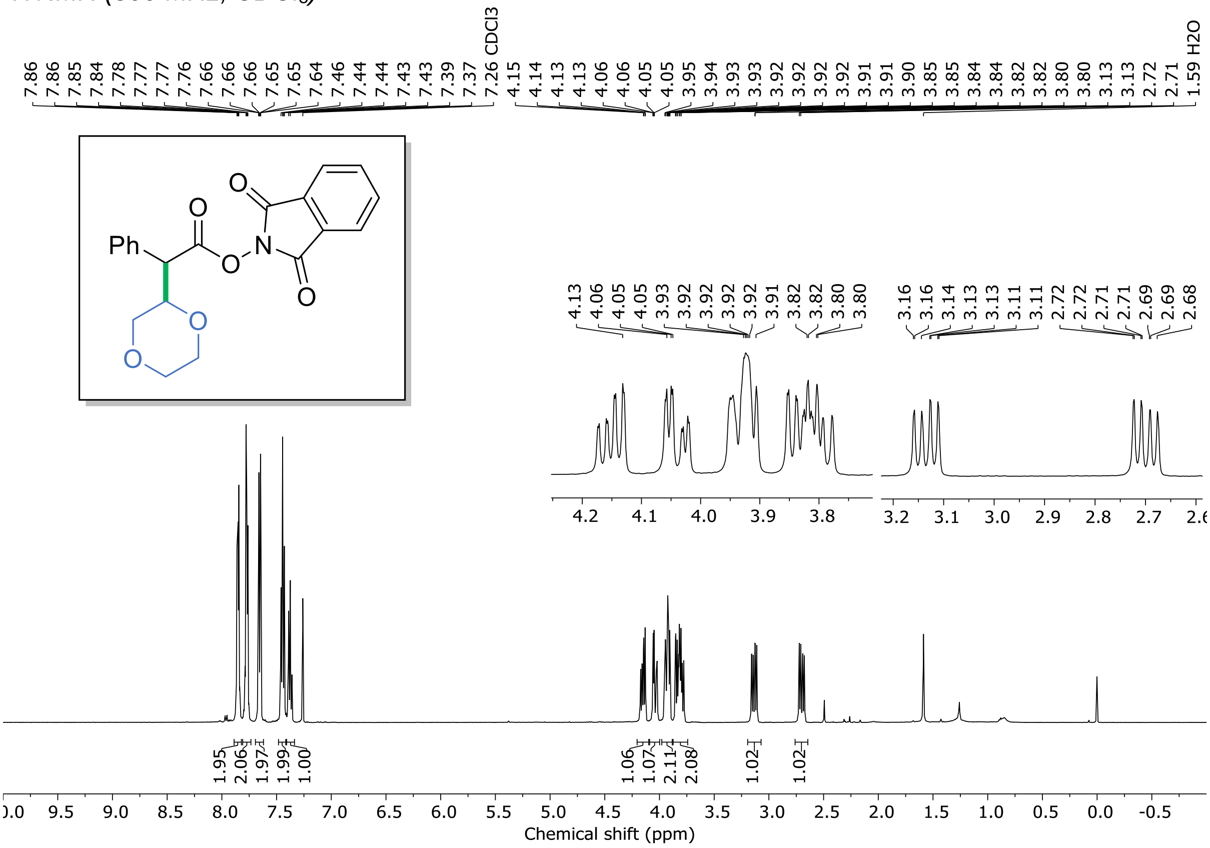

^{13}C NMR (126 MHz, CDCl$_3$)

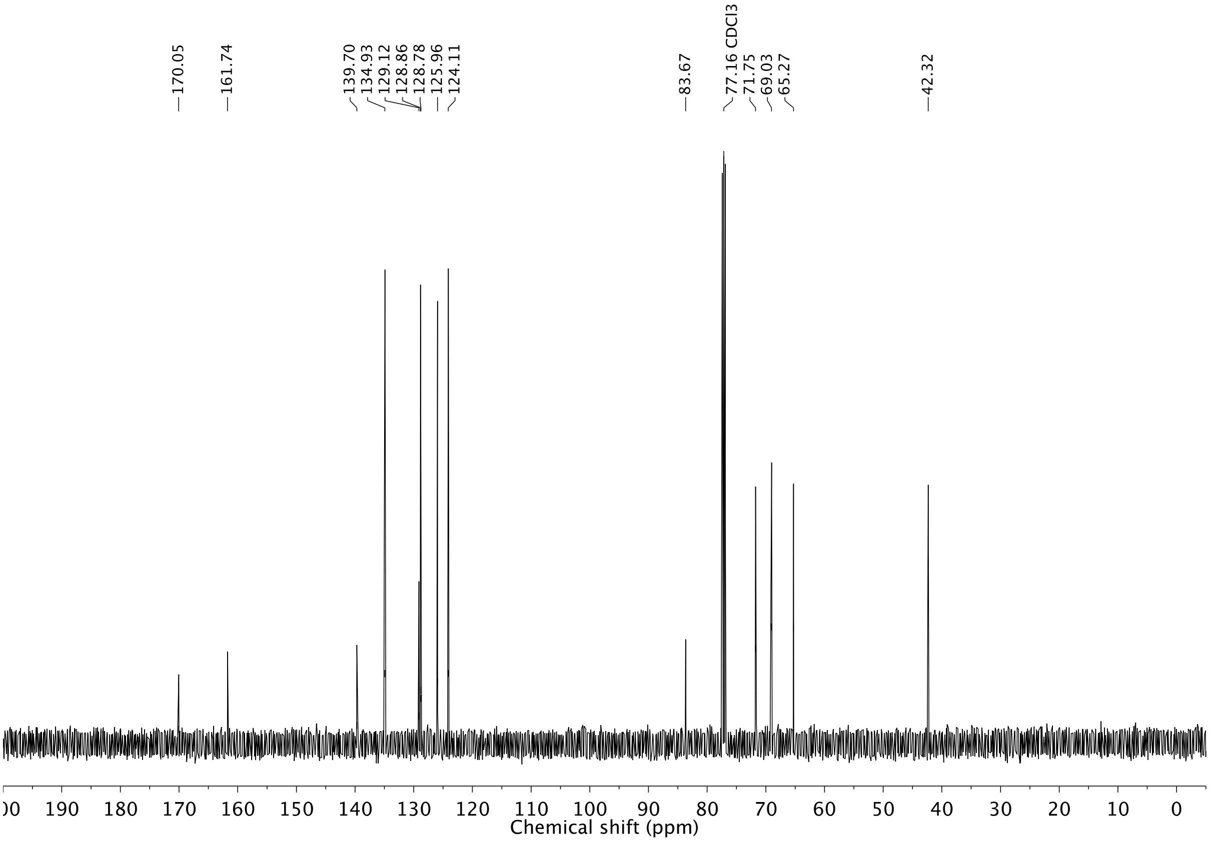

1,3-dioxoisoindolin-2-yl 2-cyclohexyl-2-(4-methoxyphenyl)acetate 5f

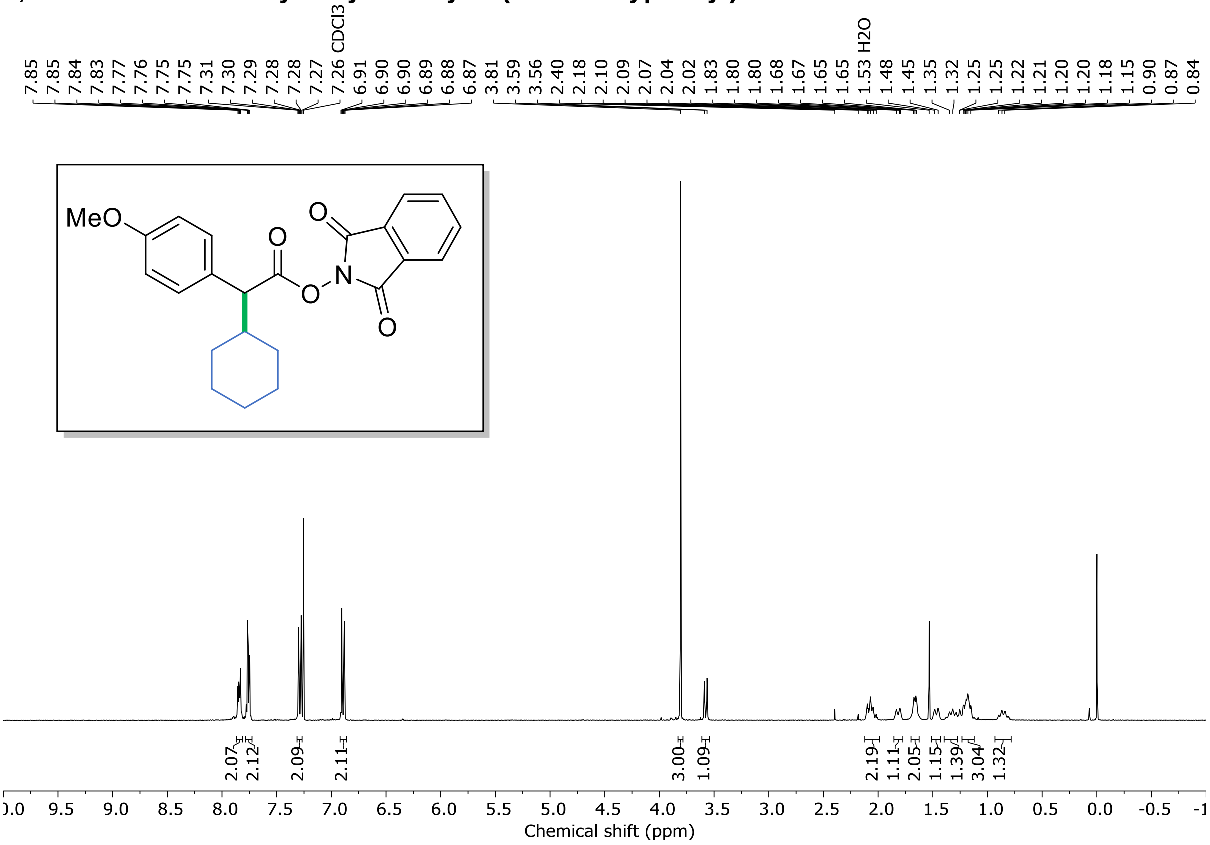

^{13}C *NMR (126 MHz, $CDCl_3$)*

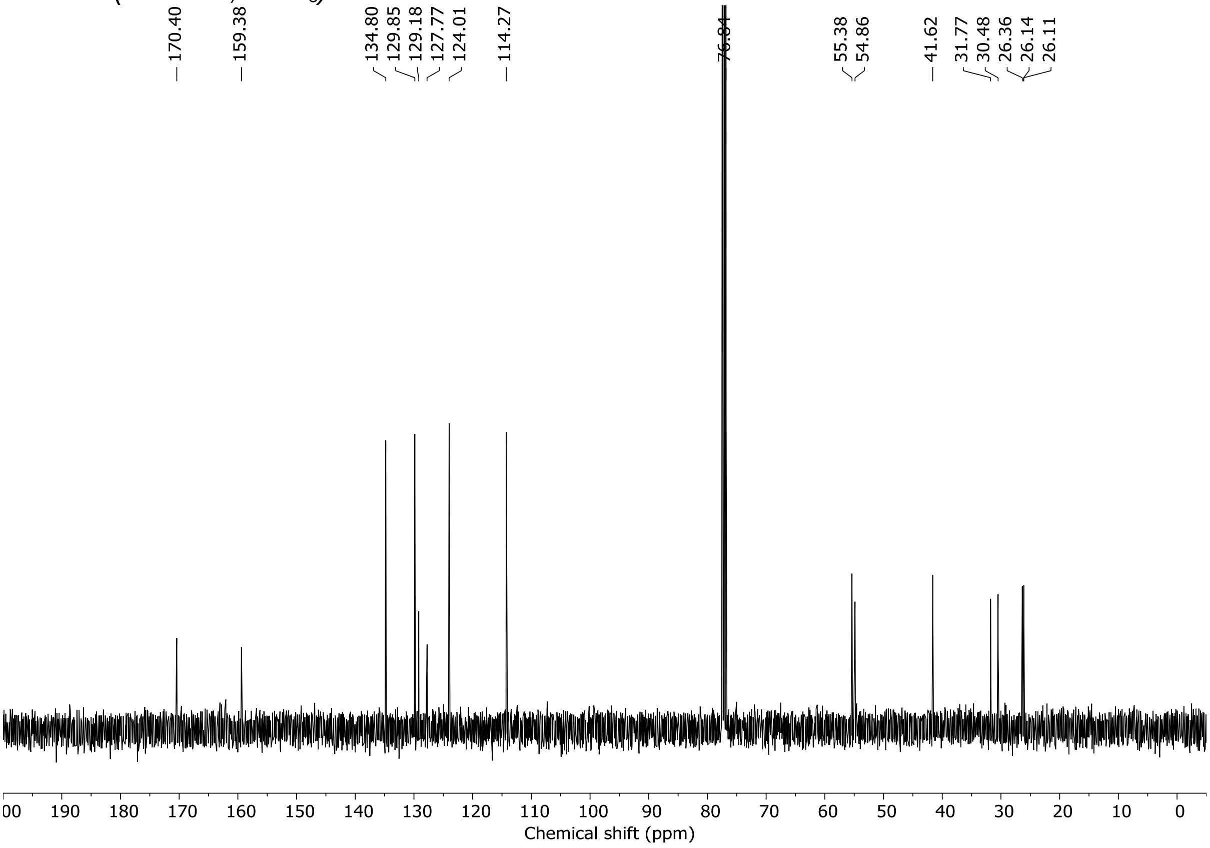

1,3-dioxoisoindolin-2-yl 2-cyclohexyl-2-(p-tolyl)acetate 5g

^{1}H NMR (500 MHz, CDCl$_3$)

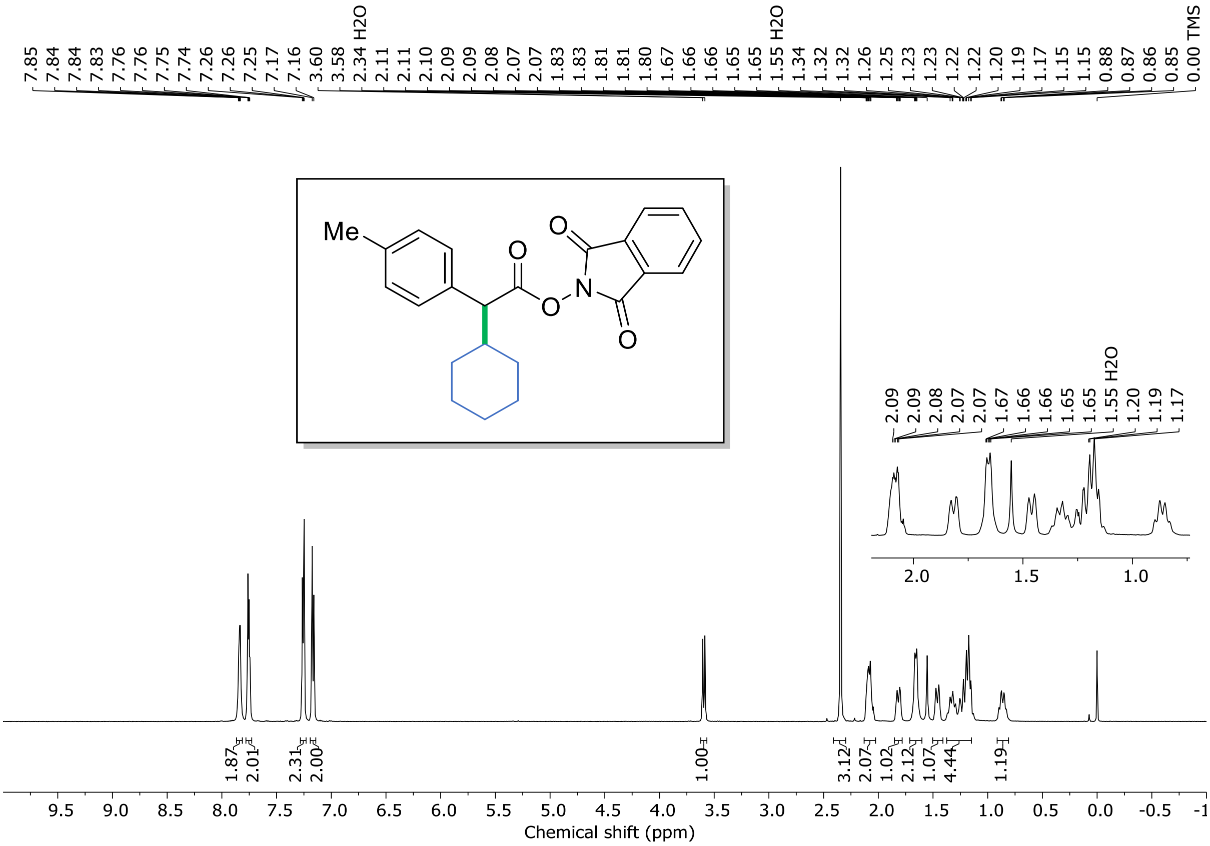

^{13}C NMR (126 MHz, CDCl$_3$)

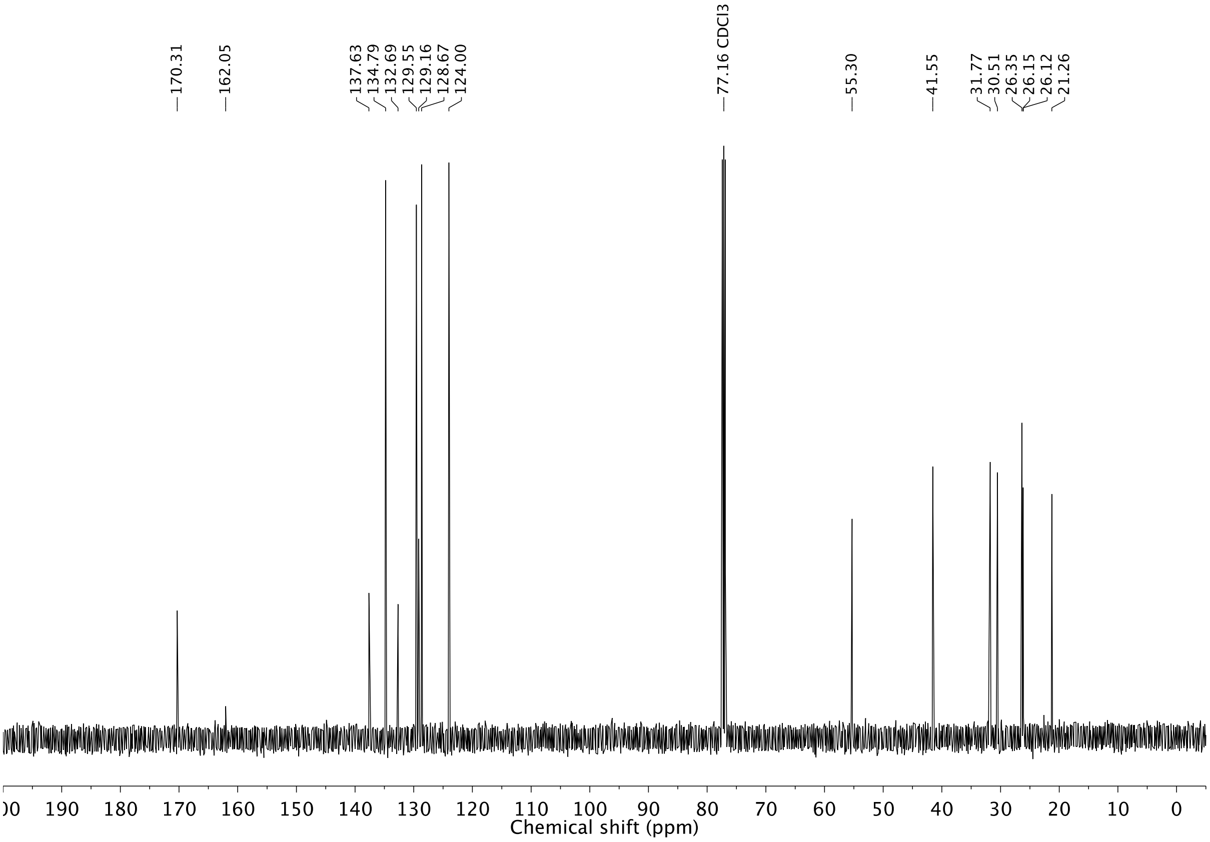

Methyl 4-(1-cyclohexyl-2-((1,3-dioxoisoindolin-2-yl)oxy)-2-oxoethyl)benzoate 5h

^{1}H NMR (500 MHz, $CDCl_3$)

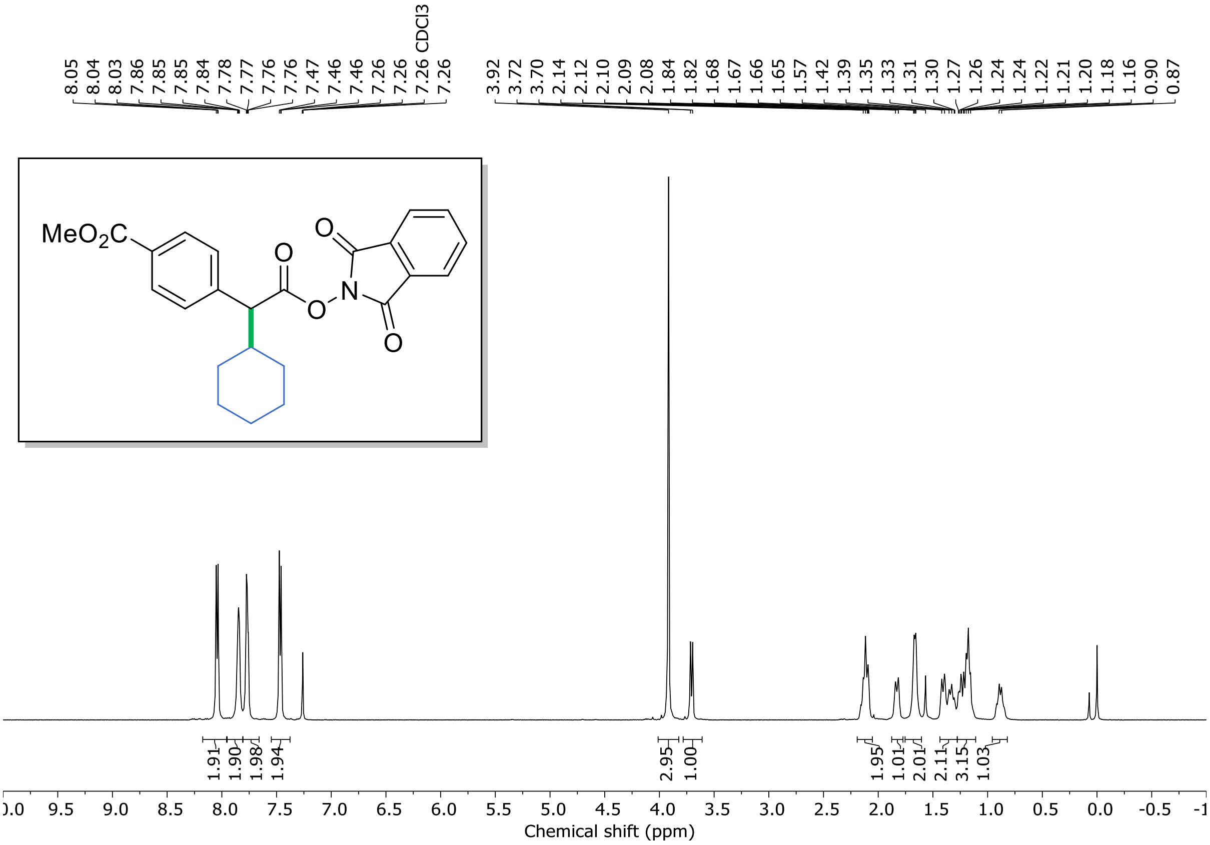

^{13}C NMR (126 MHz, $CDCl_3$)

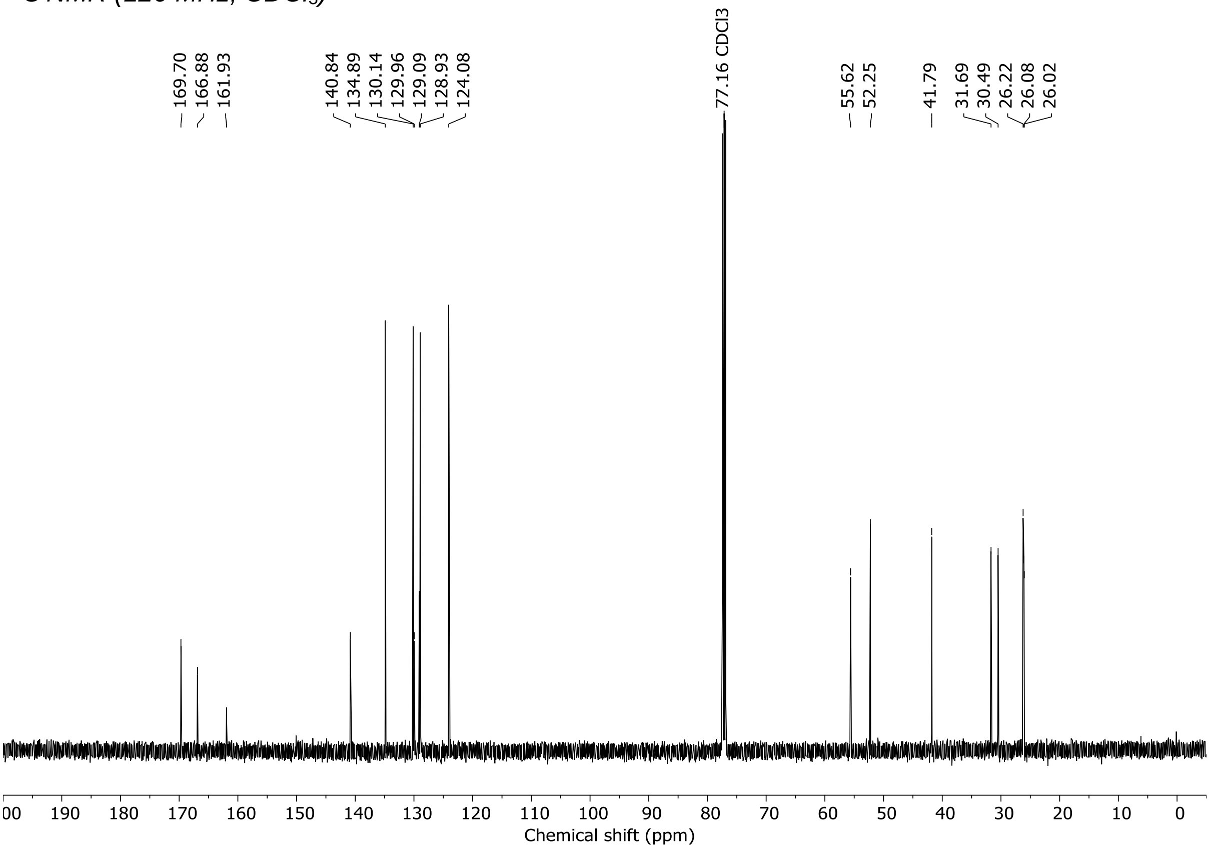

1,3-dioxoisoindolin-2-yl acrylate 6a

^{1}H NMR (500 MHz, $CDCl_3$)

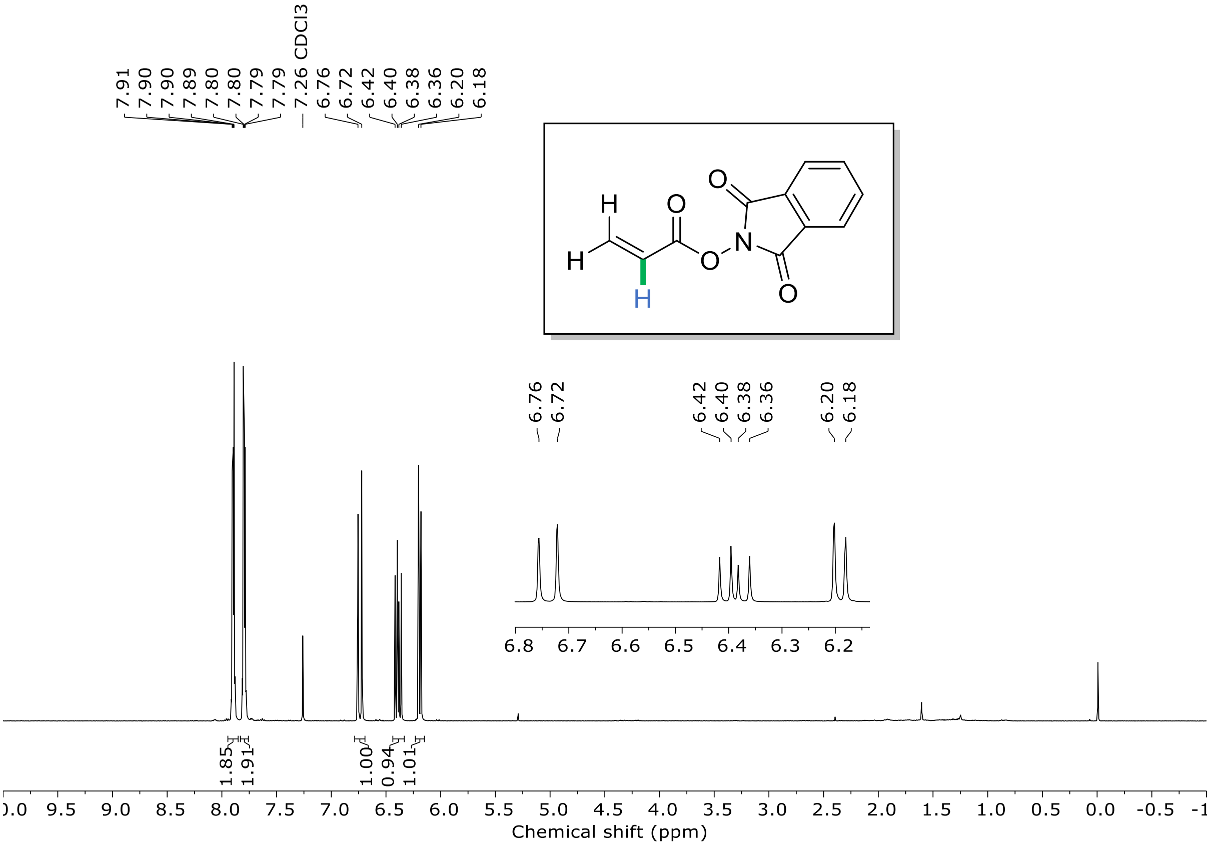

^{13}C NMR (126 MHz, $CDCl_3$)

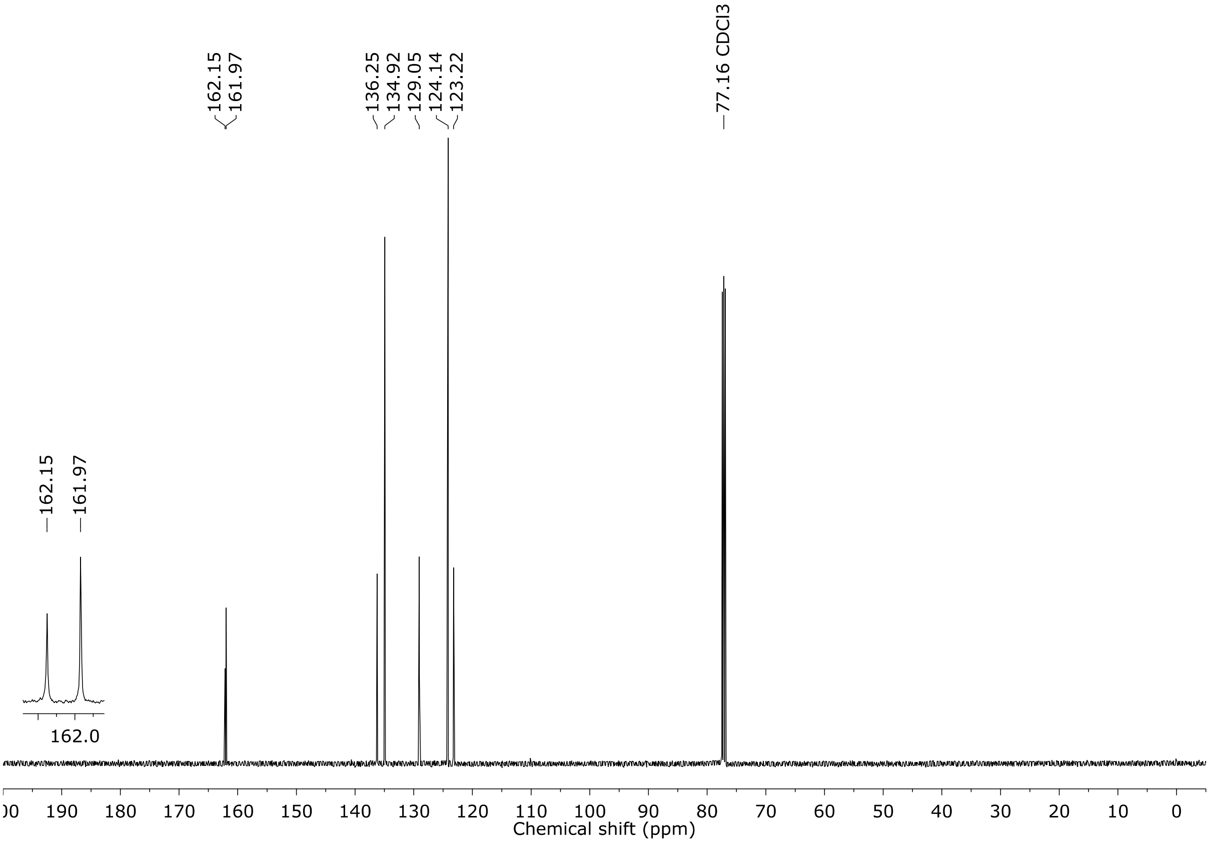

1,3-dioxoisoindolin-2-yl but-2-enoate 6b

^{1}H NMR (500 MHz, CDCl$_3$)

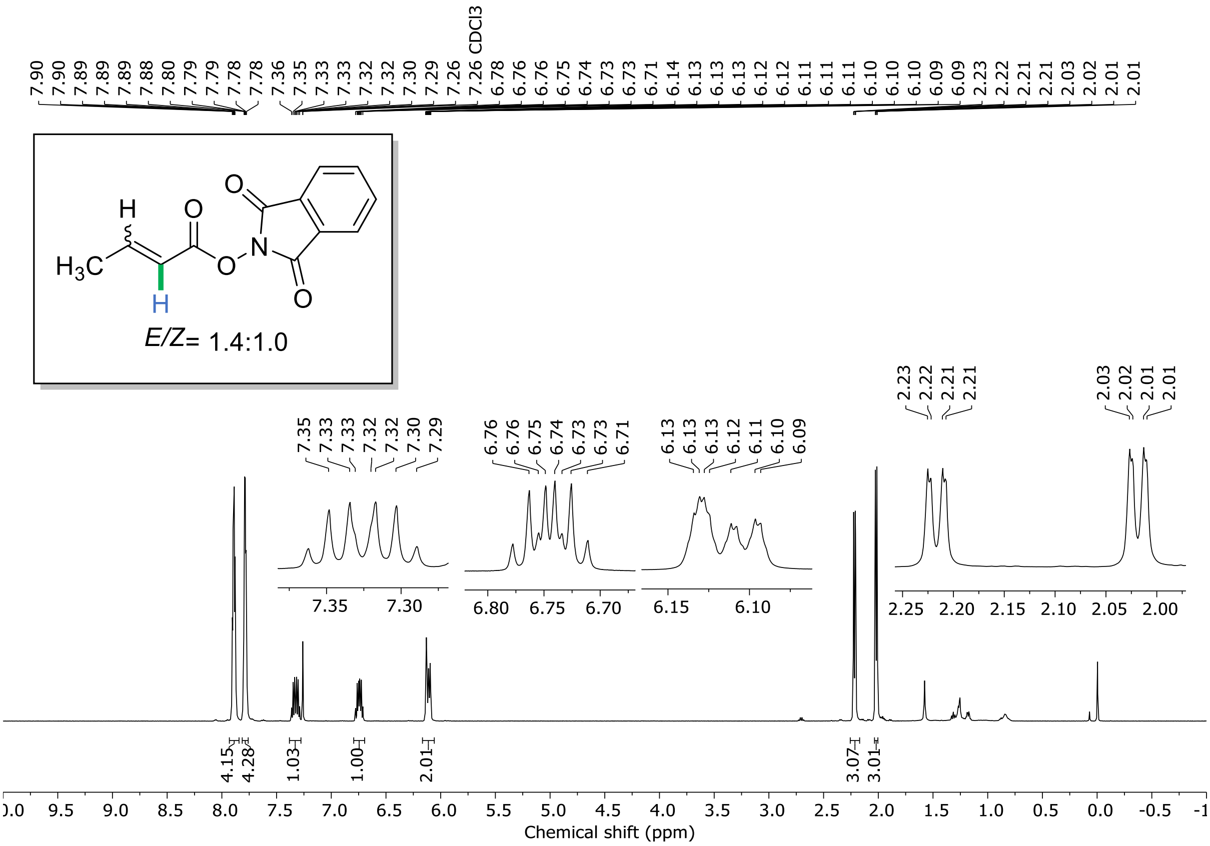

^{13}C NMR (126 MHz, CDCl$_3$)

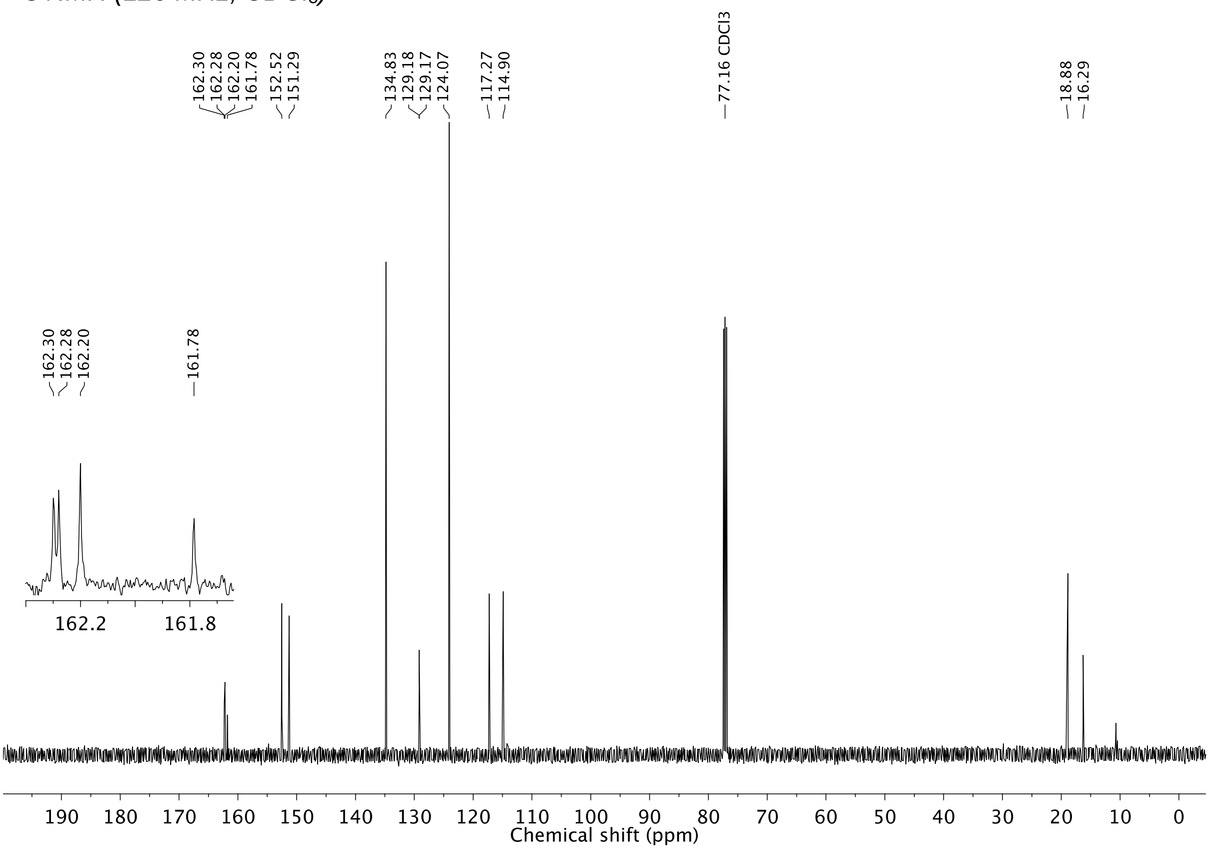

1,3-dioxoisoindolin-2-yl 3-methylbut-2-enoate 6c

^{1}H NMR (500 MHz, $CDCl_3$)

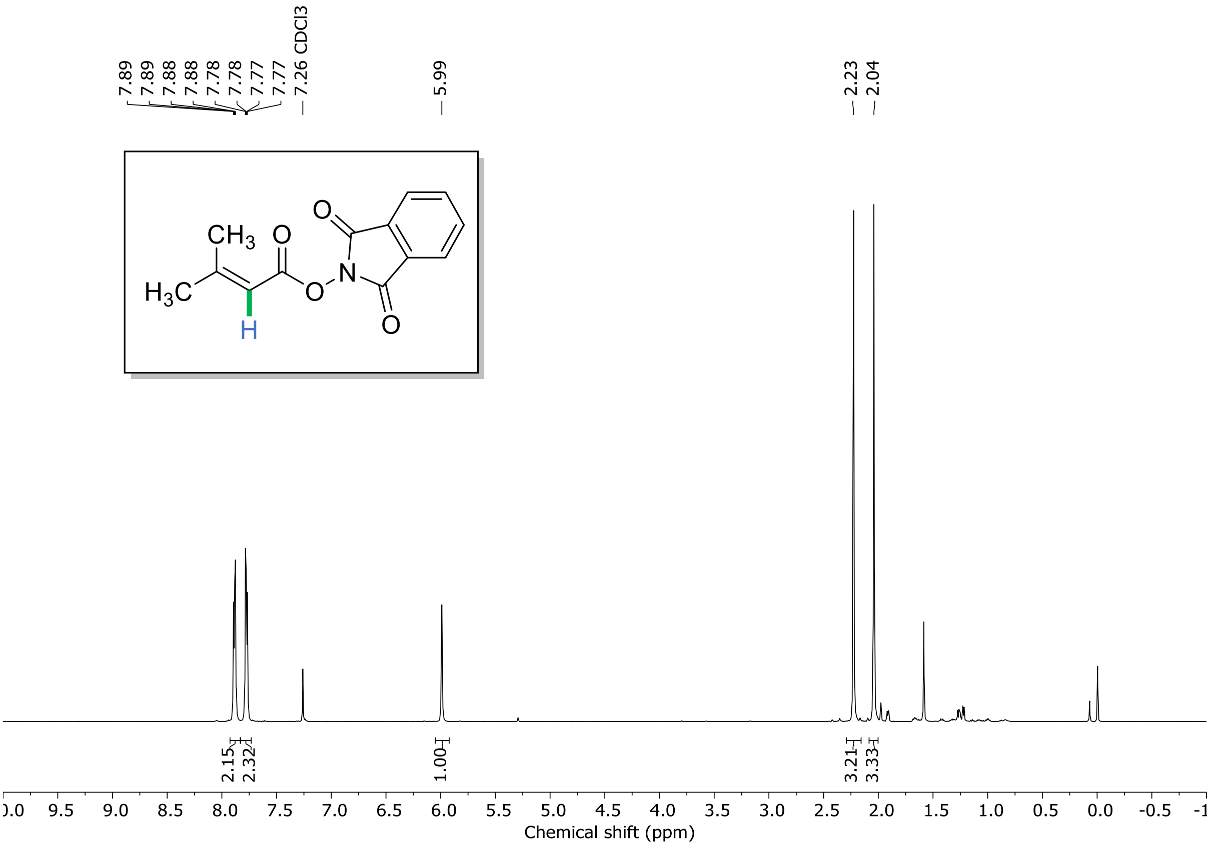

^{13}C NMR (126 MHz, $CDCl_3$)

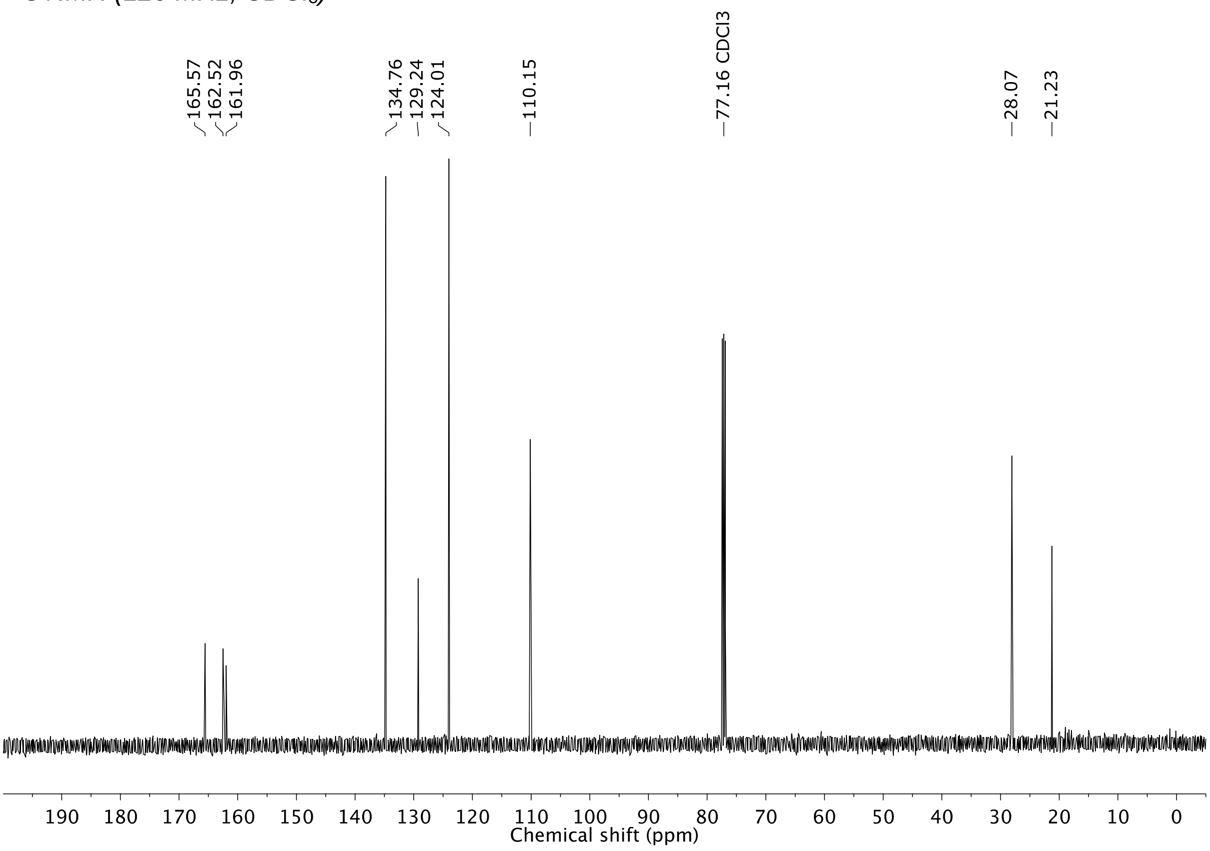

1,3-dioxoisoindolin-2-yl 2-cyclohexylideneacetate 6d

^{1}H NMR (500 MHz, $CDCl_3$)

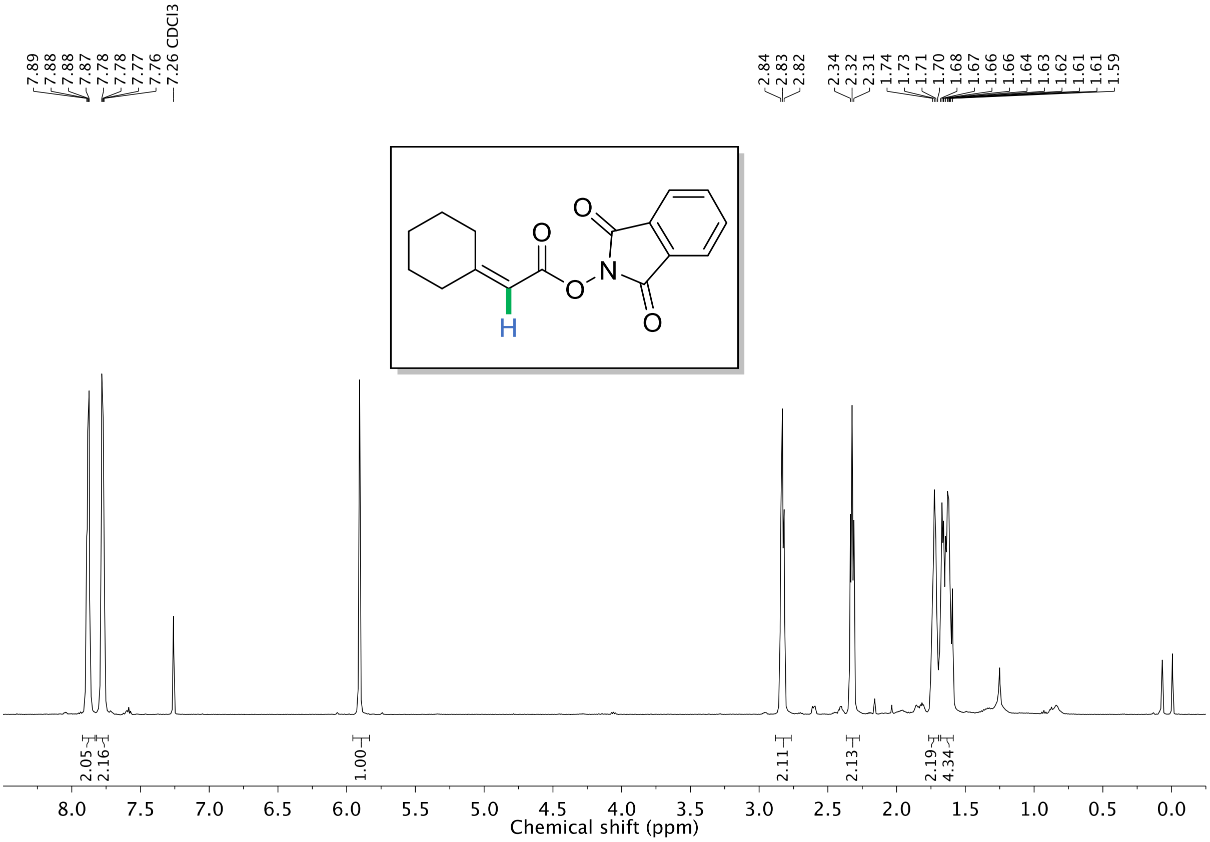

^{13}C NMR (126 MHz, $CDCl_3$)

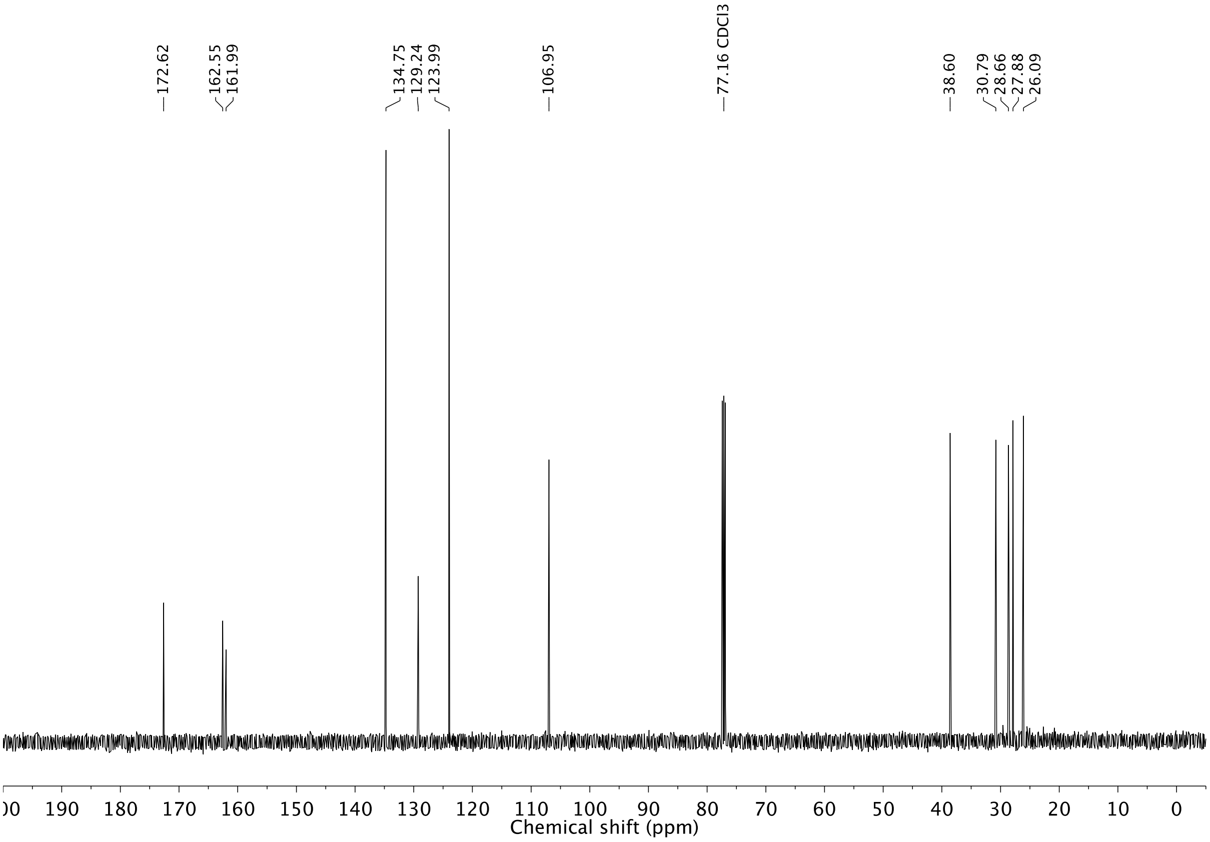

1,3-dioxoisoindolin-2-yl 2,3-dimethylbut-2-enoate 6e

^1H NMR (500 MHz, $CDCl_3$)

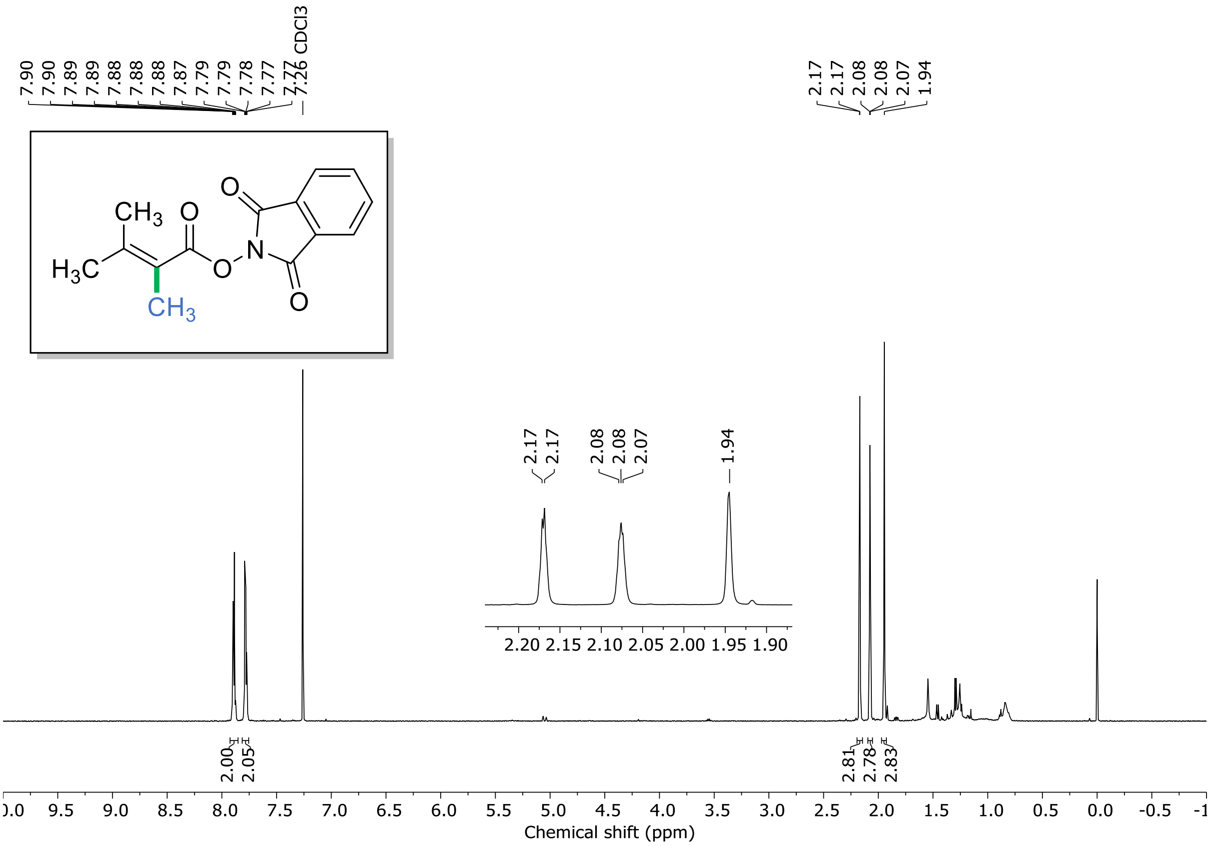

^{13}C NMR (126 MHz, $CDCl_3$)

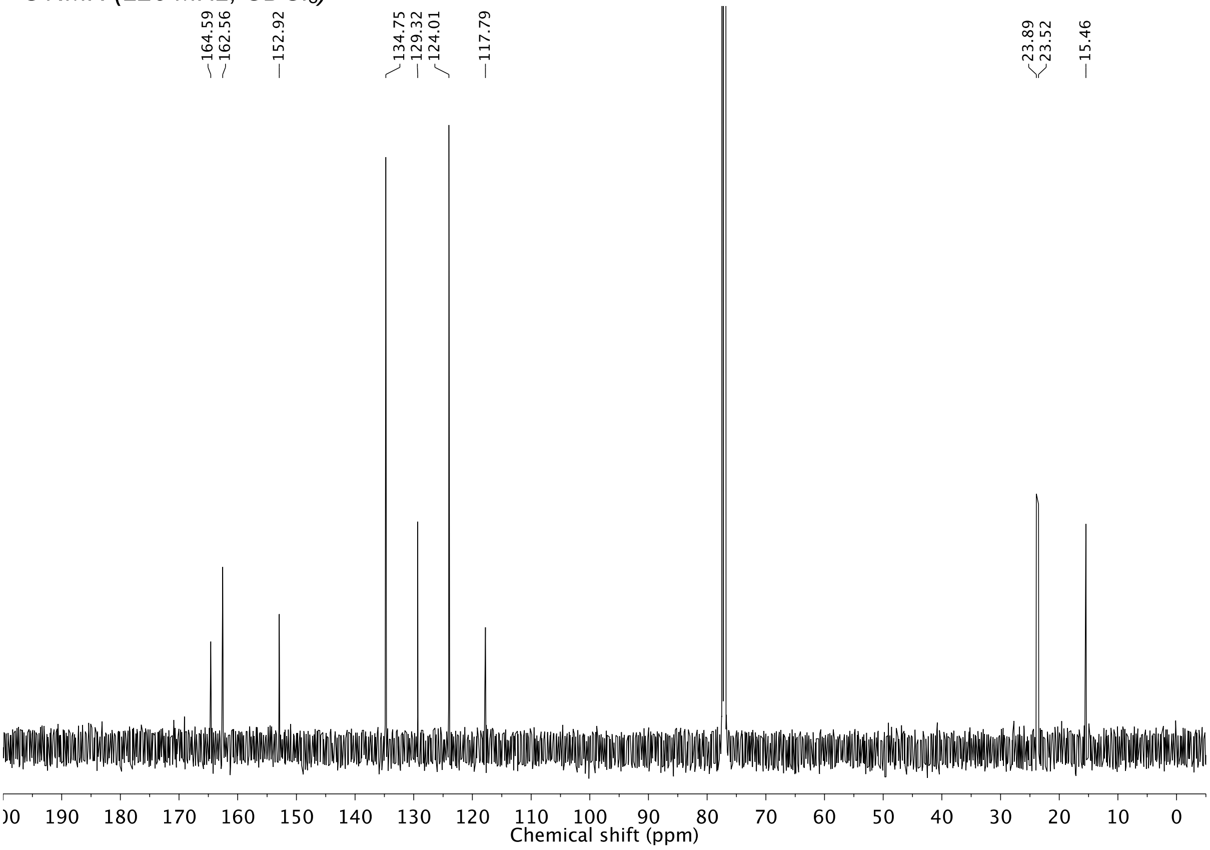

1,3-dioxoisoindolin-2-yl 2,3-dimethylpent-2-enoate (6fa) and 1,3-dioxoisoindolin-2-yl 2-ethyl-3-methylbut-2-enoate (6fb)

^{1}H NMR (500 MHz, $CDCl_3$)

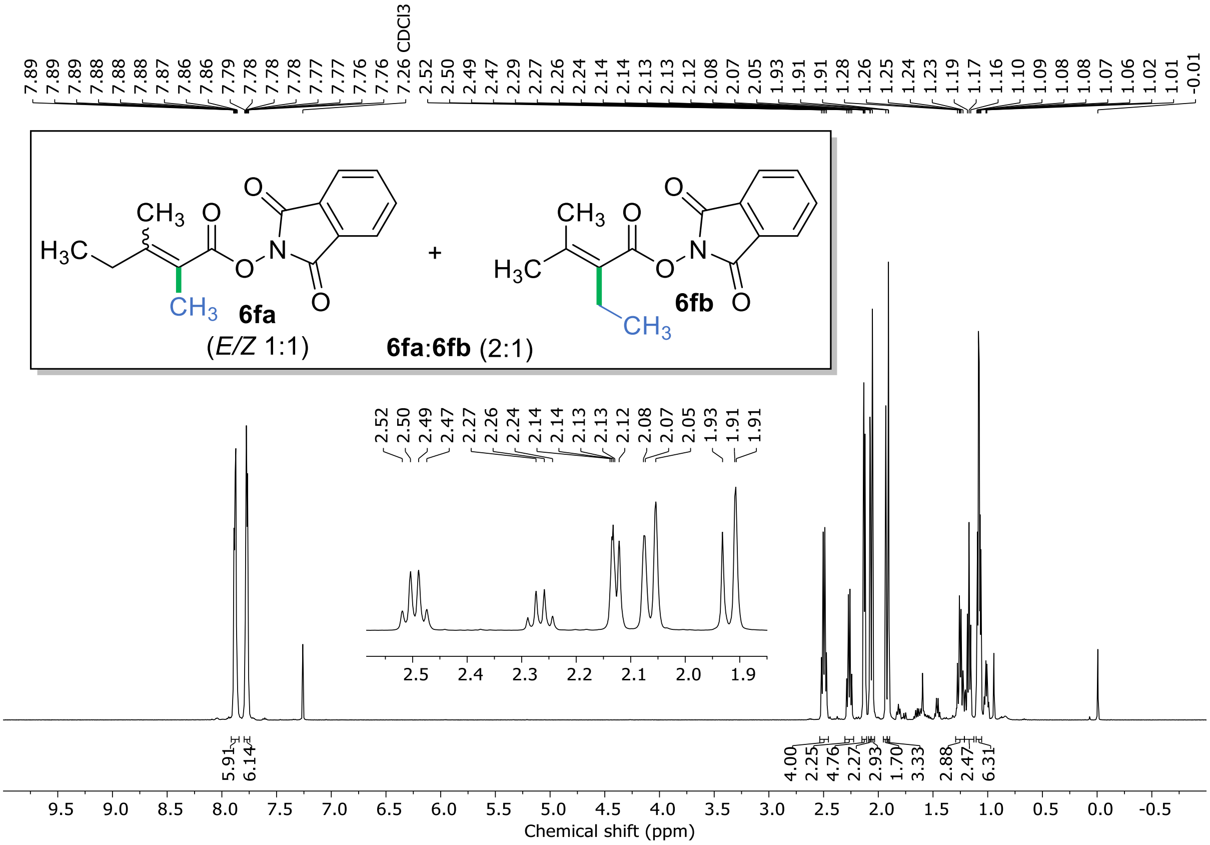

^{13}C NMR (126 MHz, $CDCl_3$)

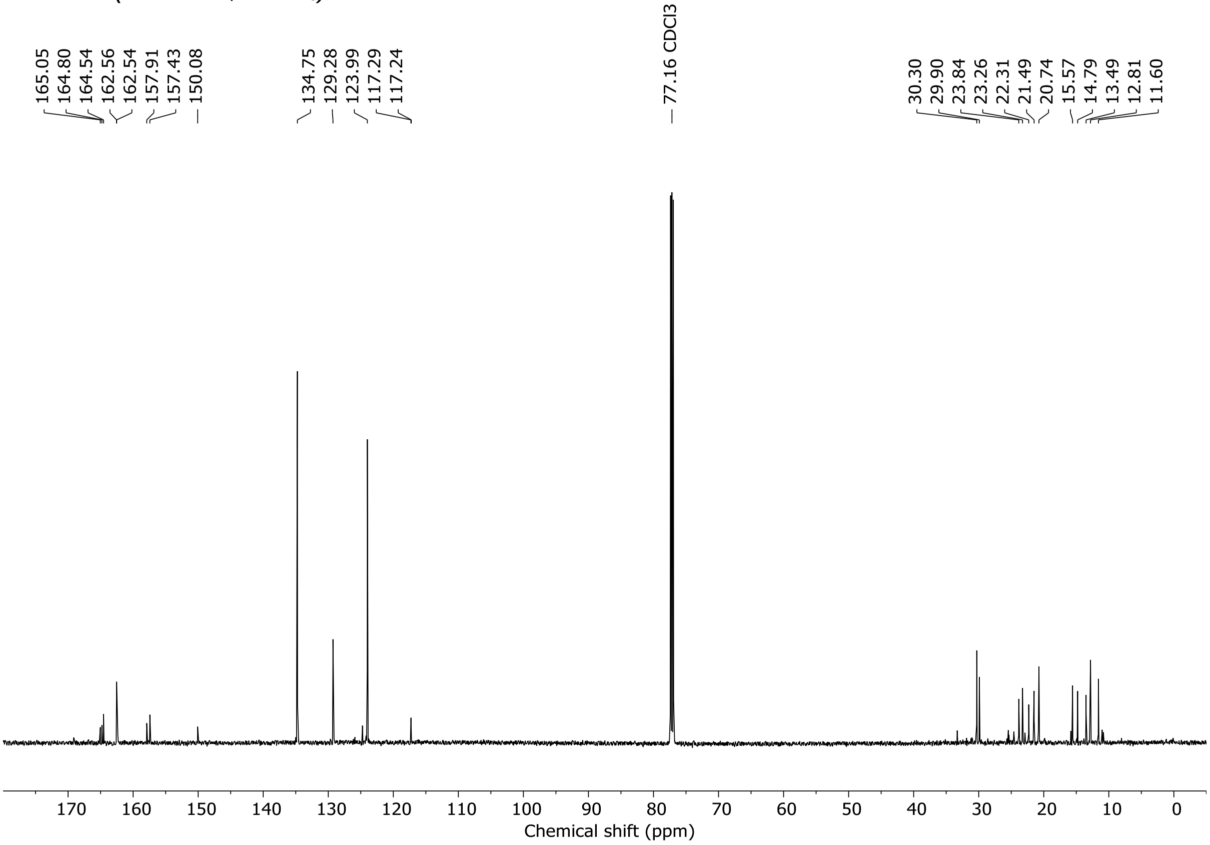

2-((1,3-dioxoisoindolin-2-yl)oxy)-2-oxo-1-phenylethyl benzoate 7

^{1}H NMR (500 MHz, $CDCl_3$)

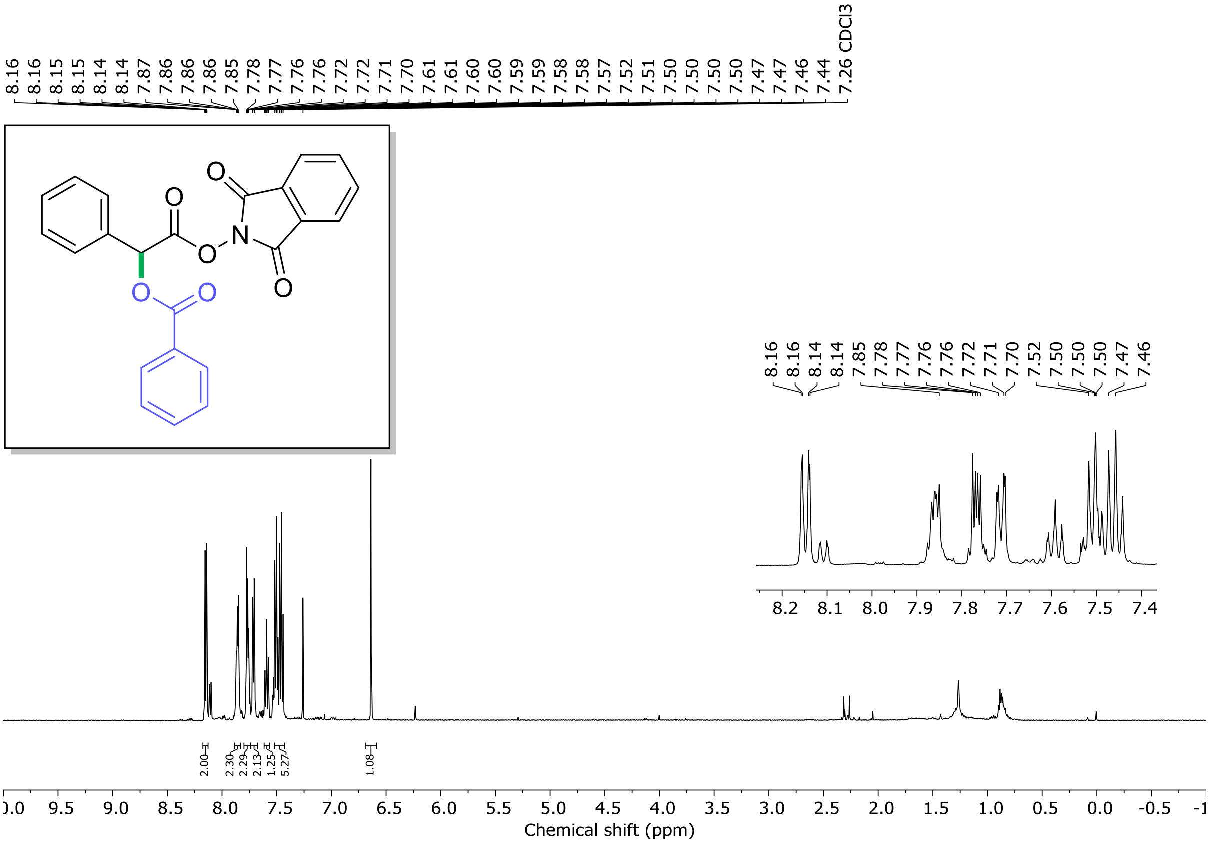

^{13}C NMR (126 MHz, $CDCl_3$)

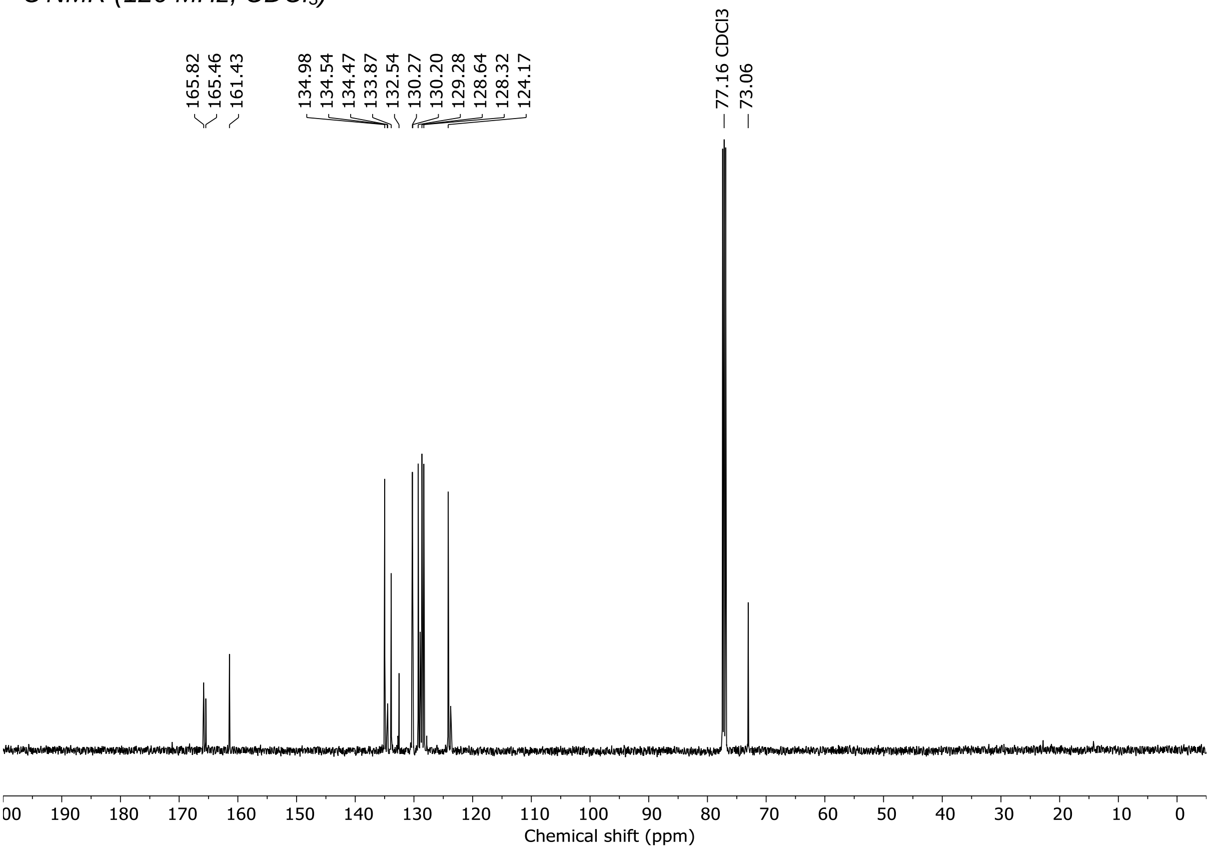

Supplement: Supplementary file 1 — ol3c02055_si_001.pdf [file ol3c02055_si_001.pdf]
